# Mitochondrial OXPHOS restricts SARS-CoV-2 replication

**DOI:** 10.1126/sciadv.adz3081

**Published:** 2026-06-03

**Authors:** Yentli E. Soto Albrecht, Ryan M. Morrow, Devin Kenney, Arnold Z. Olali, Alan Wacquiez, Nader Chehadeh, Zimu Cen, Jeffrey A. Haltom, Ian Chen, Sujata S. Ranshing, Gabrielle A. Widjaja, Alessia Angelin, Jesus A. Tintos-Hernandez, Wanqing Xie, Prasanth Potluri, Marie T. Lott, Shiping Zhang, Mohsan Saeed, Deborah G. Murdock, Susan R. Weiss, Florian Douam, Douglas C. Wallace

**Affiliations:** ^1^ Center for Mitochondrial and Epigenomic Medicine, Children’s Hospital of Philadelphia, Philadelphia, PA, USA.; ^2^ Department of Microbiology, Perelman School of Medicine, University of Pennsylvania, Philadelphia, PA, USA.; ^3^ Department of Virology, Immunology and Microbiology, Boston University Chobanian and Avedisian School of Medicine, Boston, MA, USA.; ^4^ National Emerging Infectious Diseases Laboratories, Boston University, Boston, MA, USA.; ^5^ Department of Biochemistry and Cell Biology, Boston University Chobanian and Avedisian School of Medicine, Boston, MA, USA.; ^6^ Department of Pathology and Laboratory medicine, Boston University Chobanian and Avedisian School of Medicine, Boston, MA, USA.; ^7^ Department of Bioengineering, University of Pennsylvania, Philadelphia, PA, USA.; ^8^ Department of Biomedical and Health Informatics, Children’s Hospital of Philadelphia, Philadelphia, PA, USA.; ^9^ Division of Human Genetics, Department of Pediatrics, Perelman School of Medicine, University of Pennsylvania, Philadelphia, PA, USA.

## Abstract

Severe acute respiratory syndrome coronavirus 2 (SARS-CoV-2) rewires host metabolism to optimize virus production. Although glycolysis is necessary for virus production, the importance of mitochondrial oxidative phosphorylation (OXPHOS) is unknown. The mitochondrial DNA (mtDNA) codes for 13 critical OXPHOS polypeptides plus the 22 transfer RNAs (tRNAs) and 2 ribosomal RNAs (rRNAs) for mitochondrial protein synthesis. We found an ∼5- to 100-fold greater SARS-CoV-2 virus production in infected human ACE2-expressing A549 lung cells when OXPHOS was inhibited by mtDNA depletion (ρ^0^ cells), inhibition of mitochondrial translation with chloramphenicol (CAP), or chemical inhibition of OXPHOS complexes. OXPHOS inhibition led to a marked increase in the size and distribution of viral replication centers and accelerated the production and release of infectious particles, occurring ∼2 hours earlier than in parental A549-ACE2 (wild type) cells. Subsequently, we found that increased glycolytic capacity was required for enhanced viral replication whereas differences in innate immune pathway activation were not. Reintroduction of mtDNA from a well-defined maternal lineage into the ρ^0^ cells reinstated OXPHOS, impaired SARS-CoV-2 replication, and reversed associated viral and glycolytic correlates. Thus, metabolic balance regulates SARS-CoV-2 replication, with OXPHOS exerting an antiviral effect.

## Teaser

The balance between OXPHOS and glycolysis dictates SARS-CoV-2 replication, where OXPHOS is antiviral.

## INTRODUCTION

The severe acute respiratory syndrome coronavirus 2 (SARS-CoV-2) pandemic has caused over 750 million COVID-19 cases (*1*), resulting in more than 7 million deaths globally (*1*, *2*). Long-lasting postviral syndromes (Long Covid) and deaths continue as derivative SARS-CoV-2 variants arise that evade vaccine-elicited immunity and compromise the efficacy of existing antiviral therapies (*3*, *4*). SARS-CoV-2 has an ∼30-kb positive sense genome that encodes four structural proteins [envelope (E), nucleocapsid (N), spike (S), and membrane (M)] and 16 nonstructural proteins (nsp1 to nsp16), which, respectively, incorporate into the virion and replicate and transcribe the viral genome, and nine accessory genes, which contribute to viral virulence (*5*–*7*). SARS-CoV-2 belongs to the family *Coronaviridae*, genus *Betacoronavirus*, and subgenus sarbecoviruses and is one of the seven coronaviruses that cause disease in humans. Three coronaviruses cause severe respiratory infections [SARS-CoV-2, SARS-CoV, and Middle East respiratory syndrome coronavirus (MERS-CoV)], and four coronaviruses cause the common cold (HCoV-229E, HCoV-NL63, HCoV-OC43, and HCoV-HKU1) (*7*–*9*). Common cold coronaviruses lack the accessory immune antagonists expressed by the lethal coronaviruses including SARS-CoV-2 (*10*), although all coronaviruses remodel the host endoplasmic reticulum (ER) to establish viral replication organelles or double membrane vesicles (DMVs). DMVs contain viral replication-transcription complexes identifiable as foci of double-stranded RNA (dsRNA), which protect dsRNA from innate immune sensing (*11*–*13*). Coronavirus replication has been linked to extensive host metabolic remodeling, although the role of oxidative phosphorylation (OXPHOS) in viral replication and DMV formation remains unknown. Because severe COVID-19 is linked to higher virus production (*14*–*16*), modulating host metabolism to restrict SARS-CoV-2 replication presents a promising antiviral strategy.

Cells (and the viruses that infect them) derive energy from two balanced pathways: cytosolic glycolysis and mitochondrial OXPHOS. Glycolysis is more anabolic and generates adenosine triphosphate (ATP) while preserving carbon substrates for use in biogenesis, whereas OXPHOS catabolizes the carbon backbones to maximize ATP production. The differential regulation of glycolysis and OXPHOS is critical to driving altered cell states like proliferation, cancer, and, recently, viral infection (*17*–*19*). Glycolysis is up-regulated by most viruses, whereas viral effects on OXPHOS are variable (*20*). SARS-CoV-2 up-regulates glycolysis, and down-regulates OXPHOS by inhibiting the transcription of both nuclear DNA (nDNA)–coded and mitochondrial DNA (mtDNA)–coded OXPHOS genes, which contribute to large polypeptide OXPHOS complexes (*13*, *21*–*25*). The mtDNA is a small, circular, multicopy DNA coding for 13 essential OXPHOS polypeptides: 7 of 45, 1 of 11, 3 of 13, and 2 of 17 polypeptides, for OXPHOS complexes I, III, IV, and V, respectively. The mtDNA also encodes 22 tRNAs and 2 ribosomal RNAs (rRNAs) for mitochondrial protein synthesis, which translates the 13 mtDNA polypeptides (*26*, *27*). The nDNA codes for ∼160 additional OXPHOS genes. In OXPHOS, electrons from substrates flow through complexes I, III, and IV of the electron transport chain (ETC) to create a proton gradient used by complex V to generate ATP (*28*). Inhibition of OXPHOS complex activity redirects ETC electrons to O_2_ to generate mitochondrial reactive oxygen species (mROS) (*29*), and mROS stabilizes hypoxia-inducible factor (HIF-1α) transcription factor, which up-regulates glycolytic gene expression (*30*). Suppressed OXPHOS gene expression results in an imbalance between nDNA and mtDNA OXPHOS polypeptides, activating the mitochondrial unfolded protein response (UPR^MT^), which is also linked to increased glycolysis (*31*, *32*).

Inhibition of mROS, HIF-1α, and glycolysis limits SARS-CoV-2 replication and pathology (*33*–*37*), yet the direct effects of OXPHOS inhibition on SARS-CoV-2 virus production are unknown. It has been reported that SARS-CoV-2 RNA intermediates localize within the mitochondrial matrix, as observed by both fluorescence and immunoelectron microscopy (*24*, *38*, *39*). Others have proposed that SARS-CoV-2 may use mitochondrial translation to generate certain viral peptides (*39*–*42*) and that chloramphenicol (CAP), an inhibitor of mitochondrial translation, may inhibit the SARS-CoV-2 papain-like protease (*43*, *44*). If either are correct, CAP should inhibit SARS-CoV-2 virus production.

Because mitochondria are central to several immune responses that restrict SARS-CoV-2 infection, several forms of mitochondrial dysfunction have the potential to impair mitochondrial inflammation. Mitochondrially released mtDNA and mitochondrial double-stranded RNA (mtRNA), along with viral dsRNA, initiate innate immune responses (*45*, *46*). mtDNA activates the NOD-like receptor protein 3 (NLRP3) inflammasome, which generates interleukin-1β (IL-1β) and triggers gasdermin D pores and cell death (*47*–*49*). mtDNA activates the cyclic GMP-AMP synthase–stimulator of interferon genes (cGAS-STING) and Toll-like receptor 9 (TLR9) pathways and melanoma differentiation-associated gene 5 (MDA-5), which detects mtRNA and viral dsRNA. cGAS-STING and TLR9 induce tumor necrosis factor–α (TNFα) via nuclear factor κB (NF-κB) pathway activation as well as interferons (IFNs). IFNs establish an autocrine and paracrine antiviral program, initiating the transcription of hundreds of IFN-stimulated genes (ISGs), where both type I (IFN-α and IFN-β) and type III (IFN-λ) IFNs are important restrictors of SARS-CoV-2 replication (*50*). Mitochondrial antiviral signaling protein (MAVS) is an nDNA-expressed mitochondrial protein residing on the outer mitochondrial membrane that forms an oligomer and signalosome upon binding activated MDA-5, a major pathway for IFN production in nonimmune cells during SARS-CoV-2 and other coronavirus infections. These immune players interface with mitochondrial function and decrease SARS-CoV-2 replication when active (*51*–*58*).

**Fig. 1.**
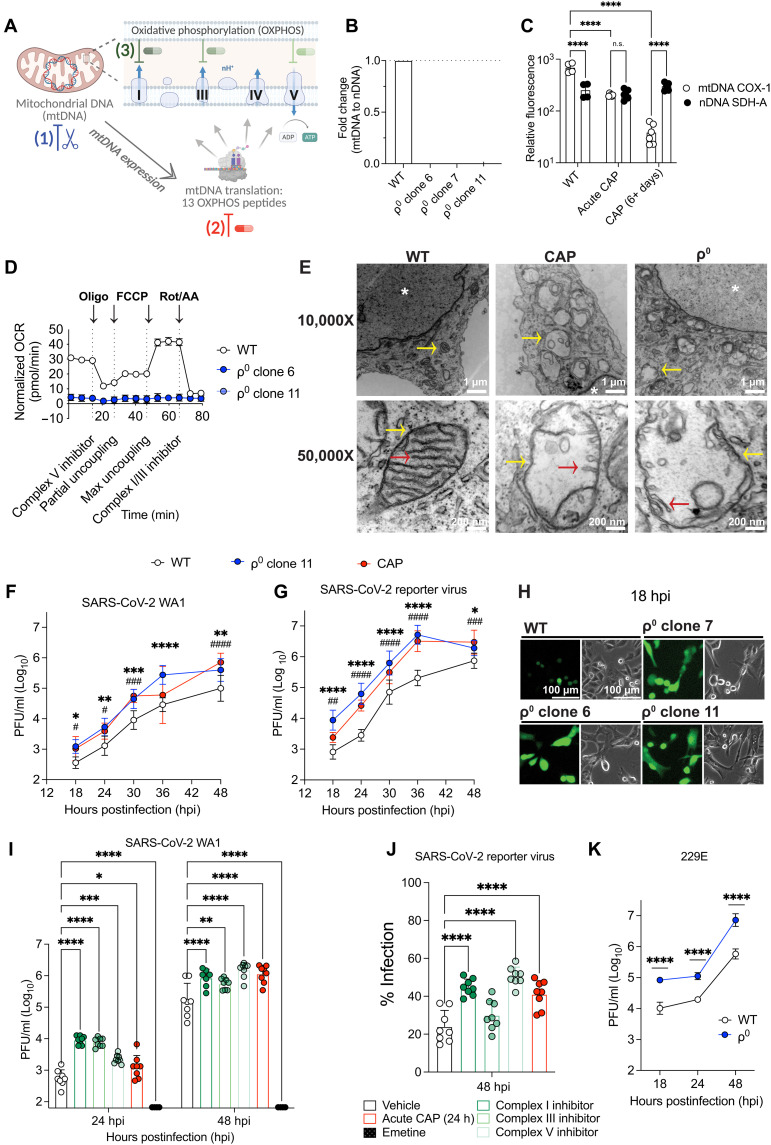
OXPHOS restricts SARS-CoV-2 replication. (**A**) OXPHOS inhibited by mtDNA depletion, CAP, or chemical inhibitors. Created with BioRender. Y. Soto Albrecht (2026) https://BioRender.com/5kz25jo. (**B**) qPCR of mtDNA *ND5* to nDNA *GAPDH* graphed as fold change (2^−ΔΔCT^) over WT *n* = 3 experiments. (**C**) mtDNA (COX-1) and nDNA (SDH-A) mitochondrial protein levels from A549-ACE2 cells treated with CAP (50, 100, and 200 μg/ml) for five passages (CAP) or 24 hours (acute CAP). Two- to 3-well replicates, representative of *n* = 4 with CAP (50 μg/ml). (**D**) DAPI+ (4′,6-diamidino-2-phenylindole–positive) nuclei-corrected OCR post–mitochondrial stress test Seahorse assay. Rot, rotenone; Oligo, oligomycin; antimycin A (AA); FCCP, carbonyl cyanide *p*-trifluoromethoxyphenylhydrazone. Ten-well replicates, representative of *n* = 3. (**E**) ρ^0^ cells and CAP cells show an absence of mitochondria (yellow arrow) with a typical inner membrane folding, or cristae (red arrow), by TEM. The nucleus is denoted by a white asterisk (*). Two-well replicates, *n* = 1. (**F** to **K**) Cells were infected with the SARS-CoV-2 WA1 (F and I), SARS-CoV-2 NeonGreen reporter (G, H, and J), or alphacoronavirus 229E (K), at an MOI of 0.2, and viral replication was assessed by supernatant plaque assay (F, G, I, and K), percent infection (J), or representative images (H). h, hours. For select experiments (I and J), complex inhibitors, CAP, emetine, or vehicle was included in the viral refeed media. Two- to 4-well replicates, *n* = 2 (F to H and J) and selected time points confirmed *n* = 3 (F to H) or *n* = 2 of 3 (I and K). Means ± SD where applicable. Where statistics are shown, a two-way ANOVA was performed compared to WT or vehicle where *P < 0.05 or ^#^*P* < 0.05; **P < 0.01 or ^##^*P* < 0.01; ***P < 0.001 or ^###^*P* < 0.001; ****P < 0.0001. In (F) and (G), *: ρ^0^ and #: CAP. In (C), mtDNA compared to nDNA within each condition. Image brightness and contrast adjusted simultaneously for visibility.

To determine the role of OXPHOS in SARS-CoV-2 pathogenesis, we inhibited OXPHOS by three parallel approaches in the human lung cancer cell line A549 expressing the SARS-CoV-2 receptor, angiotensin converting enzyme (A549-ACE2). We inhibited the expression of mtDNA-coded polypeptides by removing the mtDNA from A549-ACE2 cells (ρ^0^ cells) (*59*–*65*) or by blocking mitochondrial translation with prolonged CAP treatment. OXPHOS activity was inhibited by treating the cells with specific ETC and ATP synthase (complex V) inhibitors, and this is summarized in the Fig. 1A schematic. Instead of suppressing SARS-CoV-2 propagation, these treatments enhanced viral production. Notably, mitochondria-associated inflammation remained intact, whereas OXPHOS inhibition enhanced glycolysis, which was required for increased viral replication. Hence, OXPHOS exerts a metabolically regulated antiviral effect on SARS-CoV-2 replication. Our findings may offer mechanistic insight into new and existing host-directed antiviral strategies against acute and Long Covid.

## RESULTS

### Inhibition of OXPHOS modulates mitochondrial structure and function

To determine whether OXPHOS is required for SARS-CoV-2 replication, we inhibited OXPHOS in A549-ACE2 cells by three parallel and independent approaches, followed by infection with SARS-CoV-2. A549-ACE2 ρ^0^ cells were created by depletion of their mtDNA by serial passage in low-dose ethidium bromide (50 ng/ml) followed by clonal selection. mtDNA depletion was confirmed in three ρ^0^ clones by showing that mtDNA-coded NADH dehydrogenase subunit 5 gene (*MT-ND5*) of complex I was undetectable by quantitative polymerase chain reaction (qPCR) (Fig. 1B). Western blots confirmed that ACE2 was still expressed in the ρ^0^ cells (fig. S1A). Mitochondrial translation was inhibited by serial passage in media containing CAP (50 μg/ml). CAP selectively blocks the synthesis of mtDNA-coded polypeptides including cytochrome c oxidase (complex IV) subunit 1 (COXI) but not the nDNA-coded OXPHOS polypeptide, succinate dehydrogenase complex II subunit A (SDH-A) (Fig. 1C). All measures of OXPHOS use oxygen consumption rate (OCR), or “mitochondrial respiration,” as a proxy for OXPHOS activity (*66*). A549-ACE2 OXPHOS capacity was reduced to 60% when cells were maintained in CAP for 24 hours and to ρ^0^ levels when cells were exposed to CAP for 6 days or more (fig. S1B). Loss of OXPHOS in ρ^0^ cells was confirmed by the absence of mitochondrial respiration (Fig. 1D). OXPHOS I, III, and V complexes were inhibited using rotenone, antimycin A, and oligomycin, respectively. These drugs inhibit A549-ACE2 respiration within minutes (Fig. 1D).

Transmission electron microscopy (TEM) revealed that mtDNA depletion in ρ^0^ cells and inhibition of mitochondrial translation by CAP resulted in loss of mitochondrial cristae, in association with disrupted assembly of complex V (*67*) (Fig. 1E). Suppression of OXPHOS in the ρ^0^ and CAP cells resulted in increased cell size and altered mitochondrial patterning, from puncta to larger and more diffusely packed lobules (Fig. 1E and fig. S1C).

### Inhibition of OXPHOS enhances SARS-CoV-2 replication

Inhibition of OXPHOS in A549-ACE2 cells by mtDNA elimination (ρ^0^), CAP inhibition of mitochondrial translation, or OXPHOS complex inhibition all enhanced SARS-CoV-2 USA-WA1/2020 isolate (WA1) replication. Infection of three independent ρ^0^ clones [multiplicity of infection (MOI) = 0.2 plaque-forming units (PFU) per cell], followed by titration of infectious virus by plaque assay from cell culture media at various times postinfection, revealed that SARS-CoV-2 virus production was increased ∼10- to 100-fold in all ρ^0^ clones compared to parental A549-ACE2 cells with their endogenous mtDNA [wild type (WT)] with *P* values <0.0001 to 0.0117 (Fig. 1F and fig. S1D). Likewise, treatment of A549-ACE2 cells with CAP increased viral titers ∼10-fold between 18 and 48 hours postinfection (hpi) (Fig. 1F and fig. S1D). Increased viral propagation was confirmed by plaque assay following infection with a SARS-CoV-2 NeonGreen reporter strain (*68*) (Fig. 1G and fig. S1E) for which reporter gene fluorescence increased faster, with greater intensity and percent infection in ρ^0^ cells than in WT cells (Fig. 1, G and H, and fig. S1, E and F). In contrast to CAP stimulation of SARS-CoV-2 replication, inhibition of cytosolic translation with emetine blocked SARS-CoV-2 virus production (Fig. 1I). Hence, inhibition of mitochondrial translation strongly enhanced SARS-CoV-2 infectious particle production, whereas inhibition of cytosolic translation blocked viral biogenesis.

To determine whether the proviral effect of OXPHOS inhibition was a product of impairing mitochondrial biogenesis versus bioenergetics, complex I was blocked with rotenone, complex III with antimycin A, and complex V with oligomycin and the effect on virus production assessed. All three inhibitors increased SARS-CoV-2 replication ∼5- to 10-fold and percent infection, despite drug-induced cytotoxic effects as seen by changes in bright-field morphology in mock-infected cells (Fig. 1, I and J, and fig. S1G). Hence, the proviral effect of OXPHOS inhibition is the result of inhibiting mitochondrial respiration, not mitochondrial biogenesis.

We asked whether the relationship between OXPHOS inhibition and increased SARS-CoV-2 replication holds across the *Coronaviridae* family because they share key elements of their viral life cycle. We infected three ρ^0^ clones with the common cold *Alphacoronavirus*, HCoV-229E. All three ρ^0^ clones displayed increased HCoV-229E replication ∼10-fold higher than parental WT cells (Fig. 1K and fig. S1H). Thus, the proviral effect of OXPHOS deficits holds for other coronaviruses. To determine whether the increase in viral replication associated with OXPHOS inhibition was specific for A549-ACE2 cells (*65*), we treated embryonic kidney human embryonic kidney (HEK) 293T cells with OXPHOS complex I and III inhibitors. Both increased SARS-CoV-2 viral titers in HEK293T cells at 18 hpi, complex I at 24 hpi, and both enhanced percent infection at 48 hpi (fig. S1, I to K). There was a stronger effect for complex I over III inhibition, although both increased viral replication to a lesser extent than seen for A549-ACE2 cells. In summary, OXPHOS is not required for SARS-CoV-2 replication, and instead SARS-CoV-2 virus production is enhanced ∼10- to 100-fold in OXPHOS-deficient cells.

### Transmitochondrial cybrids exhibit restored OXPHOS and cellular morphology

**Fig. 2.**
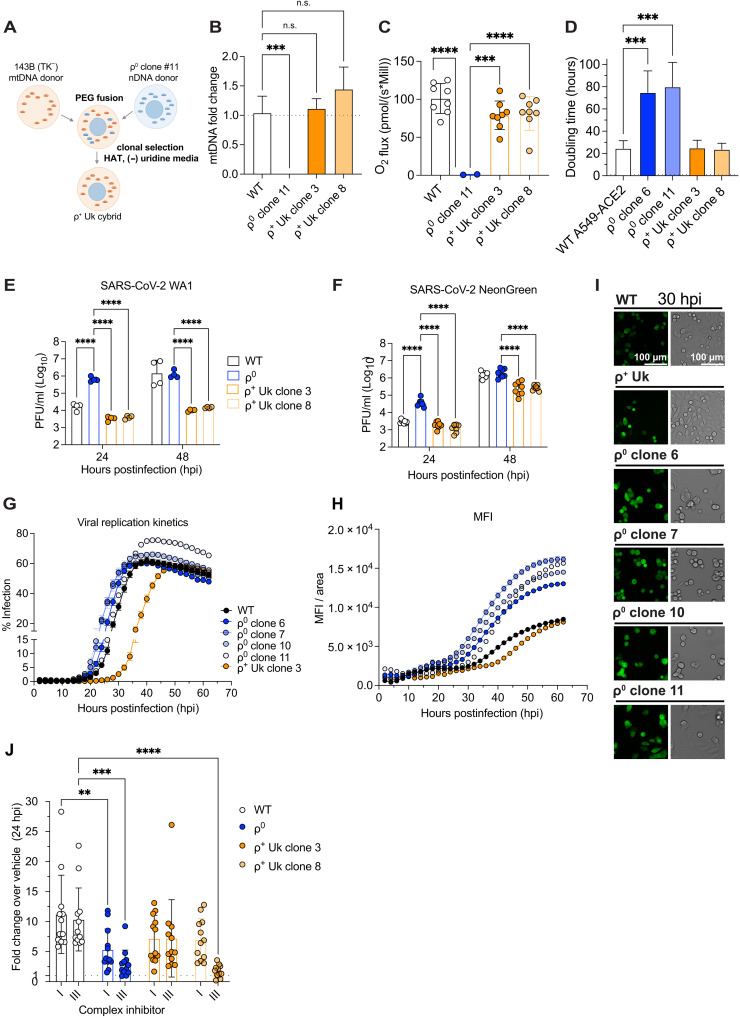
Transmitochondrial cybrids restore mitochondrial OXPHOS and revert viral replication below baseline. (**A**) Schematic of forming ρ^+^ Uk cybrids by fusing Uk mtDNA cytoplasts to A549-ACE2 ρ^0^ clone 11 followed by clonal selection. Created in BioRender. Y. Soto Albrecht (2026) https://BioRender.com/39oy225. (**B**) qPCR of mtDNA *ND5* to nDNA *GAPDH* graphed as fold change (2^−ΔΔCT^) over WT. Two to three averaged technical replicates, 3- to 4-well replicates, representative of *n* = 2. (**C**) OCR by Oroboros respirometry (maximum respiration) on permeabilized cells show ρ^+^ Uk cybrids with replenished OXPHOS activity. Mill, million cells. Two replicates, *n* = 5 (WT and ρ^+^ cybrids). (**D**) Acquisition of ρ^+^ Uk mtDNA restored the shorter doubling time to A549-ACE2 ρ^0^ cells. Three to four measurements, representative of *n* = 2. (**E** and **F**) Cells were infected with the SARS-CoV-2 WA1 (E) or NeonGreen reporter (F) at MOI = 0.2, and supernatant viral titer assessed by plaque assay. Four-well replicates, *n* = 2 (F) or *n* = 2 of 3 (E). (**G** to **I**) Cells infected with the SARS-CoV-2 NeonGreen reporter at an MOI of 0.2 had viral replication assessed by an ImageXpress Hi.AT high-content imager every 2 hours; results presented as percent infection/bright field (G), MFI normalized by infected cell area (H), or representative images (I). Four-well replicates, representative of *n* = 2. (**J**) Cells were infected with SARS-CoV-2 WA1 and OXPHOS complex inhibitors applied in the viral refeed media. Supernatant viral titers determined by plaque assay. Four-well replicates, *n* = 3. Means ± SD where applicable, except means ± SEM for (G) and (H). Where statistics are shown, a two-way ANOVA was performed compared to ρ^0^ clone 11 for (C), (E), and (F) or to WT for (B), (D), and (J) where **P < 0.01; ***P < 0.001; ****P < 0.0001; n.s., not significant. Images unmodified. See also fig. S2 and table S1.

If OXPHOS deficiency in A549-ACE2 ρ^0^ cells is proviral, then restoring OXPHOS should be antiviral. To test this hypothesis, we reestablished normal OXPHOS in the A549-ACE2 ρ^0^ cells using transmitochondrial cybrid technology. As the normal mtDNA donor, we chose the European mtDNA haplogroup Uk1a (Uk) to be able to distinguish the cybrid donor mtDNA from the resident A549-ACE2 H4a mtDNA due to differences in their polymorphisms. The Uk mtDNA, maintained on a 143B(TK^−^) nuclear background was transferred to A549-ACE2 ρ^0^ clone 11 cells by enucleating the 143B(TK^−^) ρ^+^ Uk cells and fusing the mitochondrial containing cytoplasts to the A549-ACE2 ρ^0^ cells (Fig. 2A). The resulting A549-ACE2 transmitochondrial cybrids harboring the ρ^+^ Uk mtDNA were clonally selected and two clones chosen for downstream experiments.

The cybrids were shown to harbor near-endogenous (WT) levels the ρ^+^ Uk1a (Uk) mtDNA by qPCR amplification and mtDNA sequencing, confirming that the resident mtDNA harbored the ρ^+^ Uk mtDNA polymorphisms and not the A549-ACE2 WT endogenous H4a mtDNA variants (Fig. 2B and table S1). The A549-ACE2 nuclear background of the cybrids was confirmed by the presence of four PCR amplicons of the ACE2 transgene cassette absent in the 143B(TK^−^) nucleus, expression of the ACE2 transgene in A549-ACE2 ρ^0^ cells by qPCR and Western blot, and chromosome count; specifically, the Uk ρ^+^ cybrids had the chromosome number of A549-ACE2 ρ^0^ cells but not those of the 143B(TK^−^) mtDNA donor (fig. S2, A to G).

The two ρ^+^ Uk cybrid clones had a restored respiratory capacity and doubling time of ∼24 hours, similar to that of WT A549-ACE2 parental cells (Fig. 2, C and D, and fig. S2D). ρ^+^ Uk cybrid cells expressed the ACE2 receptor (fig. S2, F and G) and exhibited the same compact mitochondrial puncta of WT A549-ACE2 cells by MitoTracker staining compared to the diffused mitochondrial pattern of the ρ^0^ cells they were derived from (fig. S2H). The ρ^+^ Uk cybrid nuclear morphology was lobular and more similar to the WT nucleus by two approaches to quantify differences in morphology (fig. S2, I to K). Thus, acquisition of the ρ^+^ Uk mtDNA reverted the A549-AE2 ρ^0^ subcellular structure and function back toward that of the parental A549-ACE2 WT cells (*61*, *62*, *69*–*71*).

### Cybrids restrict SARS-CoV-2 replication in an OXPHOS-dependent manner

We asked whether restoring OXPHOS capacity restricts SARS-CoV-2 replication by infecting ρ^+^ Uk cybrids and ρ^0^ and WT cells at (MOI = 0.2). Both ρ^+^ Uk cybrid clones produced ∼100-fold less infectious virus than ρ^0^ cells at 24 and 48 hpi with *P* values <0.0001 for ρ^+^ Uk clones 3 and 8 (Fig. 2, E and F). This was confirmed by the differential replication of the SARS-CoV-2 fluorescent reporter virus, which permitted tracking of viral replication kinetics (Fig. 2, G and H).

The reduction in viral replication following restoration of ρ^+^ Uk mtDNA is further supported by reduced percent infected cells as well as mean fluorescence intensity (MFI) of the NeonGreen reporter virus throughout infection (Fig. 2, H and I). The ρ^0^ cells showed increased percent and magnitude of infection over WT cells, whereas the ρ^+^ Uk cybrids showed decreased infection relative to ρ^0^ and WT cells (Fig. 2, H and I). Although the maximum percent infection was similar across samples (60 to 70%), this level of infected cells was achieved nearly 18 hours earlier in the ρ^0^ cells than in ρ^+^ Uk cybrids, demonstrating a reduction in the rate of infection progression in association with the restoration of OXPHOS (Fig. 2G). Hence, mtDNA and OXPHOS depletion in ρ^0^ cells and repletion in ρ^+^ Uk cybrids regulates SARS-CoV-2 replication, at the level of virus production and the kinetics of viral spread.

To confirm that this relationship is driven by changes in OXPHOS function, we inhibited OXPHOS complex I (rotenone) and complex III (antimycin A) in the Uk mtDNA cybrids and controls and assessed supernatant viral titers. We plotted fold change in viral titers over vehicle-treated cells to compare the increase in SARS-CoV-2 replication when OXPHOS inhibitors are applied to ρ^+^ Uk, WT, and ρ^0^ cells (Fig. 2J). Inhibition of OXPHOS complexes I and III similarly increased SARS-CoV-2 replication in ρ^+^ Uk cybrids and WT cells ∼5- to 10-fold, whereas ρ^0^ cells were minimally affected, although ρ^+^ Uk clone 8 did not respond to the antimycin A concentration used. Thus, OXPHOS inhibition enhances viral replication and OXPHOS restoration restricts SARS-CoV-2 replication in A549-ACE2 cells.

### OXPHOS regulates the number and location of SARS-CoV-2 replication organelles

**Fig. 3.**
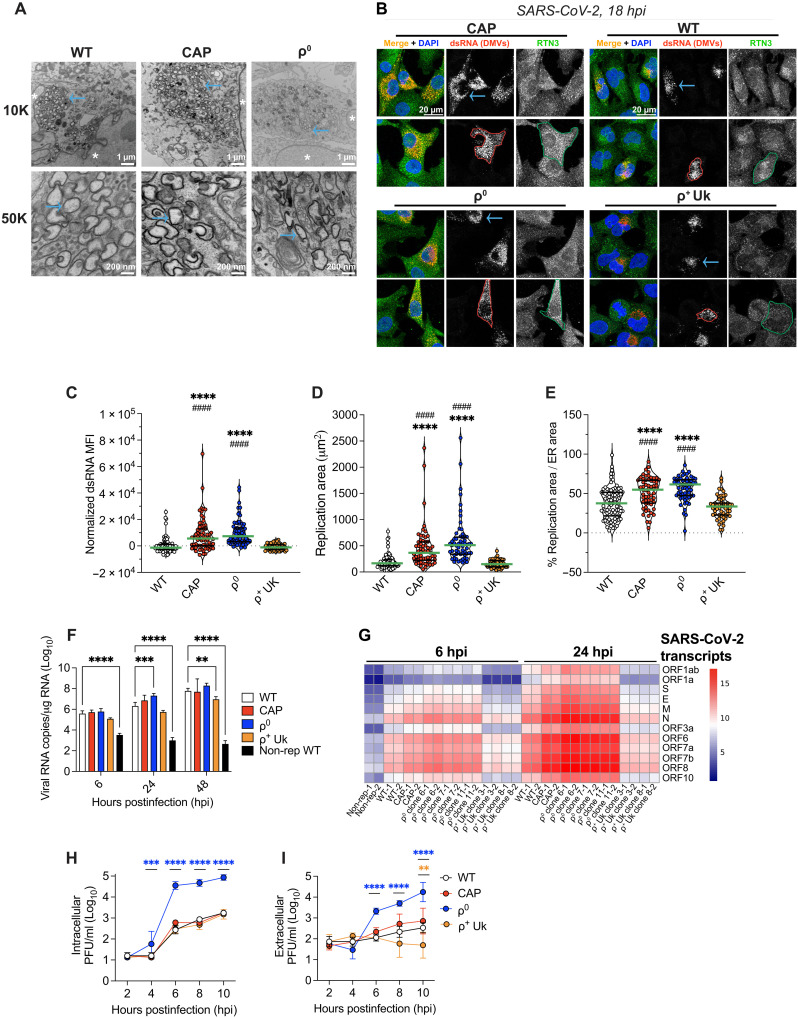
OXPHOS regulates the intracellular landscape of SARS-CoV-2 virus production. (**A**) Viral DMVs are visualized across SARS-CoV-2 MOI = 2.0 infected cells at 24 hpi by TEM where the nucleus is denoted by a white asterisk (*). Two-well replicates, *n* = 1. (**B** to **E**) SARS-CoV-2–infected cells (MOI = 10) were fixed at 18 hpi, stained with J2 anti-dsRNA and ER anti-reticulon 3, counterstained with Hoechst, and imaged by confocal microscopy. A total of 55 to 100+ infected cells per condition from 3+-well replicates across *n* = 2 to 3 were analyzed from maximum projections where each dot is a cell (C to E) and representative images and tracings shown (B). The MFI was normalized by the mean WT fluorescence per session (C), raw replication area (μm^2^) (D), or normalized to RTN3 area (E) plotted, where the median and quartile bars are green and black. (**F**) Intracellular SARS-CoV-2 gRNA was quantified by RT-qPCR to ORF1a/b at defined time points (MOI = 5) and calculated as copies per μg RNA. Nonreplicating (non-rep) virus was ultraviolet inactivated for 10 min. Two- to 3-well replicates, *n* = 2. (**G**) SARS-CoV-2 structural and accessory subgenomic mRNA transcripts detected by bulk RNA-seq on (F) samples and relative expression plotted in a heatmap as transformed transcripts per million. Two- to 3-well replicates, *n* = 2. (**H** and **I**) Plaque assays were performed on the cell pellets (intracellular) and supernatants (extracellular) of MOI = 2 SARS-CoV-2 WA1–infected cells. Two- to 4-well replicates, *n* = 2 of 3 shown. Means ± SD where applicable. Two-way ANOVA was performed compared to WT (F) WT and ρ^+^ Uk cells (C to E) or 2 hpi (H and I) where **P < 0.01; ***P < 0.001; ****P < 0.0001; ^###^*P* < 0.001. In (C) to (E), *: compared to WT and #: compared to ρ^+^ Uk cells. In (H) and (I), significance is color coded by comparison. Image brightness and contrast (A) or just brightness (B) adjusted simultaneously for visibility.

Because OXPHOS deficits change the mitochondrial ultrastructure and mitochondria are closely associated with remodeled ER networks during infection, we asked whether the presence of healthy mitochondria was required for DMV formation. SARS-CoV-2–infected (MOI = 2.0) ρ^0^, CAP-treated, and WT cells were examined by TEM at 24 hpi and found to have classic DMVs, perinuclear clusters of organelles surrounded by two membranes (Fig. 3A). These were not observed in mock-infected cells (Fig. 1E). However, CAP and ρ^0^ cells appeared to have larger networks of DMVs compared to WT, suggesting increased viral RNA replication at 24 hpi (Fig. 3A).

To quantify the DMV burden within individual cells at an earlier time point, we assayed the dsRNA foci in SARS-CoV-2–infected cells using fluorescence microscopy, a well-accepted proxy for coronaviral DMVs (*11*). We assessed three-dimensional DMVs from maximum projections acquired by confocal microscopy at 18 hpi or about ∼2 full viral replication cycles in WT A549-ACE2 cells (Fig. 3B). Both the CAP and ρ^0^ cells display increased dsRNA MFI widely dispersed throughout the cells indicative of increased DMV burden relative to WT and ρ^+^ Uk cybrid cells, confirmed by electron microscopy (Fig. 3, A to C). The extent (area) of viral replication was greater in ρ^0^ and CAP-treated cells relative to WT and ρ^+^ Uk cells, even when normalized to the ER area by antibody staining with the ER reticulon 3 (RTN3) protein that colocalizes (*13*) with the site of viral replication (Fig. 3, D and E). The calculated distance of DMVs from the nuclear edge was increased in ρ^0^ and CAP-treated cells (fig. S3, A and B). The observed increase in DMV parameters (mass, area, and distribution) in ρ^0^ cells was reduced in ρ^+^ Uk cybrids, similar to that of infected WT cells (Fig. 3, B to E, and fig. S3A). Hence, OXPHOS modulates SARS-CoV-2 DMV mass and distribution.

We also compared the distribution of DMVs relative to the intermediate filament protein vimentin. In SARS-CoV-2–infected A549-ACE2 cells, vimentin appears to form a cage around DMVs, and disruption of these vimentin cages reduces viral replication (*13*). Infected ρ^0^ and CAP cells showed displaced vimentin structures relative to DMVs, indicating that OXPHOS inhibition modifies the biogenesis of viral replication organelles: their mass, relative localization, and intracellular spread (fig. S3C). OXPHOS deficits thus modify the subcellular environment to favor SARS-CoV-2 replication through the increase in DMV cellular distribution, size, and burden.

### Deficits in OXPHOS accelerate infectious virus production at early time points

Because different OXPHOS conditions result in different DMV patterns and viral propagation levels as early as 18 hpi, we asked whether blocking OXPHOS would accelerate other aspects of the viral life cycle. SARS-CoV-2 replication was monitored by quantification of SARS-CoV-2 open reading frame 1a/1b (ORF1a/b) genomic RNA (gRNA) (*72*) using reverse transcriptase quantitative PCR (RT-qPCR) in cell pellets at 6, 24, and 48 hpi (MOI = 5.0) (Fig. 3F and fig. S3D). No difference in viral ORF1a/b gRNA copies was observed among CAP, ρ^0^, WT cells, and ρ^+^ Uk cybrids at 6 hpi, but at 24 hpi, ρ^0^ and CAP cells produced more ORF1a/b gRNA than WT and ρ^+^ Uk cybrids, and at 48 hours, WT cells more than Uk cybrids. Differential levels of all viral structural and accessory subgenomic mRNAs was confirmed by bulk RNA sequencing at 6 and 24 hpi (Fig. 3G). The increased levels of viral mRNAs in ρ^0^ and CAP cells was most pronounced for the structural N protein that wraps around gRNA in the virion (*6*), as well as ORF6 to ORF8, at 24 hpi. Only mild increases in viral RNAs in ρ^0^ cells were seen at 6 hpi (Fig. 3G). These findings are consistent with the expected relative abundance of SARS-CoV-2 RNAs (*72*). Thus, OXPHOS inhibition causes an increase in viral RNA at 24 hpi, consistent with increased viral load by plaque assay at 18 and 24 hpi and increased DMVs at 18 hpi (Fig. 1, F and G).

To further refine the differential replication kinetics of SARS-CoV-2 WA1 between ρ^0^ and CAP cells versus WT and ρ^+^ Uk cybrid cells, we assayed infectious virus contained or released from A549-ACE2 cells before a full viral life cycle had transpired (8 to 10 hpi) (*73*). Intracellular versus extracellular virus was quantified by plaque assay at 2, 4, 6, 8, and 10 hpi. Cells were washed to remove nonabsorbed virus before adding viral refeed media, as well as before cell pellet collection. The intracellular virus was released from scraped pellets by three consecutive freeze-thaws and centrifugation to clarify the supernatant. The intracellular and extracellular virus was quantified by plaque assay of the lysed cell supernatant and the culture media by plaque assay. At 2 hpi, the virions are internalized, disassembled, and viral replication initiated (the eclipse period). Hence, at this time, no virus can be detected (*74*–*76*).

By 4 hpi, intracellular SARS-CoV-2 PFU was detectable by plaque assay in ρ^0^ cells but not in WT, CAP, or ρ^+^ Uk cybrid cells until 6 hpi (Fig. 3H and fig. S3E). Furthermore, ρ^0^ cells released virus at 6 hpi, whereas WT, CAP, and ρ^+^ Uk cells did not start releasing virus until 8 hpi (Fig. 3I and fig. S3F). dsRNA staining within DMVs, a secondary measure of early viral kinetics, revealed that ρ^0^ cells harbored extensive DMV networks at 6 hpi, whereas DMVs were not observed extensively in WT cells until 10 hpi (fig. S4, A and B). Consequently, ρ^0^ cells exhibited a 100-fold increase in intracellular plus extracellular virus over WT and ρ^+^ Uk cybrid cells at 10 hpi (Fig. 3, H and I, and fig. S3, E and F), and ρ^0^ cells harbored increased DMV mass and area at 10 hpi over WT cells (fig. S4, C and D). The DMV area also expanded faster in ρ^0^ cells compared to WT cells from 10 to 18 hpi (fig. S4E). Overall, this suggests that ρ^0^ cells have increased DMVs and generate and release virus (PFU) as early as 6 hpi, before completion of a full viral life cycle in WT cells.

Thus, OXPHOS inhibition creates a SARS-CoV-2 replication advantage that persists within one and across multiple viral replication cycles. Severe OXPHOS deficits (ρ^0^ cells) accelerate SARS-CoV-2 virus detection as early as 4 and 6 hpi and DMV burden and expansion by at least 10 hpi. Restoration of OXPHOS function in ρ^+^ Uk cybrids delays the kinetics of virus production and DMV landscape to that seen in WT A549-ACE2 cells, as summarized in Table 1. A similar early difference in DMV appearance was seen for HCoV-229E–infected ρ^0^ cells at 18 hpi (fig. S4F). This suggests that accelerated virus production as well as the increase in mass and area of viral replication organelles in OXPHOS-deficient cells may extend to other coronaviruses.

**Table 1.** OXPHOS function and SARS-CoV-2 replication. A summary of how mitochondrial OXPHOS activity regulates SARS-CoV-2 replication. ↑: increased viral replication/earlier detection; ↓: decreased viral replication/later detection; *: compared to ρ^0^. WT, wild type; CAP, chloramphenicol; mtDNA, mitochondrial DNA; Mito, mitochondria; ETC, electron transport chain; OXPHOS, oxidative phosphorylation; DMVs, double membrane vesicles.

Condition	Source of OXPHOS deficit	mtDNA	Mito translation	ETC activity	Source of ATP	Effect on SARS-CoV-2 virus production	Effect on first virus detection	Effect on DMVs
WT	–	Potentially pathologic mtDNA variants	–	Baseline (Figs. 1 and 2 and fig. S2)	OXPHOS and glycolysis	WT baseline	WT baseline	WT baseline
CAP	Block in mitochondrial translation	–	Blocked (Fig. 1)	None (fig. S1)	Glycolysis	5–10X increase ↑	Earlier than WT ↑	Increased ↑
Acute CAP	Partial block in mitochondrial translation	–	Partially blocked (Fig. 1)	60% (fig. S1)	OXPHOS and glycolysis	5–10X increase ↑	–	–
Complex I inhibitor	ETC complex I activity blocked	–	–	Complex II (fig. S2)	OXPHOS and glycolysis	5–10X increase ↑	–	–
Complex III inhibitor	ETC complex III activity blocked	–	–	None (Fig. 1 and fig. S2)	Glycolysis	5–10X increase ↑	–	–
Complex V inhibitor	ETC complex V blocked	–	–	Decrease (Fig. 1 and fig. S2)	Glycolysis	5–10X increase ↑	–	–
ρ^0^ cells	mtDNA depletion	Depleted	Presumed absent (Fig. 1)	None (Fig. 1)	Glycolysis	10–100X increase ↑	Earlier than WT ↑	Increased ↑
ρ+ Uk cybrids	–	Replenished (Uk)	Normal (Fig. 2)	Comparable to WT	OXPHOS and glycolysis	*5–100X decrease ↓, less than WT	Later than WT ↓	Same as WT

### Mitochondrial dysfunction does not impair host innate immune defenses

**Fig. 4.**
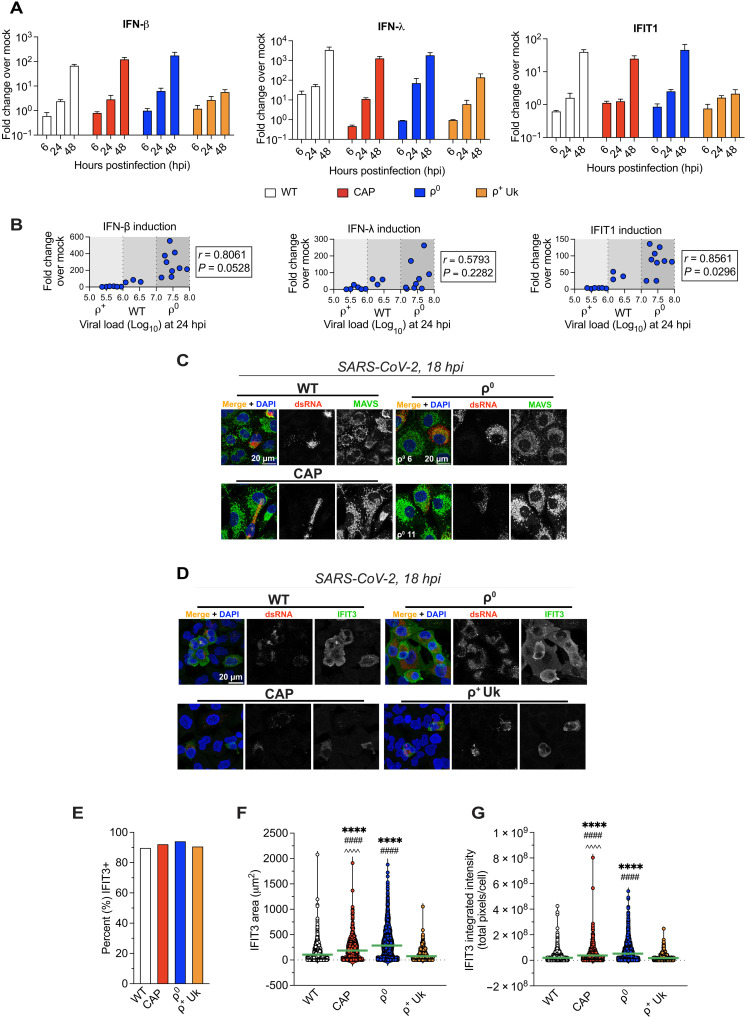
The IFN response to infection is not blunted with OXPHOS inhibition. (**A** and **B**) Cells were infected with SARS-CoV-2 (MOI = 5). (A) Intracellular RT-qPCR was performed for IFN transcripts versus *HPRT1* graphed as fold change (2^−ΔΔCT^) over matched mock-infected cells, and statistics was performed by (B) Pearson’s coefficient analysis between viral gRNA copies at 24 hpi (*x* axis, from Fig. 3F) and IFN induction at 48 hpi (*y* axis). Averaged technical duplicates, 2- to 3-well replicates, representative of *n* = 2. (**C** and **D**) SARS-CoV-2–infected cells (MOI = 10) were fixed at 18 hpi and stained and imaged by confocal microscopy as maximum projections where images are representative of *n* = 2. Staining performed with J2 anti-dsRNA and (C) anti-MAVS or (D) anti-IFIT3 and counterstained with Hoechst. Representative of 2- to 3-well replicates and *n* = 2. (**E** to **G**) Cells from (D) were imaged by an ImageXpress Hi.AT high-content imager, and percent infected cells expressing IFIT3 (E), IFIT3 area (F), and IFIT3 MFI per cell (G) were calculated, *n* = 1. Means ± SD where applicable. Where statistics are shown, a two-way ANOVA was performed across all samples where ****P < 0.0001; ^####^*P* < 0.0001; ^^^^*P* < 0.0001. In (E) to (G), *: compared to WT, #: compared to ρ^+^ Uk cells, and ^: compared to ρ^0^ cells. See also figs. S5 to S7. Image brightness adjusted simultaneously for visibility.

Because the release of mtDNA and mtRNA into the cytosol is known to activate cGAS-STING, the NLRP3 inflammasome, and TLR-9, one possible reason that OXPHOS inhibition could stimulate SARS-CoV-2 replication is the absence of innate immune activation (fig. S5A). Downstream cytokines including type I and III IFNs are known to restrict SARS-CoV-2 replication (*50*). To determine whether mitochondrial dysfunction impairs mitochondria-mediated immunity during SARS-CoV-2 infection, we assayed IFN-β and IFN-λ, and IFN-induced protein with tetratricopeptide repeats 1 (IFIT1, an ISG) transcripts at 6, 24, and 48 hpi (MOI = 5). We found that ρ^0^ cells still generate inflammatory cytokines, and ρ^0^ and CAP cells launch an IFN transcriptional program starting with phosphorylation of signal transducer and activator of transcription 1 (p-STAT1) in response to SARS-CoV-2 infection (*77*) at least as strong as that of WT cells and ρ^+^ Uk cybrids (Fig. 4, A and B, and figs. S5B and S6A).

Strong induction of the IFN inflammatory response in the ρ^0^ A549-ACE2 cells was unexpected because A549 cells do not express STING (*78*) and lack robust endogenous NLRP3 inflammasome capacity (*53*). In cells with an active NLRP3 inflammasome, apoptosis-associated speck-like protein (ASC) typically forms one 1- to 2-μm oligomer or “speck” (*79*). We induced the NLRP3 inflammasome in ρ^0^, WT, and ρ^+^ Uk cybrid cells with priming and activation signals lipopolysaccharide (LPS) and nigericin and quantified ASC speck formation by confocal microscopy. All derivatives showed an equally minimal response to these inflammasome activators, as seen by a limited number of specks across all tested conditions (fig. S6, B and C).

Because ρ^0^ cells lack mtDNA, the increased ρ^0^ cell IFN response cannot be due to mtDNA or mtRNA signaling. Therefore, we considered whether the robust IFN response could be due to MDA-5 signaling through outer mitochondrial protein MAVS in response to SARS-CoV-2 viral RNA. MAVS expression or localization could be disrupted by the altered mitochondrial networks of ρ^0^ and CAP cells. However, we found that all conditions expressed MAVS with mitochondrial patterning (Fig. 4C and fig. S7, A and B). We also found that the IFIT3 protein, another ISG and a MAVS adaptor that amplifies the IFN response, was up-regulated in nearly all infected cell lines regardless of OXPHOS function. The cytoplasmic expression was stronger and broader in ρ^0^ and CAP cells than in WT and ρ^+^ Uk cells (Fig. 4, D to G). Stronger IFIT3 expression (MFI and area) was confirmed even in mock-infected (uninfected) ρ^0^ cells (fig. S7, C to E). Last, we asked whether the IFN response was specific to viral infection. We found that ρ^0^ cells also respond robustly to immunostimulatory polyinosinic:polycytidylic acid [poly(I:C)] RNA, showing that the strong immune response is not limited to actively replicating virus (fig. S6D).

To confirm the relationship between IFN response and SARS-CoV-2 propagation, we conducted a Pearson’s correlation analysis on IFN transcript induction as a function of cellular viral gRNA ORF1a/b levels at 24 hpi. IFN induction for all cells was directly proportional to SARS-CoV-2 replication and inversely proportional to OXPHOS function; ρ^0^ and CAP cells manifested increased viral replication as well as increased IFN production (*IFN-β*: *r* = 0.8061, *P* = 0.0528; *IFIT1*: *r* = 0.8561, *P* = 0.0296) relative to WT cells, as well as ρ^+^ Uk cybrids, which showed the least IFN response (Fig. 4, A and B). Hence, increased SARS-CoV-2 replication in OXPHOS-deficient cells is likely not due to a blunted innate immune response (Fig. 4 and figs. S5 to S7).

To confirm that the ρ^0^ and CAP cells were not defective in baseline innate immune function, we performed bulk RNA-seq on mock-treated WT cells, three ρ^0^ clones, CAP cells, and the two ρ^+^ Uk cybrids clones. Using unsupervised Gene Ontology (GO) pathway analysis of alterations in ρ^0^, CAP, WT, and ρ^+^ Uk cell transcripts, we found reduced gene expression related to cell cycle progression in ρ^0^ and CAP cells consistent with their reduced cell division rate (fig. S8A and table S2). Using gene set enrichment analysis of hallmark gene lists, we found major changes in differentially expressed genes (DEGs) of immune function in the ρ^0^ and CAP cells versus WT and ρ^+^ Uk cells with the greatest log_2_ fold change for genes associated with general inflammation, TNFα signaling, and IFN-α activation (fig. S8B). This confirmed that ρ^0^ and CAP cells up-regulate inflammatory genes—both IFN and NF-κB—even in the absence of induction by SARS-CoV-2 infection or poly(I:C). Thus, the increased viral replication in ρ^0^ and CAP cells is not due to a blunted innate immune response, which is even more unexpected given the heightened baseline antiviral state.

### Glycolysis reprogramming positively correlates with SARS-CoV-2 replicative capacity

**Fig. 5.**
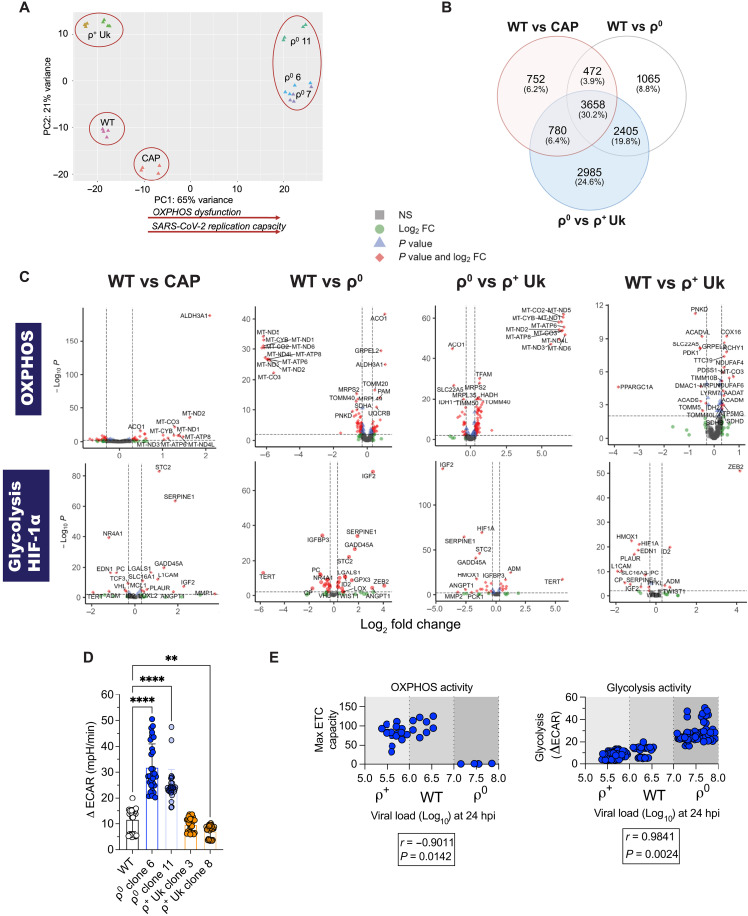
OXPHOS inhibition is linked to bioenergetic remodeling and up-regulation of glycolysis. (**A** to **C**) Bulk RNA-seq of uninfected cells highlights host bioenergetics as a correlating variable with viral replication. Dataset represents 2-well replicates, two (ρ^+^ Uk cybrids) or three (ρ^0^ cells) clones, *n* = 2 and batch corrected, and only genes with *P*-adjusted values ≤ 0.05 are represented in downstream analysis. (A) PCA plot, (B) a Venn diagram of DEGs where the gene percentage is in parentheses was created in BioRender [A. Chilkatowsky (2026) https://BioRender.com/g3pwkoe], and (C) volcano plots of DEGs across OXPHOS or HIF-1α/glycolysis gene lists are shown. FC, fold change. (**D**) Seahorse analysis by ECAR is a proxy for glycolysis activity. DAPI+ nuclei-corrected ECAR post–glycolysis stress test, where ΔECAR, upon 10 mM glucose addition, is plotted. Ten-well replicates, *n* = 3. (**E**) Pearson’s coefficient analysis performed where OXPHOS activity (max ETC capacity from Fig. 2C) and glycolysis activity [ΔECAR from (D)] are plotted on the *y* axis. All independent experiments shown. Means ± SD where applicable. Where statistics are shown, a two-way ANOVA was performed compared to WT where **P < 0.01; ***P < 0.001; ****P < 0.0001. See also fig. S8 and tables S2 to S4.

Having established that the ρ^0^ and CAP cells are not defective in innate immune gene expression (*51*–*58*), we investigated characteristics of the metabolic switch from substrate oxidation to glycolysis in the ρ^0^ and CAP cell states of mitochondrial stress. OXPHOS degrades metabolites to O_2_ and H_2_O, whereas glycolysis preserves carbon backbones for the biosynthesis of nucleic acids, amino acids, and lipids required for virion production; SARS-CoV-2 prefers the latter (*25*). Principal components analysis (PCA) revealed that 65% of transcriptome variance was explained by differences in OXPHOS function among the ρ^0^, CAP, WT, and ρ^+^ Uk cells (Fig. 5A). The ρ^+^ Uk cybrid clones clustered to the extreme top left of the PCA plot, the ρ^0^ clones clustered to the extreme top right, and WT and CAP cells were arrayed between these two extremes, with WT cells located in the bottom-left corner and CAP cells in the bottom center. Hence, most of the physiological differences between these cell lines appear determined by bioenergetic status.

Venn diagrams of DEGs between WT versus CAP, WT versus ρ^0^, and ρ^0^ versus ρ^+^ Uk cybrids revealed that 30.2% (3658) of the DEG changes were shared by all three comparisons, confirming that differential OXPHOS function is the predominant determinant of the physiological status of these cell lines (Fig. 5B). Exploring these comparisons for DEGs from curated bioenergetic gene sets revealed that 21% (*38*) of OXPHOS genes and 37.2% (*16*) of glycolysis/HIF-1α genes were shared across all comparisons (fig. S8C). As expected, the ρ^0^ cells lacked mtDNA transcripts (fig. S8D).

Volcano plots further exemplified the importance of the bioenergetic switch from the oxidative metabolism of WT and ρ^+^ Uk cells to the glycolytic metabolism of ρ^0^ and CAP cells (Fig. 5C). As expected, the absence of mtDNA in ρ^0^ cells was associated with reduced mtDNA transcripts. This was associated with the induction of nDNA-coded mitochondrial transcripts such as aconitase (ACO1), mitochondrial import protein translocase of outer mitochondrial membrane 20 (TOMM20), and complex III [ubiquinol–cytochrome c reductase binding protein (UQCRB)] genes (Fig. 5C, “OXPHOS” and “WT vs ρ^0^”). In CAP cells, the mtDNA transcripts were up-regulated relative to WT consistent with CAP inhibition of mitochondrial translation being compensated by the induction of mtDNA transcription (Fig. 5C, “OXPHOS” and “WT vs CAP”, and fig. S8D). For ρ^+^ Uk relative to ρ^0^ cells, mtDNA transcripts were increased in association with the mtDNA transcription factor TFAM as well as other nDNA mitochondrial transcripts such as Tomm40 (Fig. 5C, “OXPHOS” and “ρ^0^ vs ρ^+^ Uk”). The OXPHOS-deficient ρ^0^ cells and CAP cells, relative to WT and ρ^+^ UK cells, up-regulated glycolytic genes including HIF-1α, which induces transcription of glycolytic genes, and heme oxygenase-1 (HMOX1), important in hypoxia and antioxidant defenses (*80*) (Fig. 5C, “glycolysis/HIF-1α” and “ρ^0^ vs ρ^+^ Uk”). The ρ^0^ and CAP cells compared to WT cells and ρ^+^ UK cybrids also induced stress response genes. These included insulin-like growth factor 2 (IGF2) (*81*), stanniocalcin 2 (STC2) (*82*), α-1 antitrypsin (SERPENE1) (*83*), and growth arrest and DNA damage–inducible protein 45alpha (GADD45A) (*84*) (Fig. 5C, “glycolysis/HIF-1α” and “WT vs ρ^0^”).

Although the bioenergetic differences between ρ^0^ and CAP versus WT and ρ^+^ Uk cells were expected, the transcriptional differences between WT A549-ACE2 cells with their endogenous mtDNA versus the ρ^+^ Uk cybrids with A549-ACE2 nDNA but ρ^+^ Uk mtDNA was unexpected. WT cells exhibited a notable up-regulation of peroxisome proliferator-activated receptor gamma coactivator 1-alpha (PGC-1α), nearly a log_2_ fold change of 4 (Fig. 5C, “OXPHOS”). PGC-1α is the coactivator for global transcription induction of nDNA-coded mitochondrial genes, and its compensatory up-regulation in cells with mitochondrial dysfunction can improve OXPHOS function (*85*). The induction of PGC-1α was associated with extensive alterations in an array of mitochondrial genes, indicating that WT cells, with their endogenous mtDNA, could have a partial mitochondrial defect relative to ρ^+^ UK cybrids and thus be more glycolytic.

To validate the finding that OXPHOS inhibition in the ρ^0^ cells shifted metabolism toward glycolysis, we compared the oxidative and glycolytic metabolism of ρ^0^, WT, and ρ^+^ UK cells by the Seahorse glycolysis stress test. This revealed a two- to threefold increase in the glycolytic surrogate, extracellular acidification rate (ECAR), when ρ^0^ cells metabolized 10 mM glucose compared to WT cells and ρ^+^ Uk cybrids (Fig. 5D and fig. S9A). WT cells also showed slightly increased glycolysis compared to ρ^+^ Uk cybrids, with ρ^+^ Uk clone 8 showing the greatest reduction. ECAR levels correlated directly and highly with capacity for SARS-CoV-2 replication (Pearson’s correlation *r* = 0.98, *P* = 0.002) (Fig. 5E, “Glycolysis”), whereas OCR levels (OXPHOS) were inversely proportional to viral replication (*r* = 0.90, *P* = 0.0142) (Fig. 5E, “OXPHOS”). Last, we examined protein-level changes of two factors important to glycolysis: pyruvate kinase M2 (PKM2), a crucial glycolytic enzyme catalyzing the final, rate-limiting step, and the GLUT-3 glucose transporter, both downstream of HIF-1α and causally linked to promoting inflammation (*86*, *87*). GLUT-3, but not PKM2, was equally increased in ρ^0^ cells across mock an 24 and 48 hpi conditions when compared to cells harboring mtDNA (fig. S9B).

Because A549-ACE2 WT and cybrid cells differ only in the endogenous mtDNA versus the replaced ρ^+^ Uk mtDNA, we asked whether the endogenous mtDNA might harbor mtDNA variants that could partially impair OXPHOS. Both of the mtDNAs are common European mtDNA lineages (haplogroups); the parental A549-ACE2 being H4a, whereas cybrid mtDNA is Uk1a (table S1, tab 1). However, closer analysis of individual nucleotide changes revealed that the parental WT A549-ACE2 mtDNA harbors two novel homoplasmic (100% mutant) and three heteroplasmic variants (≥20% mutant) rarely if ever seen in the ∼300,000 mtDNA sequences currently documented (table S1, tabs 2 to 4). The homoplasmic variants were *MT-RNR2* m.3097T>C in the 16*S* rRNA gene, which is 100% conserved across an array of multicellular animals and could affect mitochondrial protein synthesis, and *MT-ND1* m.3696C>A, a transversion mutation—rare for mtDNA polymorphisms—changing amino acid 30 from an isoleucine to a methionine (Ile^30^ to Met) where the isoleucine is 84% conserved across diverse animals and should affect the complex I function. The heteroplasmic mutations include *MT-ND1* m.3814G>T nonsense mutation (Glu^170^ to STOP) at 20% heteroplasmy, *tRNA^Ala^* m.5611C>A that is 93% conserved at 71% heteroplasmy, and *MT-CO2* m.7598G>A missense mutation (Ala^5^ to THr) that is 18% conserved at 21% heteroplasmy. These novel mtDNA mutations would impair OXPHOS and may reflect the tumor origin of the A549 cells because solid tumors often manifest aerobic glycolysis (*88*, *89*). By contrast, the Uk mtDNA had only one novel variant, an *MT-ND1* m.3931T>C missense (S209P), which alters a conserved amino acid present at 29% heteroplasmy (table S1, tabs 3 and 4). By shifting the A549-ACE2 metabolism toward glycolysis, the native A549 mtDNA mutations could explain why the WT parental cell line clusters away from the ρ^+^ Uk cybrids transcriptionally and shows a fourfold increase in PGC-1α expression as well as exhibits increased glycolysis despite sharing the same nDNA. These observations, in turn, could explain how the WT parental cell line sustains more robust SARS-CoV-2 replication than the ρ^+^ Uk cybrids including increased early and late viral titers (Fig. 3F and fig. S3D) and reaching maximum percent infection ∼18 hours before Uk clone 3 (Fig. 2G).

Overall, the transcriptomic data show that cells with OXPHOS deficits (ρ^0^ and CAP cells) exhibit up-regulated glycolytic pathways along with a signature of increased HIF-1α/hypoxia and mitochondrial stress, all of which increase glycolytic programming (*80*, *84*). Transcriptomics and the Seahorse assay also revealed that WT cells were more glycolytic than the derived ρ^+^ Uk cybrids, implying that mtDNA mutations can change the metabolic balance between OXPHOS and glycolysis in the cell. The aggregate data provide a coherent indication that impaired OXPHOS and enhanced glycolysis favor SARS-CoV-2 replication.

### Increased glycolysis is required to enhance SARS-CoV-2 replication when OXPHOS is inhibited

**Fig. 6.**
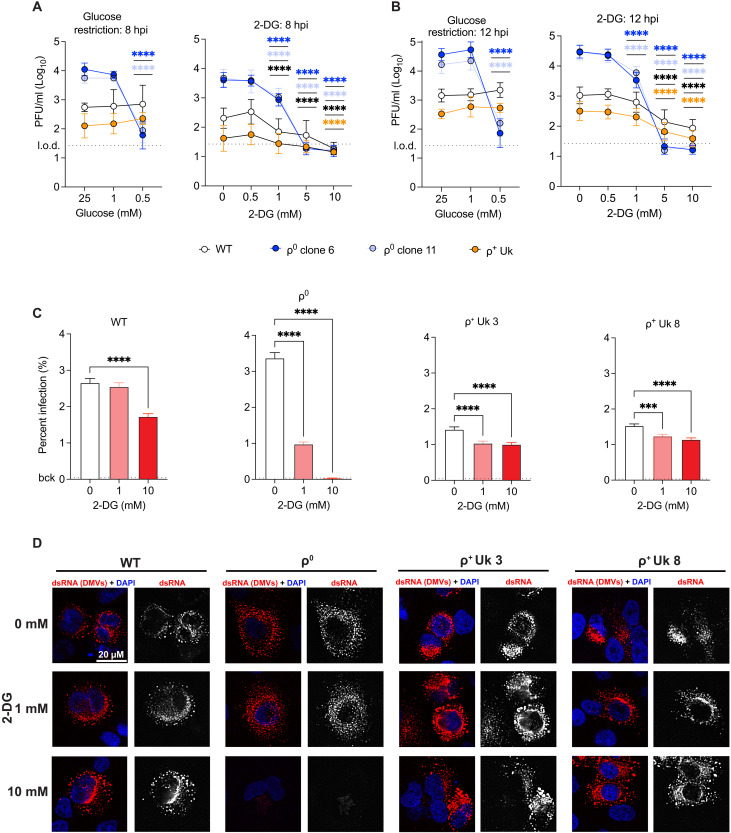
OXPHOS regulates SARS-CoV-2 replication via glycolytic capacity. (**A** and **B**) Cells were infected with SARS-CoV-2 WA1 (MOI = 2), glucose depleted or glycolysis inhibited by competitive 2-DG treatment in the viral refeed media and viral replication assessed by supernatant plaque assay at 8 hpi (A) and 12 hpi (B). Means ± SD. Two- to 4-well replicates, *n* = 3. (**C** and **D**) Cells were infected with SARS-CoV-2 WA1 (MOI = 10) and treated with 2-DG in the viral refeed media, fixed, and imaged at 12 hpi by the ImageXpress Micro Confocal as maximum projections. Staining was performed with J2 anti-dsRNA and counterstained with Hoechst and (C) percent infection calculated with means ± SEM shown. The dotted line represents the background (bck) or percent dsRNA calls in mock-treated and 0 mM 2-DG–treated wells of each cell type. An ordinary one-way ANOVA was performed compared to 0 mM 2-DG (control) and (D) shows representative images. Three replicate wells, 49 sites per well, *n* = 3 plates. ANOVA significance is as follows: ***P < 0.001 and ****P < 0.0001 and are color coded by comparison in (A) and (B). Image brightness adjusted simultaneously for visibility. See also figs. S9 and S10.

To demonstrate a causal relationship between dependence on glycolysis and SARS-CoV-2 replication, ρ^0^, WT, and ρ^+^ Uk cybrid cells were cultured in varying concentrations of glucose and 2-deoxyglucose (2-DG), a competitive inhibitor of hexokinase and thus glycolysis, and tested for effects on virus production. Four different glucose levels (0.5, 1, 5, and 10 mM) and 2-DG inhibitor levels (0.5, 1, 5, and 10 mM) were selected for minimal toxicity on ρ^0^ cells (fig. S9C) because ρ^0^ cells detach in glucose-free media by 12 hours (fig. S9E). Base media for 2-DG studies contained 25 mM glucose (equivalent to 4.5 g/liter glucose) and dialyzed fetal bovine serum (FBS) was used for the glucose restriction studies. Because glucose and 2-DG concentrations were applied to the viral refeed media, the time during glycolysis modulation coincided precisely with time postinfection. Cells were infected with SARS-CoV-2 (MOI = 2.0) and viral replication quantified in the supernatant by plaque assay at 8 hpi (when WT cells first release the virus) and 12 hpi (Fig. 6, A and B). At 25 mM glucose, ρ^0^ cells produced 10- to 50-fold more virus than WT cells and ρ^+^ Uk cybrids, and WT cells generated ∼5-fold more virus than ρ^+^ Uk cybrids (Fig. 6B, all panels). ρ^0^ cells showed sustained SARS-CoV-2 replication at 25 and 1 mM glucose at both 8 and 12 hpi, but virus production in ρ^0^ cells was reduced 100- to 500-fold at 0.5 mM glucose at 8 and 12 hpi, respectively (Fig. 6, A and B). There was no evidence of cell death, quantified by the cellular release of lactate dehydrogenase (LDH), regardless of infection state or treatment condition (fig. S9, C and D). Hence, ρ^0^ cell death was not the cause of impaired viral replication. Meanwhile, glucose restriction in the assayed range did not affect viral replication in WT cells or ρ^+^ Uk cybrids. Hence, SARS-CoV-2 replication in A549-ACE2 cells with functional OXPHOS (WT cells and cybrids) is unaffected by glucose levels at or above 0.5 mM (1/50th), but when glycolysis is diminished to the same extent in ρ^0^ cells, viral replication is nearly lost. This occurs despite the fact that glucose treatment causes increased GLUT-3 expression in ρ^0^ cells compared to WT cells and ρ^+^ Uk cybrids (fig. S9F), demonstrating the importance of glycolytic flux in this system.

ρ^0^ viral replication was also more susceptible to 2-DG glycolysis inhibition than WT and ρ^+^ Uk cybrids cells at 12 hpi (Fig. 6, A and B). ρ^0^, WT, and ρ^+^ Uk cybrid cells manifested a partial reduction in viral titers at 1 mM of 2-DG relative to untreated controls at 8 hpi, although the effect was greater for ρ^0^ cells compared to WT cells at 12 hpi. 2-DG (5 and 10 mM) was completely inhibitory to SARS-CoV-2 replication at 12 hpi in ρ^0^ cells, whereas virus production in WT and ρ^+^ Uk cells was only partially affected. To investigate which parameter of viral replication was restricted upon 2-DG treatment, we infected ρ^0^, WT, and ρ^+^ Uk cybrid cells and treated with 0, 1, or 10 mM 2-DG, this time staining for dsRNA as a proxy for viral replication centers or DMVs at 12 hpi (Fig. 6, C and D). We found that all cell types showed a dose-dependent decrease in percent infection (Fig. 6, C and D), with complete suppression in ρ^0^ cells treated with 10 mM 2-DG, consistent with virus production (Fig. 6, A and B). A 1 and 10 mM 2-DG treatment seemed to increase the area of viral replication in WT and ρ^+^ Uk cybrids cells (Fig. 6D and fig. S10A), which was not met with a concomitant rise in dsRNA MFI (fig. S10B). In summary, although inhibition of SARS-CoV-2 replication was proportional to 2-DG concentration for all cell lines, ρ^0^ cells were more sensitive at the concentrations used (1 to 10 mM 2-DG) and these cells further showed a threshold effect on SARS-CoV-2 replication in response to glucose restriction. Overall, glycolysis activity is linked to changes in virus production, percent infection, and the intracellular distribution of viral replication centers (Fig. 6).

In summary, ρ^0^ cells with reduced OXPHOS up-regulate glycolysis, create a favorable environment for SARS-CoV-2 replication. Moreover, the balanced OXPHOS-to-glycolysis ratio in WT and ρ^+^ Uk cells limits viral replication. Thus, glycolytic flux at the expense of OXPHOS energy production appears to be the major factor in driving SARS-CoV-2 replication. Hence, OXPHOS limits SARS-CoV-2 replication by reducing glycolytic capacity.

## DISCUSSION

We sought to clarify the role of OXPHOS in SARS-CoV-2 replication. It has been suggested that SARS-CoV-2 proteins may be translated on mitochondrial ribosomes and that inhibiting mitochondrial protein synthesis should decrease viral replication, based in part on early reports of SARS-CoV-2 RNA localizing to the mitochondrial matrix (*24*, *38*–*42*, *44*). However, we found that ablating mitochondrial biogenesis by both removing A549-ACE2 mtDNA (ρ^0^ cells) or inhibiting mitochondrial protein synthesis with CAP increases rather than decreases SARS-CoV-2 replication. A proviral environment is also created by individual OXPHOS complex inhibitors applied in the viral refeed media, but the greatest effect is achieved by the complete dissolution of OXPHOS complexes I, III, IV, and V by mtDNA depletion in ρ^0^ cells. Enhanced viral replication is not the result of a blocked mitochondrial innate immune response because ρ^0^ and CAP cells have up-regulated innate immune function yet support the most viral replication. Our study uniquely links the OXPHOS/glycolysis metabolic switch to SARS-CoV-2 viral replication organelle (DMV) burden and distribution in infected cells as well as infectious viral particle production. The SARS-CoV-2 life cycle is 8 hours in WT cells and may be as short as 6 hours in ρ^0^ cells, which could underpin the compounded 10- to 100-fold increase in supernatant virus throughout infection. Thus, OXPHOS inhibition results in increased glycolytic capacity, which favors SARS-CoV-2 replication.

In cells with functional OXPHOS, SARS-CoV-2 inhibits mitochondrial function by blocking the transcription of nDNA-coded and mtDNA-coded OXPHOS genes (*21*), causing more extreme OXPHOS suppression than that by other respiratory viruses (*22*). Until now, it was not known whether suppressed mitochondrial OXPHOS was a by-product of or a critical player in promoting SARS-CoV-2 replication; our findings suggest the latter. The resulting OXPHOS inhibition during SARS-CoV-2 infection of cells with a previously functional ETC increases mROS production and stabilizes HIF-1α to induce glycolytic gene expression (*21*–*24*, *90*, *91*), and this shifts cellular metabolism from OXPHOS toward glycolysis to maximize the available substrates for viral biogenesis (*25*). We find that preexisting reductions in host OXPHOS augment this metabolic shift to glycolysis—as demonstrated by a baseline up-regulation in glycolytic genes, proteins, and activity (Fig. 5 and figs. S8 and S9, A and B)—explaining the enhanced SARS-CoV-2 replication in ρ^0^ and CAP-treated cells. Confirmation that a metabolic shift from OXPHOS to glycolysis caused the proviral environment is shown by the abrupt inhibition of SARS-CoV-2 replication in ρ^0^ cells at low glucose and the graded inhibition by the 2-DG inhibitor of glycolysis (Fig. 6, A and B). SARS-CoV-2 replication in ρ^0^ cells is more sensitive to glucose restriction than in WT cells and ρ^+^ UK cybrid cells, demonstrating that the shift in glucose utilization and not glucose availability determines the amount of virus produced.

Coronavirus DMVs house viral dsRNA, have long been linked to viral RNA synthesis, and have been proposed to enable the critical function of immune evasion (*6*, *11*–*13*). Although SARS-CoV-2 inhibits OXPHOS and induces glycolysis in virally infected cells (*21*, *22*), DMV formation has not been linked specifically to this metabolic rewiring. We demonstrate that the intracellular distribution of DMVs, or dsRNA foci, is regulated both by changes in OXPHOS and glycolysis. Cells lacking OXPHOS activity and exhibiting a concomitant rise in glycolysis, ρ^0^ cells, and CAP-treated cells show increased DMV mass and area upon SARS-CoV-2 infection when compared to cells with intact OXPHOS capacity, WT, and ρ^+^ UK cybrid cells (Fig. 3, A to E, and figs. S3, A to C, and S4, A to E). Conversely, inhibiting glycolysis with 10 mM 2-DG enhanced the DMV area but not the mass in WT and ρ^+^ UK cybrid cells (Fig. 6D and fig. S10, A and B), linking an expansion in viral replication area in glycolysis-restricted cells to both reduced viral titers and percent infection (Fig. 6, A to C). This expansion in DMV area upon glycolysis inhibition was difficult to assess in ρ^0^ cells due to their extreme sensitivity to 10 mM 2-DG treatment (Fig. 6, A to D). The metabolic regulation of SARS-CoV-2 DMV formation, the impact of OXPHOS and glycolysis activity on DMV function, and the possibility of leveraging this relationship to develop antivirals warrant further investigation.

Differential reliance on OXPHOS versus glycolysis may also explain the greater SARS-CoV-2 replication in A549-ACE2 versus HEK293T cells when treated with OXPHOS complex inhibitors (Fig. 1, I and J, and fig. S1, I to K). A549 cells are from a lung adenocarcinoma that was transformed by the Kirsten Ras oncogene (KRAS) G12S gain-of-function missense mutation (*92*), a mutation known to inhibit mitochondrial function biasing energetics away from OXPHOS toward glycolysis (*93*, *94*). HEK293T cells were derived from a fetal kidney culture transformed with adenovirus 5 and are more biased toward OXPHOS (*95*). Therefore, the more glycolytic A549-ACE2 cells may provide a more favorable metabolic environment for SARS-CoV-2 replication upon OXPHOS inhibition than do the more oxidative HEK293T cells. Thus, a necessary limitation of this system is the requirement of existing oncologic metabolic rewiring to model extreme OXPHOS manipulations.

The cell biological processes that transpose cellular bioenergetics into increased SARS-CoV-2 replication were also consistent with changes to vimentin intermediate filament structures as they relate to viral DMVs. Vimentin rearrangements support the viral replication of several coronaviruses, including SARS-CoV-2 (*13*, *96*, *97*). When the vimentin network of A549-ACE2 cells is disrupted with withaferin A, SARS-CoV-2 DMVs are more dispersed, viral replication is inhibited and the amount of infectious virus released is greatly reduced (*13*). Other studies also link mitochondrial dysfunction to collapsed vimentin filaments (*70*, *71*), mirroring the collapsed vimentin networks we observed in infected and uninfected ρ^0^ and CAP cells (figs. S3C and S4, A and B). This is consistent with the cytoskeletal signatures in ρ^0^ and CAP cells compared to WT and ρ^+^ cybrid cells by GO analysis (fig. S8A and table S2). Applying complex IV but not complex I inhibitors to cells induces the collapsed vimentin network phenotype (*70*), indicating that not all OXPHOS dysfunction is inherently tied to vimentin remodeling and that vimentin rearrangements may not explain the increase in SARS-CoV-2 replication in complex I–inhibited A549-ACE2 or HEK293T cells (Fig. 1, I and J, and fig. S1, I to K). However, a collapsed vimentin network could still contribute to increased SARS-CoV-2 replication in ρ^0^ and CAP cells, and this variable needs to be further explored.

Our observation that OXPHOS inhibition enhances HCoV-229E replication suggests that inhibiting OXPHOS to drive glycolysis may be a general coronavirus strategy. SARS-CoV-2 and HCoV-229E share the replicase genes from two ORFs (ORF1a/b) coding for 16 nonstructural proteins; four structural genes S, E, M, and N; and functional homology of one accessory gene ORF3a for SARS-CoV-2 and ORF4 (or ORF4a/b) for HCoV-229E (*7*, *98*). SARS-CoV-2 and HCoV-229E share two viral proteins that modulate cellular metabolism, nsp4 as well as ORF3a for SARS-COV-2 or the ORF4 homolog for HCoV-229E (*99*–*102*). Because OXPHOS inhibition is linked to increased viral burden in infected cells, these viral proteins could link mitochondrial dysfunction to viral replication organelles and titers during SARS-CoV-2 and HCoV-229E infection and should be explored in future studies.

Our findings contribute to a more nuanced understanding of the combined role of OXPHOS and glycolysis in regulating SARS-CoV-2 replication and possibly disease. This is exemplified by our observation that WT A549-ACE2 cells with their endogenous mtDNA support significantly more robust and rapid SARS-CoV-2 replication than do A549-ACE2 cells whose mtDNA have been replaced by ρ^+^ Uk mtDNA. Transcriptomic analyses revealed WT cells to be more glycolytic than ρ^+^ Uk cybrids. Because mtDNA codes exclusively for OXPHOS genes, the five novel mtDNA variants we found in the endogenous A549-ACE2 cell mtDNA likely impair mitochondrial OXPHOS, indicated by the compensatory (*85*) increase in PGC-1α (Fig. 5C, “OXPHOS”). This would explain why substituting the endogenous mtDNA with ρ^+^ Uk mtDNA reduced SARS-CoV-2 replication.

Patients with more severe COVID-19 have higher viral titers (*14*–*16*), and certain mtDNA variants have been linked to increased risk of severe COVID-19, hospitalization, and death (*103*–*106*). Hence, our findings support mtDNA variation as an important factor in COVID-19 severity.

If decreasing OXPHOS increases SARS-CoV-2 viral replication, then increasing OXPHOS should impair it. This is supported by the success of metformin in treating COVID-19 in recent clinical trials. Because metformin inhibits glucose production and utilization and boosts mitochondrial biogenesis, our findings support the antiviral activity of metformin against SARS-CoV-2. Metformin is an oral antidiabetic medication that boosts OXPHOS activity via AMP-activated protein kinase (AMPK) activation of PGC-1α (*107*) and reduces the risk of mortality from COVID-19 (*108*–*110*). Metformin additionally reduces the risk of Long Covid postviral sequalae (*111*, *112*) by 60% (10.4 to 6.3%) compared to metformin-placebo–treated patients (*113*). In metformin-treated cancer cell lines, SARS-CoV-2 viral titers can be suppressed up to 99% via AMPK activation, and virus-reducing activity has also been shown in patients (*114*, *115*). Our study is consistent with metformin improving COVID-19 outcomes through boosting OXPHOS capacity, decreasing glucose utilization, and restricting SARS-CoV-2 replication.

In summary, our findings that OXPHOS restricts SARS-CoV-2 replication by limiting glycolytic capacity provide insight into correlative observations of mtDNA background affecting COVID-19 severity and causative associations between metformin treatment and decreased mortality from COVID-19. Further studies on how the metabolic balance of OXPHOS and glycolysis regulates SARS-CoV-2 replication will enhance the understanding of metabolic susceptibilities to increased viral replication and disease and how this relationship can be reversed to improve acute and Long Covid outcomes.

## MATERIALS AND METHODS

### Generation and validation of transmitochondrial cybrids

To prepare transmitochondrial cybrids (*116*–*118*), 10^6^ mtDNA-deficient A549-ACE2 ρ^0^ clone 11 cells were fused to cytoplasts of 143B(TK^−^) Uk cybrids using 100 μl of polyethylene glycol (Sigma-Aldrich, P7306). The mtDNA Uk cytoplasts were prepared by seeding 143B(TK^−^) Uk cybrids in a T75 flask and treatment 16 hours later with cytochalasin B (10 μg/ml; Sigma-Aldrich, C6762) for 15 min, followed by harvest by trypsinization and counting. Ten million treated cells were applied to a Percoll isopycnic gradient (*119*). Cells were centrifuged at 17,300*g* for 20 min on the gradient, and the Uk cytoplast band was removed from the upper third of the gradient and washed with complete Dulbecco’s modified Eagle’s medium (DMEM).

After being established for 2 to 3 days in complete RPMI medium supplemented with glucose, uridine, and pyruvate (GUP), cybrids were selected in RPMI ATCC (American Type Culture Collection) modifications supplemented with 10% of dialyzed FBS (Thermo Fisher Scientific, 26400044) with 1% penicillin/streptomycin, without uridine, plus hypoxanthine-aminopterin-thymidine (HAT) (Sigma-Aldrich, H0262-10VL). The complete absence of uridine selected against the parental mtDNA-deficient A549-ACE2 ρ^0^ clone 11 cells while permitting A549-ACE2 cells that had acquired the 143B(TK^−^) ρ^+^ Uk mtDNA to grow. HAT selected against any 143B(TK^−^) cells that had escaped enucleation. Transmitochondrial cybrid colonies were isolated at 22 days and subsequently expanded in RPMI GUP complete media. Clonal isolates were screened for those harboring the A549-ACE2 nucleus (ACE2 overexpression transgene cassette and expressed ACE2 protein), were mononuclear (by karyotyping), were mtDNA repleted (by qPCR), and contained the Uk mtDNA by sequencing. Two A549-ACE2 ρ^+^ Uk mtDNA clones, 3 and 8, were selected for subsequent experiments. A549-ACE2 ρ^+^ Uk mtDNA cybrids were grown in T75 flasks and used when 60 to 80 ± 10% confluent.

### Institutional approvals

All procedures were performed in biosafety level 3 (BSL-3) facilities at the University of Pennsylvania and National Emerging Infectious Diseases Laboratories (NEIDL) of Boston University, using biosafety protocols approved by the respective Institutional Biosafety Committees (IBC).

### Quantification and statistical analysis

The data were quantified in Prism 10.0 (GraphPad, La Jolla, CA, USA), and the appropriate statistical analysis performed using one-way or two-way analysis of variance (ANOVA), corrected for multiple comparisons according to GraphPad’s recommendations as follows: the Dunnett’s test, when comparing samples to one control in one category (for example, comparing all samples to WT at 18 hpi); the Tukey test, when comparing all samples to each other in one category (for example, comparing all 18 hpi samples to each other); or the Sidak method, when comparing all samples across all conditions (rarely used). When only two samples were compared, an unpaired Student’s *t* test was accomplished. Significance throughout the manuscript is shown as *, #, or ^ markings where *P < 0.05; **P < 0.01, ***P < 0.001, and ****P < 0.0001; n.s. means not significant; and, at times, the precise *P* value is written to highlight a trend toward significance. Occasionally, a two-tailed Pearson’s correlation analysis with 95% confidence interval was used to measure the strength and the direction of an association between two variables, and these are specified in the figure legends.

At least two individual clones were used per condition (ρ^0^ clones 6, 7, and 11 and ρ^+^ Uk cybrid clones 3 and 8) unless otherwise noted. Experiments were performed three independent times in at least triplicate, unless specifically noted otherwise, and the number of technical and well replicates (from a multiwell plate) indicated, as well as the number independent experiments plotted shown by *n*, at the end of each legend. For data shown in graphs, an unlabeled horizontal dotted line at 0 demarcates 0 and the labeled (l.o.d.) lines represent the limit of detection. When expanded clones are shown in supplemental figures, repeat data are indicated by a line or dotted graph and the related main figure panel specified. For data shown in violin plots, the solid green bar extending beyond the data points indicates the median, whereas black lines within indicate quartiles. In all graphs, the line or top of bar represents the mean and the error bars the SD, unless stated otherwise. The meaning of each individual data point and the respective statistical tests performed is defined in a graph-specific manner.

## Supplementary Materials

### The PDF file includes:

Supplementary Text Figs. S1 to S10 Legends for tables S1 to S5 References
