# Supplementary material for: Mitochondrial OXPHOS restricts SARS-CoV-2 replication
Source: Sci Adv. 2026 Jun 3;12(23):eadz3081. doi: 10.1126/sciadv.adz3081 (PMC13232578; doi:10.1126/sciadv.adz3081)
Supplement: Supplementary file 1 — Supplementary Text Figs. S1 to S10 Legends for tables S1 to S5 References [file sciadv.adz3081_sm.pdf]

Science Advances
AAAS

Supplementary Materials for

Mitochondrial OXPHOS restricts SARS-CoV-2 replication

Yentli E. Soto Albrecht *et al.*

Corresponding author: Douglas C. Wallace, wallaced1@chop.edu

Sci. Adv. **12**, eadz3081 (2026)
DOI: 10.1126/sciadv.adz3081

The PDF file includes:

Supplementary Text
Figs. S1 to S10
Legends for tables S1 to S5
References

Other Supplementary Material for this manuscript includes the following:

Tables S1 to S5

MATERIALS AND METHODS: SUPPLEMENTAL

Cell lines

All cell lines were incubated at 37 °C and 5% CO_2 in a humidified incubator, including during continuous live imaging experiments. Human lung adenocarcinoma A549 cells (ATCC certified), including A549 overexpressing ACE (A549-ACE2) cells, were a gift from Susan R. Weiss (77). A549 cells and all derivatives (A549-ACE2, chloramphenicol (CAP)-treated, mitochondrial DNA (mtDNA)-depleted (ρ^0), mtDNA-repleted (ρ^+ Uk cybrid) cells and OXPHOS inhibitor conditions) were maintained in RPMI containing high Glucose (4.5g/L), Pyruvate (1mM), and Uridine (50μg/ml) (GUP) media to overcome blocks in Oxidative Phosphorylation (OXPHOS), formulated from RPMI 1640 with ATCC modifications (Thermo Fisher Scientific; A1049101) supplemented with 50μg/ml uridine (Sigma-Aldrich; U3750-100G), 10% Fetal Bovine Serum (FBS), and 100U/ml of penicillin and 100μg/ml streptomycin (1% P/S) (Sigma-Aldrich; P4333-100ML). The osteosarcoma 143B thymidine kinase-deficient (TK-) (143B(TK-); ATCC; CRL-8303) ρ^+ Uk cybrids were maintained in Dulbecco's modified Eagle's medium (DMEM) GUP made from DMEM (Thermo Fisher Scientific; 10569044 or Fisher Scientific; 11-995-073) supplemented with 50μg/ml uridine, 10% FBS, and 1% P/S, as were human embryonic kidney HEK293T cells (ATCC; CRL-3216). African green monkey kidney Vero E6 cells (gifts from Susan Weiss and Florian Douam) were maintained in DMEM with 10% FBS, and 1% P/S, and VERO E6 TMPRSS2 cells (gift from Susan Weiss) were maintained in DMEM with 10mM HEPES (Sigma-Aldrich; 83264-100ML-F), 10% FBS, and 1% P/S. Huh7 cells (a well differentiated *hepatocyte* derived cellular carcinoma cell line, gift from Susan Weiss) were maintained in DMEM with 10% FBS and 1% P/S. The following volumes of complete media were used for routine cell culture: 5ml for T25 flasks, 15ml for T75, 25ml for T175, 50ml for T225. Cells were split when 70-95% confluency, during which cells were washed with

phosphate buffered saline (PBS) (Thermo Fisher Scientific; 14190250) and harvested with 0.25% trypsin-EDTA (Thermo Fisher Scientific; 25200114).

Viral stock preparation and plaque assay titration.

SARS-CoV-2 (USA-WA1/2020 strain, abbreviated as SARS-CoV-2 WA1, was obtained from the Biodefense and Emerging Infections Research Resources Repository (BEI) and propagated in VERO E6 TMPRSS2 cells (for all figures except **Figure 1F and S1D**, for which virus was propagated in VERO E6 cells). Recombinant SARS-CoV-2 expressing neongreen reporter protein in lieu of ORF7, gifted by Pei-Yong Shi (*68*), was obtained from Florian Douam and propagated in VERO E6 cells. HCoV-229E was propagated in Huh7 cells. Low MOI (0.01-0.1) infections were used to generate virus stocks, the cells seeded the day before in T175 flasks with 7.5-10 x 10^6 cells. Low passage virus P1-P2 was used to infect the flask of cells in 10ml of OPTI-MEM (Thermo Fischer Scientific; 51985034) for 1 h at 37 °C with gentle rotation, then 15ml of 2% FBS and 1% P/S-containing DMEM medium added (viral refeed medium). Sixteen-24 h later, the supernatant was discarded and 25ml of viral refeed media added to each flask. Two-four days later, when the cells exhibited excessive cytopathic effect (CPE), the culture medium was collected, passed through a 0.45μm filter, and stored at −80 °C as a P2 or P3 stock. SARS-CoV-2 WA1 grown on VERO E6 TMPRSS2 cells underwent two pooled collections.

To titrate the virus stock, we seeded Vero E6 cells into a 12-well plate (Corning; 3512 or CELLTREAT; 229112) at a density of 1.7-2 × 10^5 cells per well. The next day, the cells were incubated with serial 10-fold dilutions of the virus stock (150μl volume per well) for 1 h at 37 °C, overlaid with 1ml per well of medium containing a 1:1 mixture of 2× DMEM containing 4% FBS, and 2% P/S and 2.4% Avicel (DuPont; RC-581) and incubated at 37 °C for another 60-72 h. To visualize the plaques, the supernatant was discarded and the cell monolayer fixed with 1-2mL of

10% Neutral Buffered Formalin (NBF) (VWR; 10790-714) for 1 or more h and stained with 0.1% crystal violet in 20% ethanol 80% distilled water (dH_2O), at room temperature for at least 30 min. The number of plaques was counted (10 or more plaques per well preferred) and the virus titer was calculated.

Infections and other forms of virus quantification.

Infections were performed at a range of MOIs depending on the assay, in a 12-well plate seeded the day before with 1.45-1.75 x 10^5 cells/well for A549-ACE2 cells and derivatives or 2.0 x 10^5 cells/well for HEK-293Tcells. For a minimal number of repeated independent experiments in **Figure 1** (not shown), cells were infected in a 6-well plate seeded the day before with 4.0 x 10^5 cells/well A549-ACE2 cells and derivatives. Low MOI (MOI=0.2) was used when the kinetics of viral replication were assessed over time starting at 18hpi or later, including **Figures 1F-K, S1D-K, and 2E-J.** High MOI (2.0, 5.0, or 10.0) was used for early timepoints, assays where maximum infection was helpful, or both, such as **Figures 3A, 3H-I, S3E-F, S4F, 6A-B, S9C-D, S9F** (MOI=2.0), **Figures 3F-G, S3D, 4A-B, 5E, S5B, S6A, S9B** (MOI=5.0), **Figures 3B-E, S3A-C, S4A-E, 4C-G, 6C-D, S10** (MOI=10.0). Mock-infected controls received serum-free virus-free inoculum for the same amount of time, and the same viral refeed media.

Viral stocks were diluted to the required MOI by adjusting for doubling time (same adjustment in one experiment so the same amount of virus and inoculum volume was added to each well), and rapidly pipetted onto cells (free of aspirated media) with a multichannel pipetter to coordinate infections across plates. If any cells in an experiment already had mitochondrial dysfunction (CAP-treated, ρ^0 cells), the viral inoculum and wash media was FBS-free RPMI base media (with GUP additives) for all experimental samples; otherwise, OPTI-MEM was used. Post-absorption washes were accomplished with 750-900µL of serum free medium according to the following guidelines: 1 wash for experiments with MOI=0.2 and 5.0, as well as **Figure 3A**,

and 3 washes for all other experiments with MOI=2.0 and early virus release timepoints 2-10hpi. For assessing intracellular virus at 2-10hpi, cell monolayers were washed 3 times and the cells collected by scraping into 333µL of fresh viral refeed media and frozen at -80°C. Scraped plates were checked under brightfield (EVOSM5000) to ensure >90% scraping efficiency. Intracellular virus was released by three consecutive freeze-thaws (alternating 37°C and isopropanol-dry ice baths) followed by centrifugation (10,000 x rpm for 10 min) to clarify the supernatant, which was subsequently plaqued. Intracellular and extracellular viral titers were plotted as PFU/ml, adjusted by the counted plaque dilution and plaque assay viral inoculum volume; intracellular viral titers were additionally adjusted by the cell suspension volume of 333µL. For extracellular virus timepoints, at times indicated at least 100µL of virus-containing supernatant was collected and frozen at -80°C in the BSL-3 for storage until use. Viral titers were prepared in samples thawed no more than three times.

For glucose restriction studies in **Figure 6A-B and S9C-F**, 3 post-absorption washes were performed with 1% P/S serum-free and glucose-free RPMI media (to remove off all the glucose; Thermo Fisher Scientific; 11879020) supplemented with 1mM pyruvate, and 50µg/ml uridine, and 10mM Hepes. The viral refeed media was then supplemented with different experimental glucose levels (Alfa Aezar; J60067-AK) and 2% dialyzed FBS. 2-DG (vehicle control: water) and OXPHOS inhibitors (vehicle control: 100% ethanol) were added into the viral refeed media, "Inhibiting OXPHOS" as described above or "Inhibiting glycolysis," in **Figure 6A-D, S9C-D and F, as well as S10.** Thus, time post infection and time post treatment were the same for these conditions, since there was no drug pre-treatment.

Cell culture.

All cells were routinely tested for mycoplasma (Sigma-Aldrich; MP0035-1KT), with passage number tracked for each experiment, and experiments performed with the lowest passage

possible. Cells were seeded in a small range of cells/well or flask, in order to optimize cell confluency at the time of the experiment. Unless otherwise specified, the optimal cell confluency was 60-80 ±10% with every attempt made to match confluency across cell conditions within one experiment while seeding the same number of cells. This was complicated by the differences in cell size, doubling time, and cell count at confluency between conditions. Confluency was checked just prior to an experiment as well as after wells were manipulated with media changes. If any of these parameters were problematic, the cells were discarded and the experiment repeated.

For cell doubling calculations, cells were counted prior to seeding and at harvest; cells were seeded in a new flask. Doubling time was calculated per the following formula:

Doubling time (h) = culture time (h) / $\log_2$ (cell $number_{end}$ / cell $number_{start}$)

Inhibiting oxidative phosphorylation (OXPHOS).

mtDNA was depleted in A549-ACE2 cells to produce ρ^0 cells through serial passage in 50ng/ml Ethidium Bromide (EtBr; Sigma-Aldrich; E1510-10ML) in RPMI GUP media for 12 weeks (*116*, *117*). The EtBr stock was stored protected from light at 4°C, the 10mg/ml EtBr stock solution being filter sterilized and then diluted into medium. ρ^0 cells were clonally selected by limiting dilution and three individual clones (ρ^0 clones 6, 7, and 11) validated and selected for downstream assays.

CAP-treated cells (CAP cells) were generated by continuous treatment of A549-ACE2 WT cells with 50μg/ml chloramphenicol (CAP) (Sigma-Aldrich; C3175-100MG) added to RPMI GUP media culture media for at least 6 days and throughout all assays. A 10mg/ml CAP solution was made in water every 4 weeks, filter sterilized (0.22μm), and stored protected from light at 4°C. Treatment was started anew every 2-5 weeks to keep CAP-treated cells as low passage as

possible, and the maintenance and experimental media of CAP cells always contained 50μg/ml of CAP. Initially, the effects of 50, 100, and 200μg/ml of CAP on A549-ACE2 cells were initially studied, but since they all exerted the same effects on mitochondrial gene expression and respiration, the lowest dose (50μg/ml) was chosen for subsequent studies. One round of independent CAP validation experiments (Oroboros High Resolution Respirometry and Mitobiogenesis kits) was accomplished with all three CAP doses, and subsequent independent experiments with only the 50μg/ml dose.

OXPHOS complex I, III, and V inhibitors in this study are included: rotenone (Sigma-Aldrich; R8875-1G), antimycin A (Sigma-Aldrich; A8674-25MG) and oligomycin (Sigma-Aldrich; O4876-5MG), respectively. These were added to the viral refeed media during infection (after viral absorption and post-absorption washes, see below). In **Figure 1I-J**, CAP was also added in the viral refeed media. Since these OXPHOS inhibitor stock solutions were prepared in ethanol (EtOH), vehicle treatment was 1:100 of EtOH diluted in viral refeed media for panels of **Figures 1I-J, S1G and I-K, and 2J.** Stock solutions were stored at -20°C, vortexed vigorously and diluted in viral refeed media immediately before use at: 2.5μM (rotenone), 2.5μM (antimycin A), 1.25 μM (oligomycin). See below for infection details including viral refeed media base formulation.

Western blotting

A549 cells were seeded at 1-1.2 x 10^6 cells/well of a 6-well plate, then after 24h washed once with 1x PBS and lysed into 150μL of RIPA (50 mM Tris-HCl pH 7.5; 150 mM NaCl; 1 mM EDTA pH 8; 1% NP-40; 0.1% SDS) lysis buffer containing protease inhibitor (Cell Biolab; AKR-190). After 30 min on ice, lysates were centrifuged at 17,000 x g, 4°C, for 15 min, then supernatants transferred to a new Eppendorf tube. Protein concentrations were measured using Pierce BCA Protein Assay Kit (Thermo Fisher Scientific; 23225). The sample was diluted with 1x

PBS, and mixed with 4X Laemmli buffer (Bio-Rad; 1610737). Disulfide bonds were reduced with dithiothreitol (DTT; Thermo Fisher Scientific; 70726) or beta-mercaptoethanol (Sigma-Aldrich; M6250), then heated at 70°C for 10 min or 95°C for 5 min. A total of 10-30 µg of protein was separated on a Nativepage 3-12% Bis-Tris Gel (Thermo Fisher Scientific; BN1001BOX) or Nupage 4-12% Bis-Tris Gel (Thermo Fisher Scientific; NP0323BOX), along with PageRuler Plus (Thermo Fisher Scientific; 26619) or Precision Plus Protein Dual Color Standard (Bio-Rad; 161-0374) for size determination. After separation, samples were electro-transferred onto nitrocellulose membrane (or onto polyvinylidene difluoride (PVDF) membrane for ACE2 western blot) which were then blocked with 3-5% bovine serum albumin (BSA) and Tris-buffered saline with 0.1% Tween-20 (TBS-T) and probed with indicated primary antibodies (**Table S5**) overnight at 4°C. The next day, membranes were probed with secondary antibodies (**Table S5;** LI-COR Bioscience, Lincoln, Nebraska) and visualized using the LI-COR Odyssey M imaging system (Lincoln, Nebraska USA). Bands were quantified and ACE2 normalized to β-actin using ImageJ analysis software (*120*).

Seahorse metabolic assays.

Cellular oxygen consumption rate (OCR) and extracellular acidification rate (ECAR) were measured on 14-15 x 10^3 cells seeded the day before (20-26h). The cells were washed with filter-sterilized 37°C Seahorse XF RPMI medium (pH 7.4, Agilent Technologies; 103681-100) supplemented with 10mM glucose (or 50nM; Agilent Technologies; 103577-100), 2mM L-glutamine (Agilent Technologies; 103579-100), 1 mM pyruvate (with or without; Agilent Technologies; 103578-100), and 50µg/ml uridine. Ten well replicates of each cell line were seeded in Type I rat-tail collagen (50µg/ml, Corning; 354236) pre-coated (1 h at room temperature, followed by 3 PBS washes and air-drying) Seahorse XFe96 Cell Culture

Microplates (Agilent Technologies; 103793-100) and the plate was kept in a non-carbonated incubator for 30 min at 37 °C immediately prior to loading.

Cells were analyzed with an XFe96 Extracellular Flux Analyzer using the Wave desktop software (Agilent Technologies, Santa Clara, CA). For the Cell Mito Stress Test, three consecutive OCR measurements were obtained under basal conditions followed by the addition of 2μM oligomycin, then 150nM carbonyl cyanide 4-(trifluoromethoxy)phenylhydrazone (FCCP; Sigma-Aldrich; C2920-10MG) followed by a second 1.1μM dose of FCCP, and a combination of 2.5 μM rotenone and antimycin A. For the Glycolysis Stress Test, three consecutive ECAR measurements were obtained under basal conditions followed by the addition of 10mM glucose, which induces a sharp rise in glycolysis; followed by 2μM oligomycin and then 5mM 2-DG (Sigma-Aldrich; D8375-1G) to block glycolysis.

ECAR and OCAR data were normalized by nuclear count of matched, independently seeded Seahorse XFe96 microplates stained with NucBlue Live ReadyProbes dye (Thermo Fisher Scientific; R37605) and imaged with a 10X lens with the EVOS M5000 Imaging System (Thermo Fisher Scientific, Waltham MA) and quantification with Fiji analysis software (*121*). Delta ECAR was calculated by averaging the basal ECAR readings for each cell line within one independent experiment (“Basal ECAR”), subtracting average basal ECAR readings from individual glucose induction readings (n=10 wells), and plotting all 3 independent experiments per cell line (n=30 wells) on one graph (**Figure 5D**).

Oroboros high resolution respirometry.

High-resolution respirometry oxygen consumption was measured with the Oroboros Oxygraph-2k (Oroboros Instruments, Innsbruck, Austria) on intact and permeabilized cells. The Oroboros Oxygraph-2k was first calibrated to air for the cell culture medium (block temperature = 37 °C, stirrer speed = 750 rpm, oxygen sensor gain = 1, and data recording interval = 2 s). WT

or CAP-treated (for 24 h or 6+ days under continuous passage) cells were harvested, and $1.5X10^6$ million cells, resuspended in conditioned medium, were loaded into each chamber of the respirometer. Oxygen concentration (μmol/L) and oxygen flux [pmol/(s·10^6 cells)] were simultaneously recorded in real time. The first oxygen consumption plateau corresponds to routine respiration, determining respiration under endogenous substrates and energy demand; this value was plotted (**Figure S1B**). Specifically, 0.5μL of 5mM oligomycin was added to block complex V resulting in proton Leak respiration independent of complex V; followed by the stepwise addition of 1μL FCCP to reveal maximum ETS capacity, and finally 5μM antimycin A was added to determine background respiration, which is subtracted from the other respiratory states.

Two million WT, ρ^+ cybrid clones Uk3 and Uk8 cells or ρ^0 control cells were added to each Oroboros chamber for the permeabilized protocol (*122*). Air calibration was performed using the same settings as for intact cells, but in MiR05 respiratory buffer (0.5mM EGTA, 3mM $MgCl_2$, 60mM lactobionic acid, 20mM taurine, 10mM KH_2PO_4, 20mM Hepes, 110mM D-sucrose, and fatty acid-free bovine serum albumin [1g/L]). During the assay, 10μg/mL digitonin (Fluka; 37008), 5mM pyruvate (Sigma-Aldrich; P2256), 2mM malate (Sigma-Aldrich; M1000), 10mM glutamate (Sigma-Aldrich; G1626), 2.5mM ADP (Cal-biochem; 117105), 10mM succinate (Sigma-Aldrich; S2378), 5nM oligomycin, 1.5μM FCCP, 0.5μM rotenone, and 2.5 μM antimycin A were added sequentially.

DNA verification of A549-ACE2 nDNA.

The DNA identity of A549-ACE2 cybrids was determined from 500,000-1,000,000 cells extracted using the blood & tissue DNA extraction kit (Qiagen; 69506). 0.5μL of DNA (~12.5-50ng) was used in each of four distinct 30μL polymerase chain reaction (PCR) using amplifications of GoTaq Flexi DNA Polymerase (Promega; M8298) with annealing temperatures of 60C

and extension times of 1-2 minutes (min) and 30 seconds (s) **(Figure S2A-B)**, with the following primer sets: (A) 5'-GAAGCGAGAGATAGTTGGGG-3' and 5'-CATCATTGATACGGCTCCG-3'; (B) 5'-CCTTTAATTTCTTTGTCACTGCAC-3' and 5'-AGAATCGAGACCGAGGAGA-3'; (C) 5'-CCGTATGGCTTTCATTTTCTCC-3' and 5'-GGCAACACAGGCGAG-3'; (D) 5'-AACTCACCGCGACGTC-3' and 5'-GTGCACGAGGTGCCG-3'. Amplicons were resolved by 2% agarose gel electrophoresis (EMD Millipore; 8820-4L and Lonza; 50004) made with gel red nucleic acid stain (Biotium; 41003-1) and size compared to a 1kb ladder (Thermo Fisher Scientific; 10787018), and subsequently visualized on the Bio-Rad Universal Hood II Gel Molecular Imager Imaging System (Bio-Rad, Hercules, CA, USA).

mtDNA quantitative PCR (qPCR) and sequencing.

DNA was extracted from cell pellets and quantified by Taqman quantitative PCR (qPCR) with TaqMan Universal Master Mix II, with Uracil-N-glycosylase (UNG) (Thermo Fisher Scientific; 4440038) performed on 10ng of extracted DNA in 10µL reactions. The targets were mtDNA NADH dehydrogenase 5 (ND5; Thermo Fisher Scientific; 4331182, specify "Hs02596878_g1") and nDNA Glyceraldehyde 3-phosphate dehydrogenase (GAPDH; Thermo Fisher Scientific; 4331182, specify "Hs02786624_g1") and reactions were run on the ViiA 7 Real-Time PCR System (Thermo Fisher Scientific, Waltham, MA, USA) using default parameters. mtDNA levels were assessed by plotting fold change (2^-(ΔΔCT)) of ND5/GAPDH over WT A549-ACE2 (**Figure 1B and 2B**).

mtDNAs were sequenced following DNA quantification using Qubit dsDNA HS Assay Kit (Thermo Fischer Scientific; Q33231). The entire 16.5 kb mtDNA was amplified in one piece using long-range PCR. The primer pairs used were: LX-Human14204F: 5'-ATTACAATATATACACCAACAAACAATGTTCAACCAGTAACTAC-3' and LX-Human14204R 5'-TTTGTTGGTGTATATATTGTAATTGAGATTGCTCGGGGGAATAG-3'; LX-Human7274F 5'-

CTCTAACAGCAGTAATATTAATAATTTTCATGATTTGAGAAGCC-3' and LX-Human7274R 5'-TTATTAATATTACTGCTGTTAGAGAAATGAATGAGCCTACAG-3'; LX-Human15500F 5'-CGACCCAGACAATTATACCCTAGCCAACCCCTTAAACACCCC-3'; LX-Human15500R 5'-AGGGTATAATTGTCTGGGTCGCCTAGGAGGTCTGGTGAGAATAGTG-3'. Each PCR reaction was performed using the PrimeSTAR GXL DNA Premix (TaKara; R051B) in a total volume of 50µL containing 1× PrimeSTAR GXL Premix, 2% DMSO, 0.2 µM (10µL) of each primer, and 80ng of genomic DNA. The cycling conditions were: 94°C for 2 min; 35 cycles of 94°C for 30 s, annealing temperature at 10°C below melting temperature for 30 s, and 72°C extension for 20 s, with an increment of 20s for each cycle. PCR products were quantified by Qubit as before.

The PCR amplicons were diluted to 30ng/µL in 100µL using Tris-EDTA (TE) buffer (Alfa Aesar; J75793-AP), purified with 1x SMRTbell cleanup beads (PacBio; 102-158-300) and ran through Femto Pulse (Agilent; FP-1002-0275) to assess size distribution. DNA underwent A-tailing, and ligation of SMRTbell adapters using the SMRTbell prep kit 3.0 (PacBio;102-182-700). Libraries were treated with a nuclease cocktail to remove unligated DNA fragments and excess adapters, followed by size selection using a 35% dilution of AMPure PB beads (PacBio; 100-265-900) at a 3.1X volumetric ratio. The size-selected library were annealed to sequencing primers using the Sequel II Binding Kit 3.2 (Pacific Biosciences; 102-333-300), the annealed library bound to the Sequel II Polymerase 2.2 and loaded onto a single SMRT Cell 8M (Pacific Biosciences; 102-281-700), and sequencing performed on the PacBio Sequel IIe System (Pacific Biosciences, Menlo Park, CA) using the Sequel II Sequencing Kit 2.0 (PacBio; 102-194-400) with a 30 h movie time. Raw sequencing data was aligned to the revised Cambridge Reference Sequence (rCRS) and the output BAM file scanned for base changes, whereby changes with a frequency of 20% or above were recorded as variants. Positions with multiple bases (including the reference base) with a frequency of 20% or above were considered

heteroplasmy. A consensus sequence was obtained from the BAM file with samtools and used to obtain haplogroup identity on MITOMASTER (*123*).

Karyotyping.

Karyotype analysis (*124*) employed actively growing cells in log phase (70-85%) in a T25, fed the day before, and treated with 0.1µg/ml colcemide (Sigma-Aldrich; 10295892001) for 25 min. The cultures were gently washed with PBS, trypsinized, and the cells resuspended in a 0.075M KCl hypotonic solution for 7 min. The cells were fixed with methanol-acetic acid and stained with a Giemsa solution (Fisher Scientific; 612270100). Chromosomes were visualized on an inverted Zeiss microscope (Oberkochen, Germany) with an 63X oil immersion objective and the number of chromosomes manually quantified.

Assessing mtDNA versus nDNA protein expression using MitoBiogenesis In-Cell ELISA kit.

MitoBiogenesis In-Cell ELISA kit (abcam; ab140359) was used to assess the mtDNA translation of A549-ACE2 WT and CAP-treated cells by comparing the effects of CAP on the synthesis of the mtDNA coded COXI protein versus the nDNA coded SDH-A protein.

ACE2 quantification by transcript and protein.

ACE2 transcripts of mock-infected ρ^0, WT, and ρ^+ Uk cells were quantified by reverse transcriptase qPCR (RT-qPCR) from complementary DNA (cDNA) derived from cell pellets. The RNA used was from the same samples below employed for bulk RNA sequencing, and Taqman qPCR was performed on the targets of ACE2 (Thermo Fisher Scientific; 4331182, specify "Hs01085333_m1") normalized by hypoxanthine phosphoribosyltransferase 1 (HPRT; Thermo Fisher Scientific; 4331182, specify "Hs02800695_m1") and graphed as fold change (2^-(ΔΔCT)) over ρ^0 clone 11 cells.

ACE2 protein expression in A549 cells was quantified by western blot as described below. A total of 30 μg of protein, and Rabbit anti-ACE2 and mouse anti β-actin as specified in **Table S5,** were used for this particular blot.

RT-qPCR of viral ORF1a/b.

For reverse transcription-quantitative polymerase chain reaction (RT-qPCR) of cellular viral ORF1a/b RNA, infections were performed at an MOI=5 as described above, with mock-infected and UV-inactivated virus (the same virus used for infection was UV-inactivated for 10 min, designated "non-rep", (**Figure 3F**, **S3D,** and **S5B**) and then applied at an MOI=5) controls. Two to three different clones were included for ρ^0 and ρ^+ Uk cybrid cells, and three well replicates were included for all infected (and two for UV-inactivated virus treated and mock-infected) samples each for two independent experiments. At 6, 24, 48hpi, the cell pellets were collected directly into 350μL of RLT from RNAEasy Plus Mini kit (Qiagen; 74136) pre-prepared with 10μL/1ml beta-mercaptoethanol. Samples were lysed at room temperature for at least 30 min then frozen at -80°C. RNA extractions were later performed under RNAse-free conditions (Thermo Fisher Scientific; 7002) and RNA eluted in 30μL. The RNA was quantified by NanoDrop 8000 (Thermo Fisher Scientific, Waltham, MA, USA) and equimolar cDNA was made using the High-Capacity cDNA Reverse Transcription Kit (Thermo Fisher Scientific; 4368814). A Taqman qPCR was performed as previously described on 10ng of cDNA in 10μL reactions, where the target was viral ORF1a/b RNA using a proprietary probe (Themo Fisher Scientific; 4331182, specify "Vi07921935_s1 FAM/MGB"), along with a standard curve made with Taqman Comprehensive Microbiota Control (Thermo Fisher Scientific; A50383), containing 5 to 5 x 10^6 vRNA copies in 10-fold increments, and a 0 copies control. Mock-infected cells were included to observe background signal. Data were analyzed, quantified per standard curve, and plotted as viral RNA copies/μg of RNA.

IFN transcript induction.

Using the same cDNA as for determining viral RNA titers, Taqman assays were performed for Type I (IFN-β), Type III (IFN-λ), and the Interferon-Stimulated Gene (ISG) *IFIT1 compared to HPRT1 (*Thermo Fisher Scientific catalogue #4331182, respectively: Hs01077958_s1 FAM-MGB, Hs00601677_g1 FAM-MGB, and Hs01675197_m1 FAM-MGB with housekeeping Hs02800695_m1). Host gene expression was graphed as fold change (2^-(ΔΔCT)) over HPRT1 and cell type-specific respective mock-infected average.

Bulk RNA-sequencing (RNAseq).

Sample QC, library preparations, and sequencing reactions were conducted on the same extracted RNA from infected, mock-infected, and UV-inactivated virus treated cell pellets. Viral ORF1a/b and IFN transcripts were quantified at GENEWIZ, LLC./Azenta US, Inc (South Plainfield, NJ, USA) as follows:

Sample QC: Total RNA samples were quantified using Qubit 2.0 Fluorometer (Life Technologies, Carlsbad, CA, USA) and RNA integrity was checked with 4200 TapeStation (Agilent Technologies, Palo Alto, CA, USA). Samples were initially treated with TURBO DNase (Thermo Fisher Scientific, Waltham, MA, USA).

Library Preparation and Sequencing: Performed rRNA depletion was performed using QIAGEN FastSelect rRNA HMR Kit (Qiagen; 334386). RNA sequencing libraries were constructed with the NEBNext Ultra II RNA Library Preparation Kit for Illumina (New England Biolabs; E7770L). Enriched RNAs are fragmented for 15 min at 94 °C. First strand and second strand cDNAs were subsequently synthesized. cDNA fragments were end repaired and adenylated at 3’ends, and universal adapters are ligated to cDNA fragments, followed by index addition and library enrichment with limited cycle PCR. Sequencing libraries were validated

using the Agilent Tapestation 4200 (Agilent Technologies, Palo Alto, CA, USA), and quantified using Qubit 2.0 Fluorometer (Thermo Fisher Scientific, Waltham, MA, USA) as well as by quantitative PCR (KAPA Biosystems, Wilmington, MA, USA).

The sequencing libraries were multiplexed and clustered on the flowcell. After clustering, the flowcell was loaded on the Illumina NovaSeq X instrument and the samples were sequenced using a 2x150 Paired-End (PE) configuration. Image analysis and base calling were conducted by the NovaSeq Control Software (NCS). Raw sequence data (.bcl files) generated from Illumina NovaSeq was converted into fastq files and de-multiplexed using Illumina bcl2fastq 2.20 software. One mis-match was allowed for index sequence identification.

Data Analysis: After demultiplexing, sequence data was checked for overall quality and yield. Raw sequence reads were trimmed to remove possible adapter sequences and nucleotides with poor quality using Trimmomatic v.0.36. The reads were then mapped to the human GRCh38 reference genome available on ENSEMBL using the STAR aligner v.2.5.2b, resulting in BAM files. Unique gene hit counts were calculated by using feature Counts from the Subread package v.1.5.2, and only unique reads that fell within exon regions were counted. After extraction of gene hit counts, the gene hit counts table was used for downstream differential expression analysis. Using DESeq2, a comparison of gene expression between the groups of samples was performed. The Wald test was used to generate P values and Log2 fold changes. Genes with adjusted P values < 0.05 and absolute log2 fold changes >1 were called as differentially expressed genes for each comparison.

SARS-CoV-2 heatmap: Using pyrpipe (*125*), transcript reads were trimmed of adapter sequences, and mapped to the human transcriptome (GencodeV36) and SARS-CoV-2 transcriptome (ASM985889v3) using Salmon (*126*), following the pipeline in (https://github.com/jahaltom/COVID-19-Quantification). To increase mapping accuracy, the

human genome along with viral spike-ins from the Genomic Data Commons (GRCh38.d1.vd1) were used as decoys for Salmon. Transcript were quantified into Transcripts Per Million (TPM) and were transformed (log2(tpm+1)) and averaged across groups. The heatmap was made with R's pheatmap package.

PCA: Raw counts were input into DESeq2 (*127*) and were transformed via VST. All genes were used in PCA with no significance cutoff.

Venn Diagrams: Genes from the DESeq2 (*127*) results (p adjusted (padj) <=0.05) were used to construct Venn diagrams using venny (https://bioinfogp.cnb.csic.es/tools/venny/), then manually reconstructed.

Volcano plots: Differential expression of individual genes from DESeq2 (*127*) were represented in volcano plots generated using the EnhancedVolcano package in R with a padj threshold 10^{-3} from our previously published gene lists (*21*).

Dot plots and GoEnrichmentResults(excel): Enriched GO terms were identified from the DESeq2 data (*127*) via ClusterProfiler's (*128*) gseGO function set to the following parameters; keyType = "SYMBOL", nPerm = 10000, minGSSize = 3, maxGSSize = 800, pvalueCutoff = 0.05, verbose = TRUE, OrgDb = org.Hs.eg.db, pAdjustMethod = "BH".

Heatmaps: Using the DESeq2 (*127*) results from the WT vs ρ^0 clone 11 comparison, genes from relevant Hallmark gene lists (*129*) were filtered for a padj <=0.05 (padj filtering was done for mtDNA vs nDNA genes heatmap only). This gene list was intersected with VST expression data for all groups, and genes with the greatest |Log2FC| were selected. VST values were averaged across groups.

Transmission electron microscopy (TEM).

Cells were seeded at 4 x 10^5 cells/well (6-well plate) in duplicate followed by infection with SARS-CoV-2 WA1 at MOI=2.0 (or mock-infected). At 24hpi, the supernatant was removed and cells fixed in 1ml/well 4% paraformaldehyde (PFA; Alfa Aesar; J61899-AP) at room temperature for 30 min. Cells were scraped into Eppendorfs, which were briefly centrifuged at 1,000 x g for 5 min, the PFA aspirated and the cells resuspended in TEM fixative (2.5% glutaraldehyde, 2.0% paraformaldehyde in 0.1M sodium cacodylate buffer, pH7.4) overnight at 4℃. After subsequent buffer washes, the samples were post-fixed in 2.0% osmium tetroxide for 1 h at room temperature and rinsed in dH2O prior to *en bloc* staining with 2% uranyl acetate. After dehydration through a graded ethanol series, the tissue was infiltrated and embedded in EMbed-812 (Electron Microscopy Sciences; 14120). Thin sections were stained with uranyl acetate and lead citrate and examined with a JEOL 1010 electron microscope (JEOL UESA, Peabody, MA, USA) fitted with a Hamamatsu digital camera (Bridgewater, NJ, USA) and AMT Advantage NanoSprint500 software.

Brightfield, fluorescence, and confocal microscopy.

Brightfield and EVOS M5000 representative viral fluorescence: Brightfield images of cells in **Figures 1H, S1C, S1G,** and **S9E** were acquired using the EVOS M5000 Imaging System, all with a 10X lens except for **S9E,** with a 4X lens. Fluorescence for **Figure 1H** was acquired in the GFP channel with the same 10X lens magnification.

Mitochondrial mass: Cells were seeded 12,000-16,000 cells/well on a rat-tail collagen coated (see above) glass-bottom 96-well plate (Cellvis; P96-1.5H-N). The next day, cells were live stained with MitoTracker CMXRos Red (Thermo Scientific; M7512) and NucBlue (Thermo Fisher Scientific; R37605) for 30 min at 37°C, and media replaced with sterile-filtered Tyrodes' GUP buffer (135mM NaCl, 5mM KCl, 1.8mM $CaCl_2$, 20mM HEPES, 25mM glucose, 1mM $MgCl_2$, 1mM pyruvate, 50μg/ml uridine, pH 7.4) at 37°C. Cells were imaged using a Zeiss LSM

710 laser scanning confocal microscope with a PlanApo 63x/1.40 oil immersion objective in combination with the Zen 12 software (Oberkochen, Germany), for image acquisition. Some independent experiments were imaged with EVOS M5000 fluorescent microscope; representative images are shown in **Figure S2H**, where 8-bit images were 136.44 x 136.55μm (1260 x 1261 pixels) in size, developed as maximum projections from Z stacks, 7 steps of 1μm spanning a distance of 6.0μm.

dsRNA staining, imaging, and quantification: Cells were seeded 12,000-16,000 cells/well on a rat-tail collagen coated glass-bottom 96-well plate the day before and infected with 229E at an MOI=2 (**Figure S4F**) or SARS-CoV-2 WA1 at MOI=10 (below). For **Figure 6C-D and S10**, cells were infected with SARS-CoV-2 WA1 MOI=10 and 0, 1, or 10mM 2-DG added to the viral refeed media as previously described, then fixed at 12hpi before staining. Mock-infected wells for all cell types were also included. The primary and secondary antibody combinations were optimized for every antibody used with at least four permutations. At 6hpi (**Figure S4A**), 10hpi (**Figure S4B-E**), 12hpi (**Figure 6C-D and S10)** or 18hpi (**Figure 3B-E, S3A-C, 4C-D, S4B and S4E-F**). Cells were fixed with 10% NBF for 1 h or more, washed with PBS three times and stored in PBS at 4°C degrees protected from light until assayed up to 14 days later. The cells were permeabilized with PBS + 0.1% Triton X-100 (v/v) (Sigma-Aldrich; X100-500ML) for 1 h at room temperature then blocked in PBS + 5% goat serum (Jackson ImmunoResearch; 005-000-121) and 0.1% TWEEN-20 buffer (Calbiochem; 655204), hereby 'blocking buffer' for 1 h at room temperature. Primary antibody incubation was done overnight at 4ºC followed by secondary incubation with fluorescently conjugated antibodies and Hoechst counterstain for 6 min at room temperature and protected from light, all in blocking buffer. **Table S5** lists the manufacturer and dilution used for each antibody. Secondary antibody only controls were included and imaged as background. Washes occurred in PBS with 1:100 dilution of blocking buffer, 3 each between primary and secondary and after secondary. Confocal images were acquired using Zeiss LSM

710 laser scanning confocal microscope with a PlanApo 63x/1.40 oil immersion objective, in combination with the Zen 2012 software (Oberkochen, Germany), for image acquisition.

For all images involving dsRNA staining and acquisition on the Zeiss confocal microscope, Z stacks were acquired with the subsequent parameters. For images in **Figures S2I, 3B-E, S3A-C, S4A-E,** 8-bit images were 136.44 x 136.55μm (1260 x 1261 pixels) in size, developed as maximum projections from Z stacks, 10 steps of 1μm spanning a distance of 9.0μm. For **Figure S4F,** 8-bit images were 134.84 x 134.95μm (1260 x 1261 pixels) in size, developed as maximum projections from Z stacks, 9-13 steps spanning a distance of 4.0-6.0μm. For **Figure 4C,** 8-bit images were 134.84 x 134.95μm (1260 x 1261 pixels) in size, developed as maximum projections from Z stacks, 9-13 steps spanning a distance of 4.0-7.0μm. For representative images in **Figure 4D,** 8-bit images were 224.74 x 224.92μm (1260 x 1261 pixels) in size, developed as maximum projections from Z stacks, 10 steps of 1μm spanning a distance of 9.0μm.

Image quantification from Zeiss-acquired images (**Figure 3B-E, S3A-B and S4C-E**) was obtained using Fiji analysis software (*121*) and semi-automated macro programs to accelerate manual analysis, with code available upon request. For all quantification experiments, images were acquired from at least 3 separate wells and 2-3 independent experiments. The investigator was blinded to the hypothesis. The values from Fiji were exported into Microsoft Excel and analyzed with GraphPad prism 10 for the specified statistical approaches.

For quantification of subcellular organelle locations from Zeiss-acquired images, a Z projection with maximum intensity (as described above) was formed for each channel, and the channels divided into their respective colors for individual analysis. The RTN3 channel's brightness and contrast was manually adjusted to optimize visualization for manual tracings. No contrast or brightness adjustment to channels for dsRNA were used to ensure the accuracy of

Mean Fluorescence Intensity (MFI) measurement. Specifically, regions of interest (ROIs) within the dsRNA channel within individual cells were selected, and the area and MFI measured, providing a quantifiable metric for the dsRNA content per cell. For **Figure 3B and E,** the ER area was measured by selection of corresponding regions within the RTN3 channel. To normalize the replication area (dsRNA) to the ER area (RTN3), the ratio of the replication area to the ER area was calculated. Each point plotted for figures **Figure 3B-E and S4C-E** represents a separate cell.

To measure the distance from the nuclear edge to the dsRNA replication sites for **Figure S3A-B**, a custom macro was adapted from Ved P. Sharma's original script (*130*). First, the nucleus was manually selected in the DAPI channel and saved as a circular region of interest (ROI). The dsRNA channel was used to manually pinpoint 25 sites evenly spaced around the dsRNA region edge and saved to the ROI manager. The macro program found the 25 corresponding sites in dsRNA channel and output the shortest distance from each point to the edge of the nucleus for further analysis. If a point lay within the nucleus, the distance was set to zero. This method allowed for precise measurement of the spatial distribution of replication sites relative to the nuclear periphery. Ten cells from each type were quantified two independent times, with each point plotted independently as a point between a dsRNA edge and the nuclear edge.

For **Figure 6C-D and S10**, the images were analyzed using the following Custom Module as described: The DAPI channel was used to identify nuclei according to the following parameters: objects between 8μm and 20μm in diameter and greater than 20 grey level units in intensity above the local background. The TexasRed channel was used to identify dsRNA signal. To detect dsRNA+ signal, an adaptive threshold was performed on the TexasRed channel, creating a binary mask over objects between 1μm and 2μm in diameter and greater than 200 grey level units in intensity above the local background. To determine the sum region of the

dsRNA foci within each cell – which represents the DMV – the TexasRed binary mask was then dilated by a size of 10 pixels in all directions, forming a continuous merged region of TexasRed signal for each dsRNA+ cell called “Modify TexasRed”. To minimize visual artifacts, any objects from the “Modify TexasRed” that were not at least 125 pixels in area were removed from the binary mask, and the result was called “Final TXRed signal”. Nuclei which overlapped with “Final TXRed signal” were considered to be infected cells, while nuclei which did not overlap with “Final TXRed signal” were considered uninfected. Perfect infection was determined at the image level as the percentage of infected cells within each image. Data was plotted in GraphPad Prism 10.0 as one representative independent experiment where each replicate represents an individual field image.

For **Figure 6C-D and S10**, to perform quantifications of DMV morphology and MFI, the same images and Custom Module analysis parameters used for quantification of percent infection yielded cell-level readouts of average fluorescence intensity, total area, and shape factor of each dsRNA+ object. Total area, average fluorescence intensity, and shape factor of each dsRNA+ object were automatically measured from the Custom Module’s built in analysis. To calculate MFI of each dsRNA+ object, the average fluorescence intensity of the dsRNA+ object was divided by the total area of that same dsRNA object. Data was plotted in GraphPad Prism 10.0 as one representative independent experiment where each replicate represents an individual infected cell. Representative images were assembled using FIJI/ImageJ. Within each field of view, each channel’s images were pseudo-colored to match a representative color. The two channels were then merged, and brightness, contrast and color adjustments were performed equally on all images within the same plate. To improve visibility of cells, 50 x 50μm fields of view were cropped from select regions, and a scale bar was added manually.

Percent infection quantification by CellProfiler: For **Figure 1J** and **S1F,** cells were infected with SARS-CoV-2 neongreen reporter virus at an MOI=0.2 in a 12-well plate, fixed at 48hpi, and

counterstained with Hoechst as previously described. Cells were imaged with BioTek Cytation 5 Cell Imaging Multimode Reader (Santa Clara, CA, USA), 25 images at 4X magnification per well, and quantified with CellProfiler (*131*) as percent infection: (# green fluorescent events (reporter virus) / # nuclear events (Hoescht)) * 100. Each dot is an individual well replicate with all 25 field images averaged into one value.

ImageExpress imaging and quantification: ImageXpress Confocal HT.ai High-Content Imager

Images were captured using the ImageXpress Confocal HT.ai High-Content Imaging System (Molecular Devices, San Jose, CA, USA) on 12,000-16,000 cells/well seeded on a rat-tail collagen coated glass-bottom 96-well plate as previously described and manipulated as described below, unless otherwise mentioned. Antibody specifics are found in **Table S5**. Images were acquired using Z stacks whenever possible as specified, with an AndorSdk3 Camera. Images were analyzed using MetaXpress High Content Image Acquisition and Analysis software (Molecular Devices, San Jose, CA,USA) by the specified modules and parameters. The data was exported into Microsoft excel and statistical analyses applied in GraphPad prism 10.0. Live imaging was performed at 37 °C and 5% CO_2 in a humidified chamber. Fixed cell imaging was performed at room temperature lacking CO_2 and humidification, with 270µl of sterile de-ionized water added to each outer well to minimize evaporation effects for the duration of each experiment.

Percent infection quantification by ImageExpress on 12-well plates: For **Figure S1J-K**, cells from **Figure S1I** were fixed at 48hpi and stained for SARS-CoV-2 nucleocapsid and counterstained with Hoechst as previously described. Cells were imaged with a 4X Plan Apo objective lens in the DAPI (excitation: 377/54 nm and emission: 447/60 nm) and FITC (excitation 465/40 nm and emission 525/30 nm) channels, with 16 images per well, 3 wells per

condition. Both channels were used to establish their respective laser autofocus offsets. The image size was 1229 x 1229pixels with 16-bit gain and a 540 MHz digitizer applied. Imaging software was MetaXpress 6.7.2.290. The images were analyzed using the Multi Wavelength Cell Scoring module in standard mode. The “all Nuclei” channel was DAPI and used to identify cells according to the following parameters: objects between 3μm (5 pixels) and 20μm (31 pixels) in diameter and greater than 300 grey level units in intensity above local background. These parameters were required before a cell could be identified as infected by the FITC fluorescent channel. The FITC channel was used to classify infected (FITC+) cells according to the following parameters: objects between 3μm (5 pixels) and 25μm (38 pixels) in diameter and greater than 50 grey level units in intensity above local background, with a minimum stained area of 1μm^2 (2 pixels). The remaining FITC- objects were set to uninfected cells. From this automated analysis, the following image-level readouts were measured and used for downstream analysis: “% Positive W2” (MultiWaveScoring; or number of FITC+ cells/ total nuclei expressed as a percentage). Data was plotted in GraphPad prism 10.0 as one representative independent experiment where every dot represents an individual field image. Non-plotted independent experiments exhibit slightly different image size and object call parameters, yet the same analysis pipeline and statistics were applied.

Reporter virus percent infection and MFI: For **Figure 2G-I,** cells were infected with SARS-CoV-2 neongreen reporter virus at an MOI=0.2 in coated glass-bottom 96-well plates, 200μl of viral refeed media added to each well, and automated live-imaging of nine 10X images from the center of each well performed every 2 h up to 60hpi with a 10X Plan Apo Lambda objective lens in the FITC (excitation 465/40 nm and emission 525/30 nm) and Transmission Light (TL) 25 plane illumination setting (brightfield). Both channels were used to establish their respective laser autofocus offsets, and a 60μm pinhole was used. Z stacks included 3 steps of 3.6μm in size spanning a distance of 7.2μm around the autofocus plane (occurring prior to each image

acquisition) for both channels. The image size was 1741 x 1741 pixels with 16-bit gain. Imaging software was MetaXpress 6.7.2.290. The images were analyzed using the Multi Wavelength Cell Scoring module. The “all Nuclei” channels were brightfield images. Standard mode was used to identify cells according to the following parameters: objects between 10μm (15 pixels) and 180μm (264 pixels) in diameter and greater than 400 grey level units in intensity above local background. These parameters were required before a cell could be identified as infected by the FITC fluorescent channel. The FITC channel was in standard mode was used to classify infected (FITC+) cells according to the following parameters: objects between 5μm (7 pixels) and 85μm (125 pixels) in diameter and greater than 500 grey level units in intensity above local background, with a minimum stained area of 3μm^2 (6 pixels). The remaining FITC- objects were set to uninfected cells. From this automated analysis, the following image-level readouts were measured and used for downstream analysis: “% Positive W2” (MultiWaveScoring; or number of FITC+ cells/ total cells expressed as a percentage) and “Positive W2 Mean Stain Aver Intens” (MFI normalized by FITC+ cell area). Data was plotted in GraphPad prism 10.0 as one representative independent experiment where every dot is an average of all 9 images and 4 wells per cell condition, the error bars representing the standard error of the mean.

Nuclear morphology quantification: For **Figure S2J-K**, nuclear morphology of cells were reanalyzed from another experiment. Cells were previously mock-infected in coated glass-bottom 96-well plates, fixed at 18hpi and counterstained with Hoechst (specified in **Table S5**). Cells were imaged with 6 images per well, 2 wells per condition, with a 40X Plan Apo Lambda objective lens in the DAPI (excitation: 377/54 nm and emission: 447/60 nm) channel, which was used to establish a laser autofocus offset, and a 42μm pinhole. Z stacks were 9 steps of 1μm in size spanning a distance of 7.96μm around the autofocus plane (occurring prior to each image acquisition), and image size was 2048 x 2048 pixels with 16-bit gain. Imaging software was MetaXpress 6.7.1.157. The images were analyzed using a Custom module, only on the DAPI

channel. Nuclei were classified according to the following parameters: objects between 5µm and 25µm in diameter and greater than 2,000 grey level units in intensity above local background, and an adaptive threshold. Then, the "fill holes" module was used with an adaptive threshold, followed by a filter mask to remove items with an area larger than 250μm^2 (this eliminated nuclei clustered too close together counted as one large object). From this automated count and morphologic analysis, the following cell-level readouts were measured and used for downstream analysis: "Form Factor" ((4 * π * area) / (perimeter^2)) and "Ell form factor" (Nuclear length/breadth). Data was plotted in GraphPad prism 10.0 as one representative independent experiment where every dot is a nucleus.

IFIT3 quantification of infected cells: For **Figure 4D-G**, cells were infected with SARS-CoV-2 WA1 MOI=10 in coated glass-bottom 96-well plates, fixed at 18hpi, stained with anti-dsRNA and anti-IFIT3 and counterstained with Hoechst (**Table S5**). Cells were imaged with 49 images per well, 6 wells per condition, with a 40X Plan Apo Lambda objective lens in the FITC (excitation 465/40 nm and emission 525/30 nm), Texas Red (excitation: 560/32 nm and emission: 624/40 nm), and DAPI (excitation: 377/54 nm and emission: 447/60 nm) channels, each used to establish a laser autofocus offset, and a 42µm pinhole. Z stacks were 9 steps of 1µm in size spanning a distance of 7.96µm around the autofocus plane (occurring prior to each image acquisition). Image size was 2048 x 2048 pixels with 16-bit gain, confocal module dichroic wheel PENTA-BAND 5084036, and 540 MHz digitizer. Imaging software was MetaXpress 6.7.1.157. The images were analyzed using the Multi Wavelength Cell Scoring module. The "all Nuclei" channel was DAPI, "W2" dsRNA, and "W3" IFIT3, and fast mode used for all analyses. Nuclei were classified along the following parameters: objects between 5µm (29 pixels) and 25µm (147 pixels) in diameter and greater than 3,000 grey level units in intensity above local background. This was required before a cell could be identified as infected by the dsRNA channel. The second channel was dsRNA and used to classify infected (DAPI+ and dsRNA+)

cells along the following parameters: objects between 30µm (176 pixels) and 50µm (294 pixels) in diameter and greater than 5,000 grey level units in intensity above local background, with no minimum cutoff. The third channel was IFIT3 and used to classify the ISG responding (DAPI+ and IFIT3+) cells along the following parameters: objects between 30µm (176 pixels) and 100µm (587 pixels) in diameter and greater than 2500 grey level units in intensity above local background, with no minimum cutoff. From this automated analysis, the following cell-level readouts were measured and used for downstream analysis: (# FITC+ cells/ # dsRNA cells) expressed as a percentage, "Cell: W3 Stained Area (MultiWaveScoring)" and "Cell: W3 Stained Integr Intensity (MultiWaveScoring, otherwise known as total pixels/cell)". Data was plotted in GraphPad prism 10.0 where every dot is a cell. Representative images in **Figure 4D** was acquired using maximum projections with the Zeiss confocal microscope.

NLRP3 ASC speck quantification: For **Figure S6B-C**, cells were seeded in coated glass-bottom 96-well plates, and the next day treated with 1µg/ml Lipopolysaccharides (LPS, Sigma-Aldrich; L2630-10MG) in 100µl/well of serum free media for 3 h at 37°C (in the incubator). 20µM nigericin (Sigma-Aldrich; N7143-5MG) was added directly to wells and mixed gently 3 times, followed by 45 min at 37°C (in the incubator). Cells were fixed and stained with anti-ASC and counterstained with Hoechst (**Table S5**). Cells were imaged with 16 images per well, 2 wells per condition, with a 20X Ph1 S Plan Fluor ELWD ADM objective lens in the FITC (excitation 465/40 nm and emission 525/30 nm) and DAPI (excitation: 377/54 nm and emission: 447/60 nm) channels, each used to establish a laser autofocus offset, and a 42µm pinhole. Z stacks were 7 steps of ~1µm in size spanning a distance of ~6µm around the autofocus plane (occurring prior to each image acquisition). Image size was 1229 x 1229 pixels with 16-bit gain and confocal module dichroic wheel PENTA-BAND 5084036. Imaging software was MetaXpress 6.7.2.290. The images were analyzed using the Granularity module in standard mode. The "granule" channel was "FITC", and nuclear stain was selected as "DAPI". Granules were

classified along the following parameters: objects between 1μm (3 pixels) and 3μm (9 pixels) in diameter and greater than 5,000 grey level units in intensity above local background. Nuclei were classified along the following parameters: objects between 8μm and 50μm in diameter and greater than 2,000 grey level units in intensity above local background. From this automated analysis, the following image-level readouts were measured and used for downstream analysis: "Granules (Granularity)"as a granule count per field image. Data was plotted as an average of all fields representing one independent experiment in GraphPad prism 10.0.

ImageExpress imaging and quantification: ImageXpress Micro Confocal

IFIT3 and MAVS quantification of mock-treated cells: For **Figure S7**, cells were mock-treated in coated glass-bottom 96-well plates, fixed at 24 hours post treatment, stained with anti-IFIT3 or anti-MAVS and counterstained with Hoechst (**Table S5**). Cells were imaged at 49 images per well, 10 wells per condition, with a 40X Plan Apo Lambda objective lens in the FITC (excitation: 465/40 nm and emission: 525/30 nm) and DAPI (excitation: 377/54 nm and emission: 447/60nm) channels, each used to establish a laser autofocus offset, and a 60μm confocal pinhole. Z stacks were 7 steps of 1.5μm in size spanning a distance of 9μm around the autofocus plane (occurring prior to each image acquisition). Max projections within each field of view were automatically assembled and saved for downstream analysis. Image size was 2048 x 2048 pixels, corresponding with 354.91 x 354.91μm. Imaging software was MetaXpress 6.7.1.157.

The images were analyzed using a Custom Module as described: Nuclei were first identified within the DAPI channel as round objects between 8 and 30μm in width and 10 grey level units in intensity above the local background. To identify either IFIT3 or MAVS signal, an adaptive threshold was performed on the FITC channel to create a binary mask over objects between a width of 0.66 and 2.22 μm and 150 grey level units of intensity (for IFIT3) or 50 grey level units

of intensity (for MAVS) above the local background. To exclude noise from the analysis, the binary mask of the FITC signal was then dilated by 5 pixels in each direction, and mask objects which were below 100 pixels in size were excluded from downstream processing. The Boolean OR operator was then used on the nuclei objects and filtered FITC binary mask to create a mask covering both the FITC and DAPI signal, which was called the “FITC.DAPI mask”. Within each field of view, the following measurements were derived from the FITC.DAPI mask: sum integrated intensity of the FITC signal, sum area of the FITC signal, and nuclei count of the DAPI signal, producing image-level data. MFI of the FITC signal was calculated as sum intensity of the FITC signal divided by sum area of the FITC signal for each field of view. Average area of the FITC signal per cell was calculated as sum area of the FITC signal divided by the nuclei count within the same field of view. Data was plotted in GraphPad Prism 10.0 as one representative independent experiment where every replicate is the measurements derived from an individual field of view, the error bars representing the standard error of the mean. Representative images were assembled using FIJI/ImageJ. Within each field of view, each channel’s images were pseudo-colored to match a representative color. The two channels were then merged, and brightness, contrast and color adjustments were performed equally on all images within the same plate. To improve visibility of cells, 100 x 100μm fields of view were cropped from select regions, and a scale bar was added manually. A Grubb's test was performed to detect outliers, and data points with an alpha value below 0.05 were not plotted on the quantification graphs. Less than 5 data points were removed.

Cytotoxicity assay.

Supernatant samples (fresh or stored at 4°C for up to 10 days) were assayed for cytotoxicity via lactate dehydrogenase (LDH) quantified using an LDH Cytotoxicity Detection Kit (Sigma-Aldrich; 11644793001). Blank condition-specific media was used to subtract background

signal. Percentage cytotoxicity was calculated relative to ceiling LDH release values, as quantified from cultures treated with Triton X-100 for the duration of the experiment (>8 h).

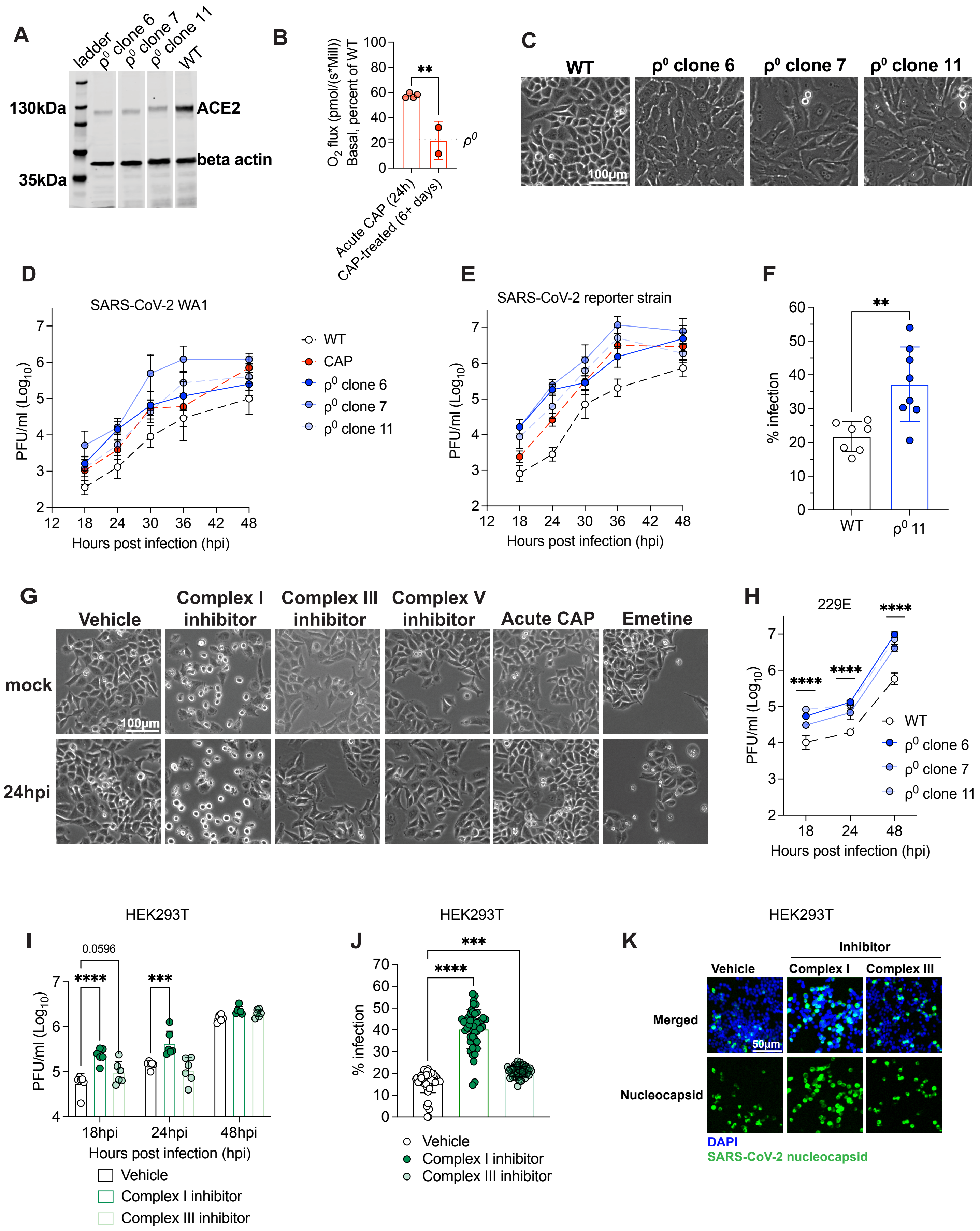
A
ladder
ρ0 clone 6
ρ0 clone 7
ρ0 clone 11
WT
130kDa
ACE2
35kDa
beta actin
B
O2 flux (pmol/(s*Mill)) Basal, percent of WT
**
ρ0
Acute CAP (24h)
CAP-treated (6+ days)
C
WT
ρ0 clone 6
ρ0 clone 7
ρ0 clone 11
100μm
D
SARS-CoV-2 WA1
PFU/ml (Log10)
Hours post infection (hpi)
WT
CAP
ρ0 clone 6
ρ0 clone 7
ρ0 clone 11
E
SARS-CoV-2 reporter strain
PFU/ml (Log10)
Hours post infection (hpi)
F
% infection
**
WT
ρ0 11
G
Vehicle
Complex I inhibitor
Complex III inhibitor
Complex V inhibitor
Acute CAP
Emetine
mock
24hpi
100μm
H
229E
****
PFU/ml (Log10)
Hours post infection (hpi)
WT
ρ0 clone 6
ρ0 clone 7
ρ0 clone 11
HEK293T
I
0.0596
****
***
PFU/ml (Log10)
18hpi
24hpi
48hpi
Hours post infection (hpi)
Vehicle
Complex I inhibitor
Complex III inhibitor
HEK293T
J
***
****
% infection
Vehicle
Complex I inhibitor
Complex III inhibitor
HEK293T
K
Inhibitor
Vehicle
Complex I
Complex III
Merged
Nucleocapsid
50μm
DAPI
SARS-CoV-2 nucleocapsid

Supplementary Figure 1: A wide breadth of OXPHOS inhibition enhances coronaviral replication.
(A) ACE2 protein expression by western blot, with β-actin as loading control (30 µg loaded). Representative of n=3.
(B) OCR by Oroboros respirometry (basal respiration) on intact cells treated with 50, 100, or 200µg/ml CAP for 24 hours (Acute CAP) or with 50µg/ml for 6+ days. Dotted line = ρ^0 baseline. n=2, where Acute CAP conditions are grouped, and one independent experiment is the permeabilized condition.
(C) Brightfield images at confluence of A549-ACE2 cells and derivatives. Representative mock of Figure 1H.
(D) – (E) Figure 1F-G expanded clones, where repeat data is shown with a dashed line. 4 well replicates, n=2, confirmed a third time by select samples and timepoints.
(F) Percent infection following MOI=0.2 at 48hpi by SARS-CoV-2 neongreen, counterstained with Hoechst. n=2.
(G) Representative images of mock-treated and infected cells at 24hpi from Figure 1I.
(H) Figure 1K expanded clones, where repeat data is shown with a dashed line; significance applies to every clone to WT comparison. 2-4 well replicates, n = 2 of 3 shown.
(I) –(K) SARS-CoV-2 WA1 replication in HEK-293T cells treated with OXPHOS complex inhibitors in viral refeed media, and supernatant viral titers by plaque assay **(I)** or percent infection assayed with representative images shown **(J, K)**. 3 well replicates, n=2.
Mean + SD where applicable. Where statistics are shown, an unpaired student's t-test (S1B and F) or two-way ANOVA was performed compared to WT or vehicle where * = $p < 0.05$; ** = $p < 0.01$; *** = $p < 0.001$; **** = $p < 0.0001$. Image brightness adjusted simultaneously for visibility.

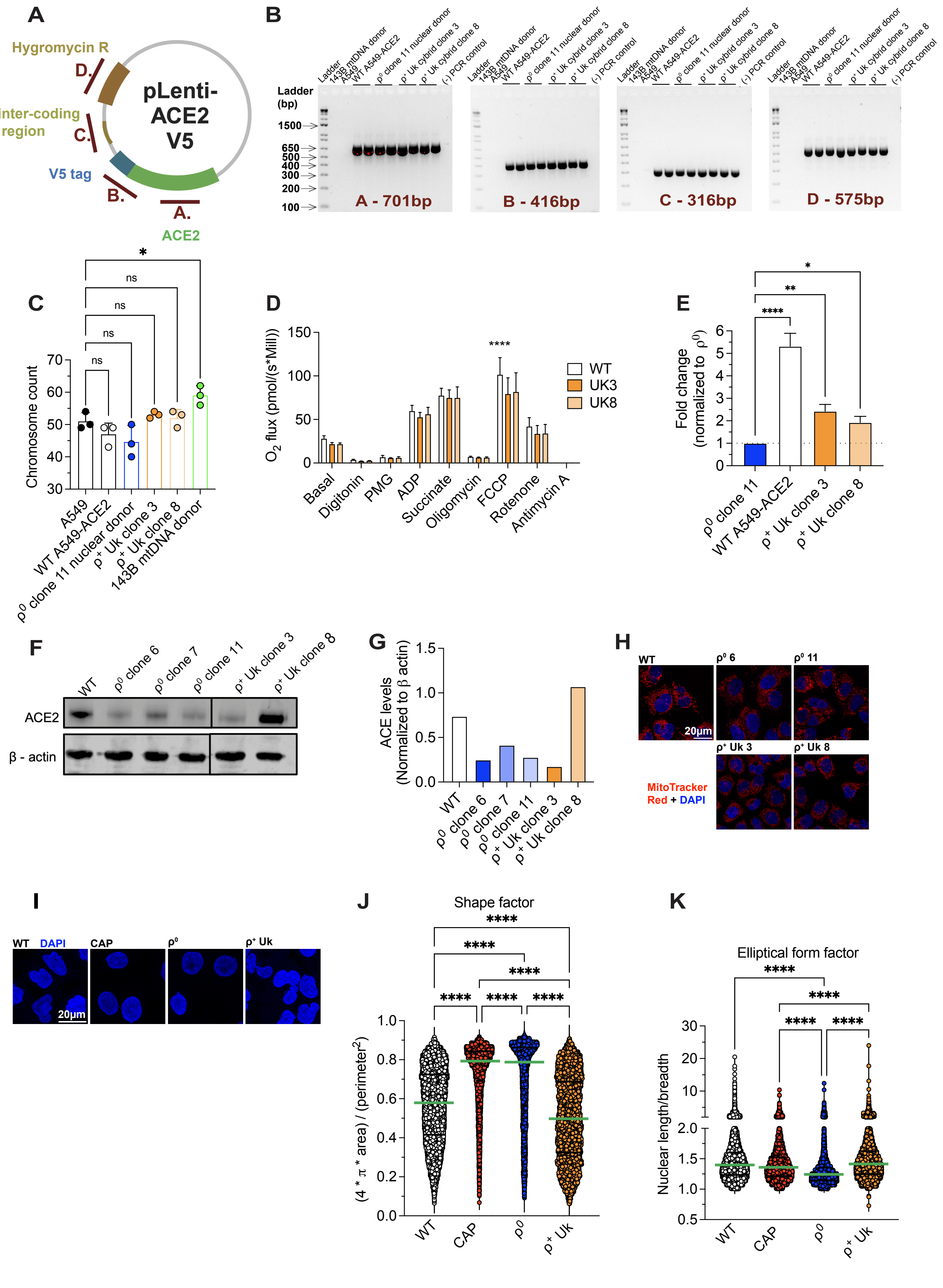
A
B
C
D
E
F
G
H
I
J
K
pLenti-ACE2 V5
Hygromycin R
inter-coding region
V5 tag
ACE2
A - 701bp
B - 416bp
C - 316bp
D - 575bp
Chromosome count
O2 flux (pmol/(s*Mill))
Fold change (normalized to ρ0)
ACE levels (Normalized to β actin)
ACE2
β - actin
MitoTracker Red + DAPI
Shape factor
(4 * π * area) / (perimeter2)
Elliptical form factor
Nuclear length/breadth

Supplemental Figure 2: Validating transmitochondrial cybrids.
(A) Plasmid map of ACE2 overexpression cassette highlights 4 PCR targets to validate A549-ACE2 nuclear specificity of ρ^+ Uk cybrids. Created in BioRender. Soto Albrecht, Y. (2026) https://BioRender.com/exka687.
(B) ACE2 transgene cassette PCR amplicons verify the nuclear identity of ρ^+ Uk cybrids derived from ρ^0 clone 11 nuclear donor. Representative of n=3.
(C) Karyotyping validates the mononuclear and A549 chromosome count of ρ^+ Uk cybrids. 3 cell replicates, n=1.
(D) Expanded OCR by Oroboros respirometry on permeabilized cells (Figure 2C) show ρ^+ Uk cybrids with replenished OXPHOS activity across all tested parameters; "FCCP" is repeat data. 2 replicates, n=5 (WT and ρ

+ cybrids).
(E) ACE2 transcript expression by qPCR graphed as fold change (2^-(ΔΔCT) of ACE2 to HPRT1 over ρ^0 clone 11. Averaged technical duplicates, 3 well replicates, n=1.
(F) – (G) Representative western blot performed on ACE2 protein (30 µg loaded) **(F)**, quantified by normalizing to loading control β-actin **(G)**. Representative of n=3.
(H) Mitochondrial mass staining (Mitotracker + DAPI counterstain) depicts mitochondrial patterns and network morphology. Representative of 3 well replicates and n=3.
(I) - (K) Quantitative measures of nuclear morphology with ImageXpress Hi.AT high content imager; 2 well replicates and representative of n=2; each dot is a nucleus (n=4,391-16,178) **(J-K)**. Representative images of nuclei acquired by confocal microscopy, n = 6+ **(I)**.
Mean + SD where applicable. Where statistics are shown, a two-way ANOVA was performed, compared to A549 **(C)**, WT A549-ACE2 **(D)**, ρ^0 **(E)**, or all samples compared to each other **(J-K)** where * = p < 0.05; ** = p < 0.01; *** = p < 0.001; **** = p < 0.0001. Image brightness adjusted simultaneously for visibility.

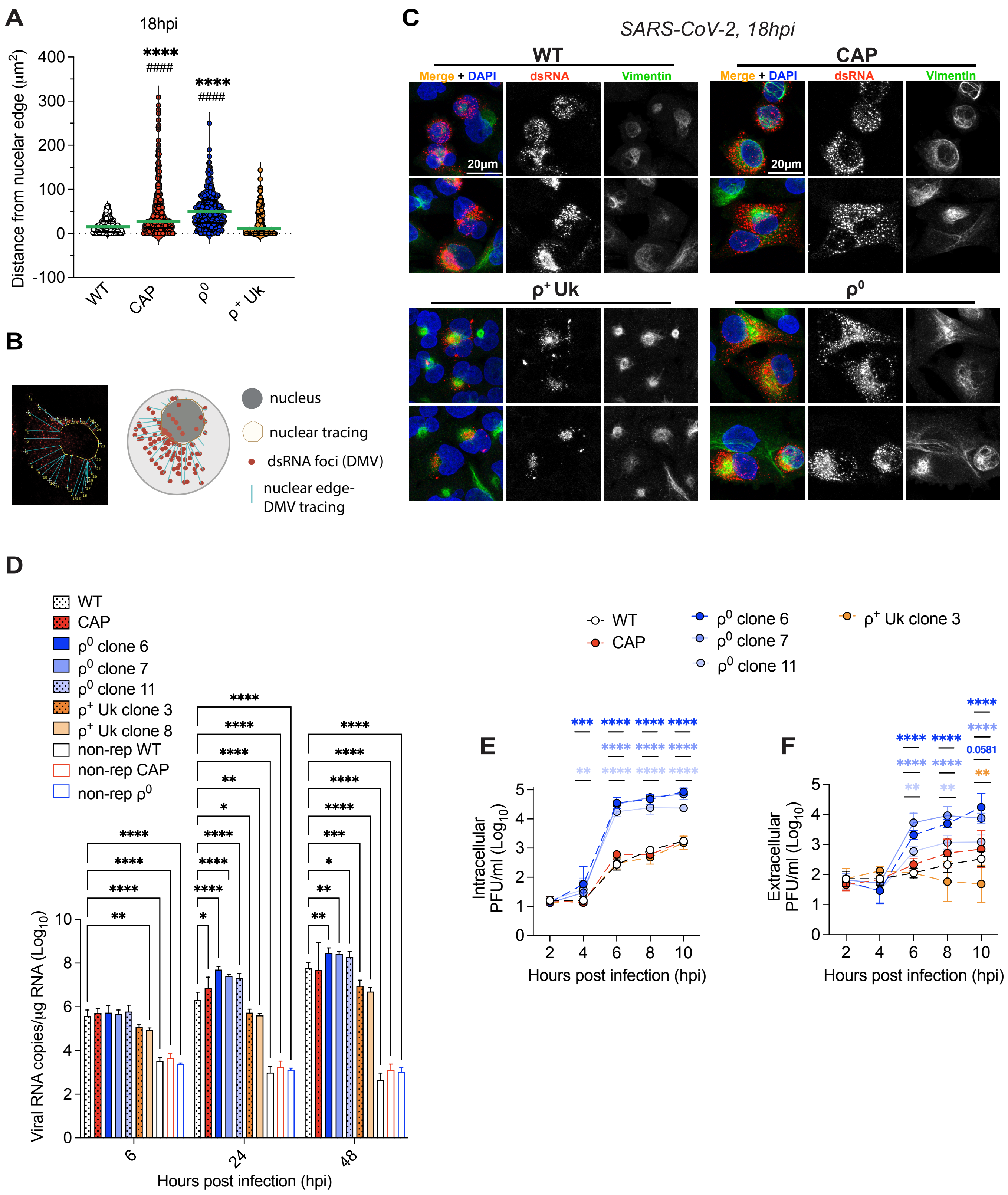

A
18hpi
Distance from nucelar edge (μm²)
WT
CAP
ρ0
ρ+ Uk
B
nucleus
nuclear tracing
dsRNA foci (DMV)
nuclear edge-DMV tracing
C
SARS-CoV-2, 18hpi
WT
CAP
Merge + DAPI
dsRNA
Vimentin
20μm
ρ+ Uk
ρ0
D
WT
CAP
ρ0 clone 6
ρ0 clone 7
ρ0 clone 11
ρ+ Uk clone 3
ρ+ Uk clone 8
non-rep WT
non-rep CAP
non-rep ρ0
Viral RNA copies/μg RNA (Log10)
Hours post infection (hpi)
E
Intracellular PFU/ml (Log10)
Hours post infection (hpi)
F
Extracellular PFU/ml (Log10)
0.0581
Hours post infection (hpi)

Supplemental Figure 3: The replicative advantage of SARS-CoV-2 in ρ^0 cells occurs prior to one full viral life cycle.

(A) – (B) In **(A)** 10 infected cells per condition from Figure 3C-E were analyzed by measuring the distance between 25 points equally distributed in the periphery of the dsRNA region and the nuclear edge, representative of n=2 analyses shown. Each dot is a distance (250 plotted per condition), and the median and quartile horizontal bars are green and black respectively and **(B)** representative tracings with nuclear edge (yellow), dsRNA (red) and distance (cyan) shown. Right panel was created in BioRender. Soto Albrecht, Y. (2026) https://BioRender.com/p38l899.

(C) SARS-CoV-2 infected cells (MOI=10) were fixed at 18hpi, stained with J2 anti-DsRNA and anti-vimentin, counterstained with Hoescht, and imaged by confocal microscopy as maximum projections. Representative images of 3+ wells, n = 2.

(D) Figure 3F expanded clones, where repeat data is shown with a dashed bar. Averaged technical duplicates, 3 well replicates, n=2.

(E) – (F) Figure 3H-I expanded clones where repeat data is shown with a dashed line. 2-4 well replicates, n = 2 of 2 (cybrids) or 3 (all other conditions) shown.

Mean + SD where applicable. Where statistics are shown, a two-way ANOVA was performed compared to WT **(D)**, WT and ρ+ Uk cells **(A)**, or 2hpi **(E-F)** where * (or #) = $p < 0.05$; ** = $p < 0.01$; *** = $p < 0.001$; **** = $p < 0.0001$. In **(A)**, * = compared to WT and # = compared to ρ^+ Uk cells. In **(E-F)**, significance is color-coded by comparison. Image brightness was adjusted simultaneously for visibility.

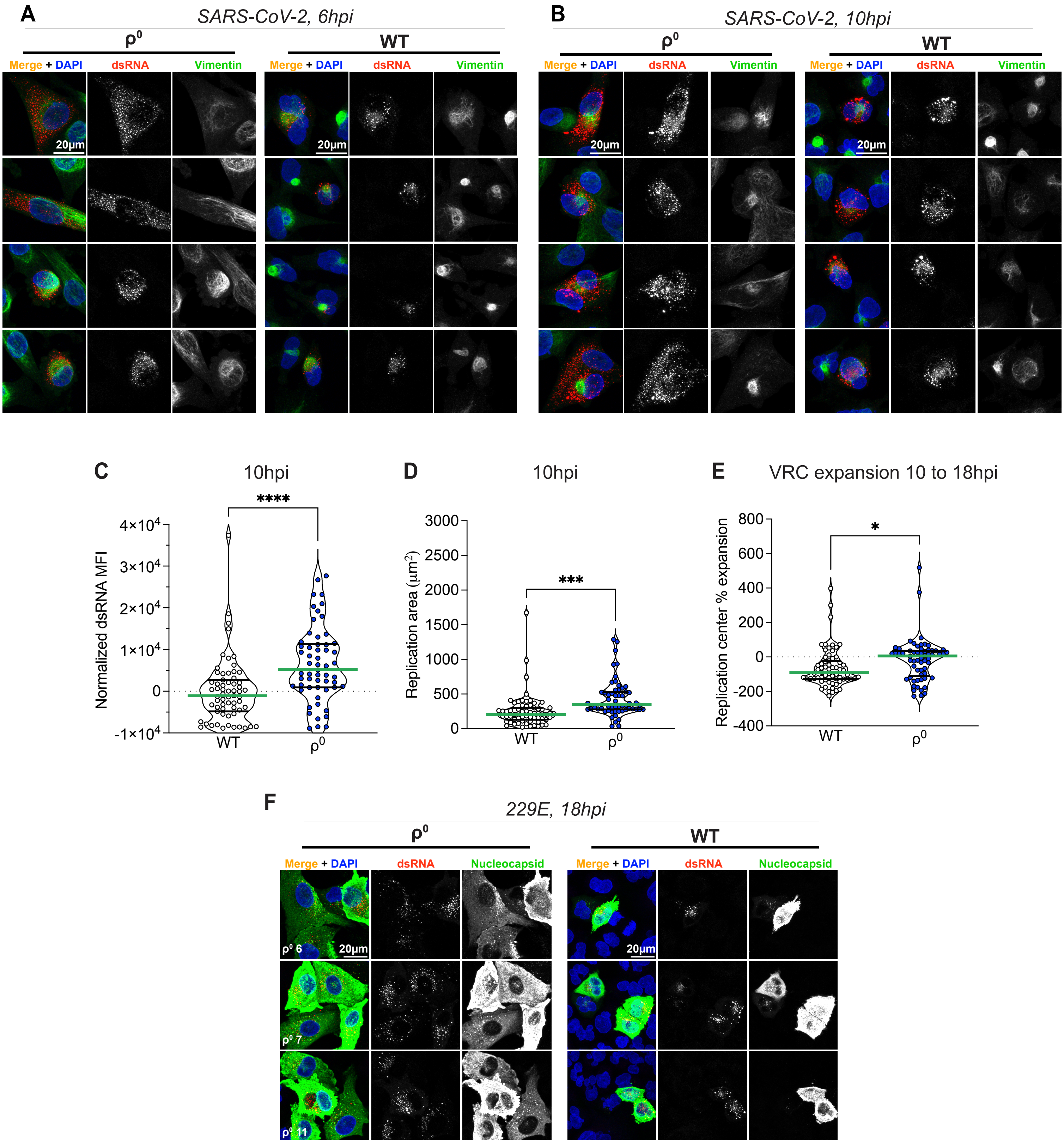
A
SARS-CoV-2, 6hpi
ρ0
WT
Merge + DAPI
dsRNA
Vimentin
20µm
B
SARS-CoV-2, 10hpi
C
10hpi
****
Normalized dsRNA MFI
4×10^4
3×10^4
2×10^4
1×10^4
0
-1×10^4
WT
ρ0
D
10hpi
***
Replication area (µm²)
3000
2500
2000
1500
1000
500
0
E
VRC expansion 10 to 18hpi
*
Replication center % expansion
800
600
400
200
0
-200
-400
F
229E, 18hpi
Nucleocapsid
ρ0 6
ρ0 7
ρ0 11

Supplemental Figure 4: The early advantage to SARS-CoV-2 replication due to OXPHOS inhibition is linked to a faster expansion of DMV area.

(A) - (B) SARS-CoV-2 infected cells (MOI=10) were fixed at 6 and 10hpi, stained with J2 anti-DsRNA and anti-vimentin, counterstained with Hoescht, and imaged by confocal microscopy as maximum projections. Representative images of 2+ wells, n=2.

(C) - (E) Quantification of SARS-CoV-2 infected cells from Figure 3D and S4D. In **(E)**, expansion of the DMV area is measured by the change area from 10 to 18hpi by subtracting 10hpi mean dsRNA area from individual 18hpi measurements in Figure 3D. 57-65 infected cells from 3+ well replicates across n=2-3 were manually analyzed from maximum projections. Each dot is a cell, where the median and quartile horizontal bars are green and black respectively. MFI is normalized by mean WT fluorescence per imaging session **(C)**.

(F) 229E infected cells (MOI=2) were fixed at 18hpi, stained with J2 anti-DsRNA and anti-nucleocapsid, counterstained with Hoescht, and imaged by confocal microscopy as maximum projections. Representative images of n=1.

Mean + SD where applicable. Where statistics are shown, a two-way ANOVA was performed compared to WT where * = $p < 0.05$; ** = $p < 0.01$; *** = $p < 0.001$; **** = $p < 0.0001$. Image brightness adjusted simultaneously for visibility.

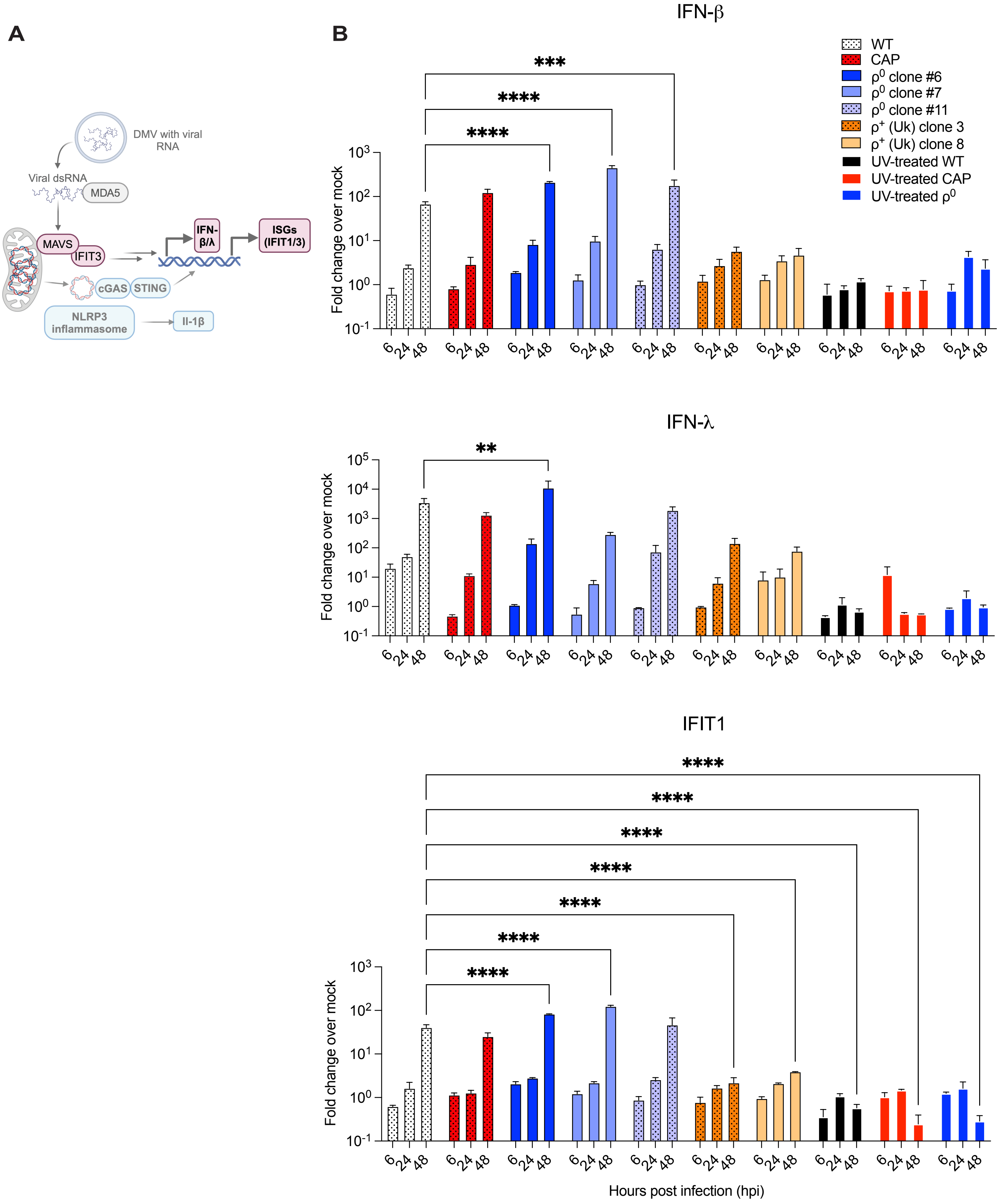
A
DMV with viral RNA
Viral dsRNA
MDA5
MAVS
IFIT3
IFN-β/λ
ISGs (IFIT1/3)
cGAS
STING
NLRP3 inflammasome
Il-1β
B
IFN-β
WT
CAP
ρ0 clone #6
ρ0 clone #7
ρ0 clone #11
ρ+ (Uk) clone 3
ρ+ (Uk) clone 8
UV-treated WT
UV-treated CAP
UV-treated ρ0
Fold change over mock
***
****
****
IFN-λ
**
IFIT1
****
Hours post infection (hpi)

Supplemental Figure 5: The interferon response to virus increases with actively replicating virus.
(A) Schematic of mitochondria as central players of the innate immune response to SARS-CoV-2 infection. Created in BioRender. Soto Albrecht, Y. (2026) https://BioRender.com/wnwgvwk.
(B) Figure 4A expanded clones where repeat data is shown with dashed bars. Non-replicating virus (Non-rep virus), 2-3 well replicates, representative of n=2.
Mean + SD where applicable. Where statistics are shown, a two-way ANOVA was performed compared to WT where ** = $p < 0.01$; *** = $p < 0.001$; **** = $p < 0.0001$.

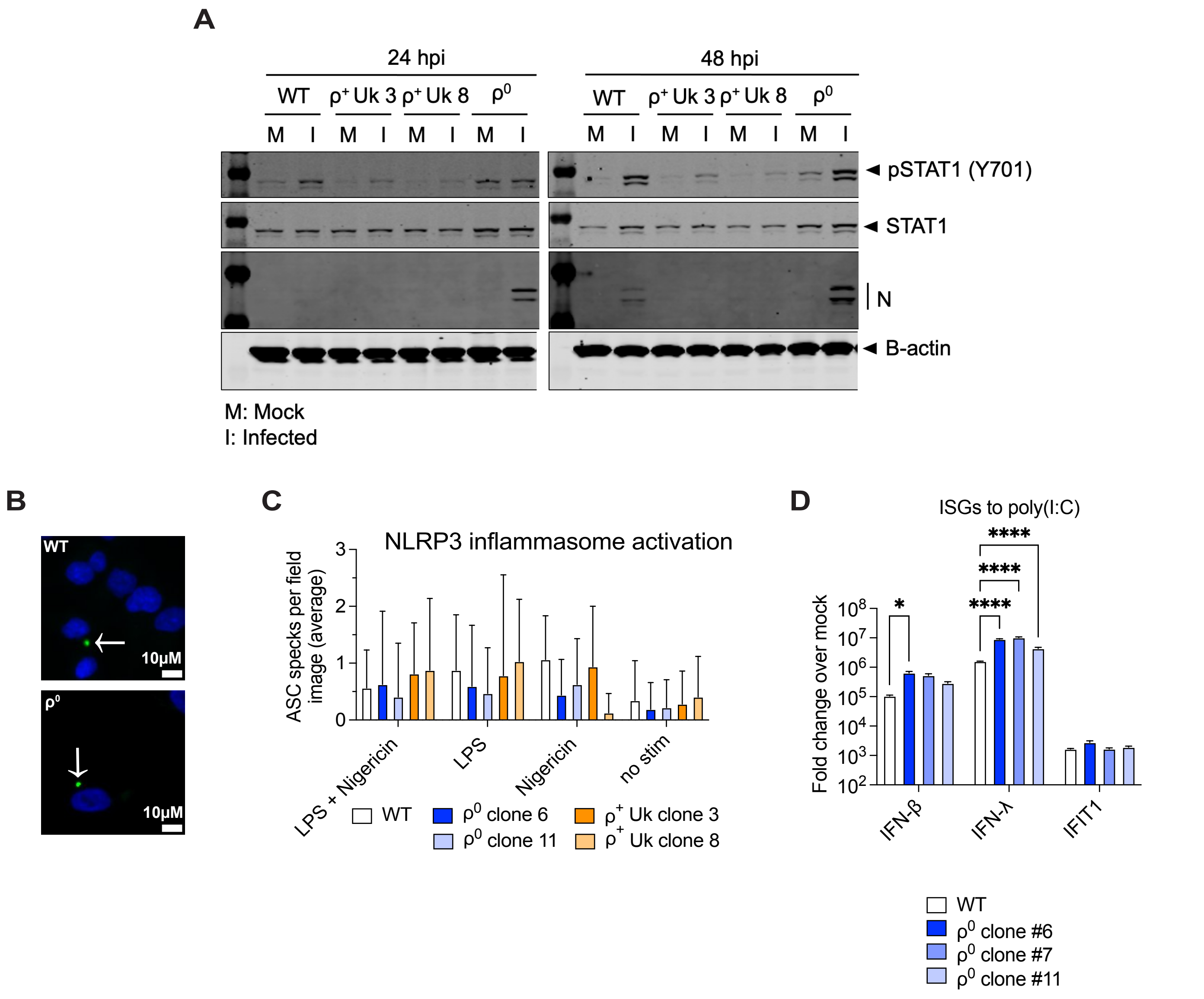

Supplemental Figure 6: The interferon pathway is initiated upstream of transcription in virally infected cells and in response to a viral RNA mimic.

(A) STAT1 and pSTAT1 (Y701) protein expression at 24hpi (left) and 48hpi (right) by western blot, with β-actin as loading control and SARS-CoV-2 N to visualize infection (10 μg loaded). Mock (M) and Infected (I) lanes are delineated. n=1.
(B) – (C) The native A549-ACE2 NLRP3 inflammasome was induced with LPS and nigericin. Cells were fixed, stained with anti-ASC speck and counterstained with Hoescht, images acquired and analyzed by Imagexpress Hi.AT high content imager. Representative images where ASC specks are highlighted with a white arrow **(B)** and quantification of ASC specks **(C)** are shown. 16-32 images from 2 wells per condition were analyzed, representative of n=3.

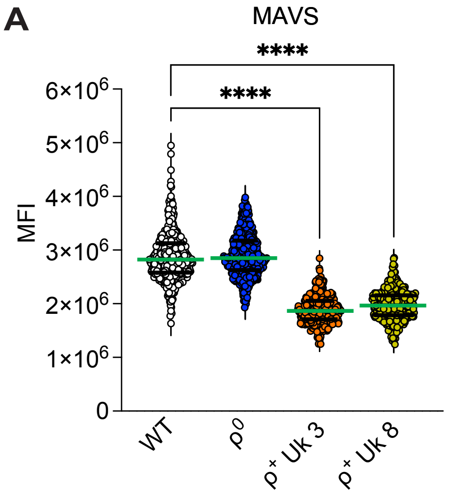

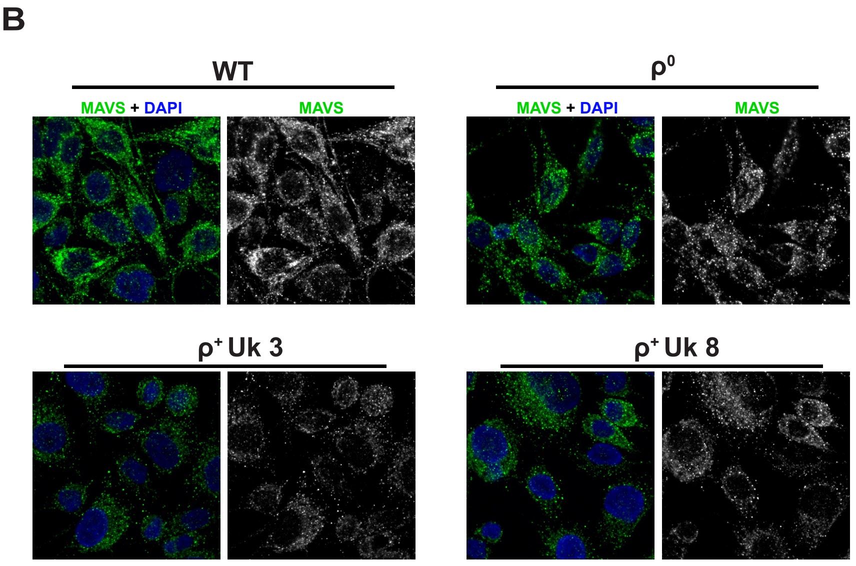

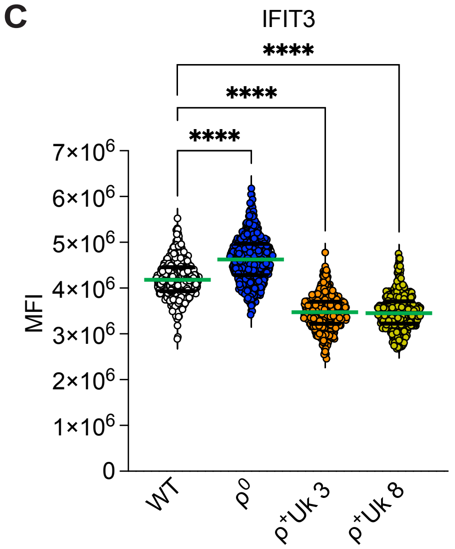

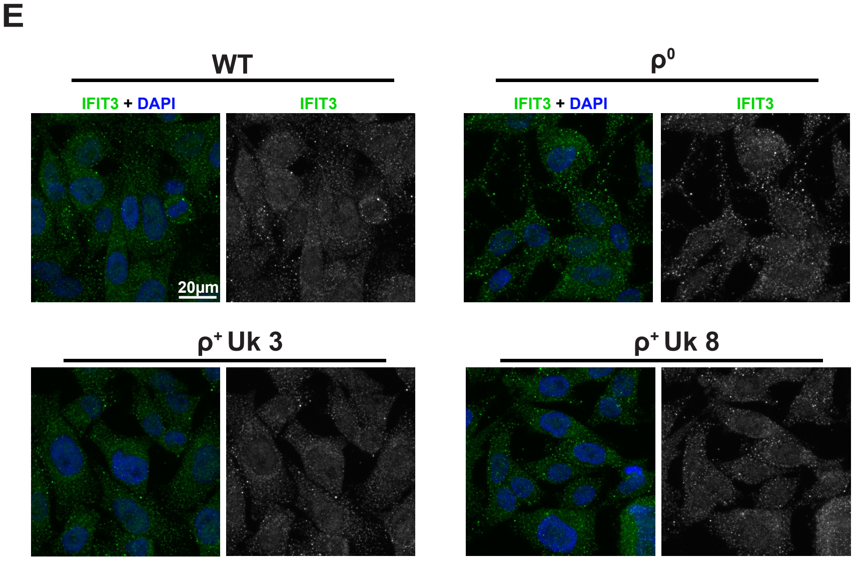

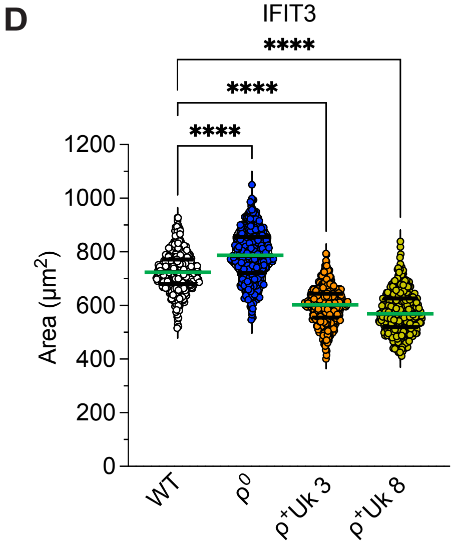

Supplemental Figure 7: Quantification of MAVS and IFIT3 in mock-infected cells mirrors infected trends.
(A) – (E) Mock-infected cells were fixed at 12hpi, stained for **(A) – (B)** MAVS and **(C) – (E)** IFIT3 respectively, counterstained with Hoechst and imaged with the ImageXpress Micro Confocal as maximum projections. Violin plots where the median and quartile horizonal bars are represented in green and black respectively depict **(A)**, **(C)** MFI and **(D)** area. An ordinary one-way ANOVA was performed where **** = $p < 0.0001$. **(B)** and **(E)** Representative images where image brightness is adjusted simultaneously for visibility. 49 images per well, 10 well replicates, n=1.

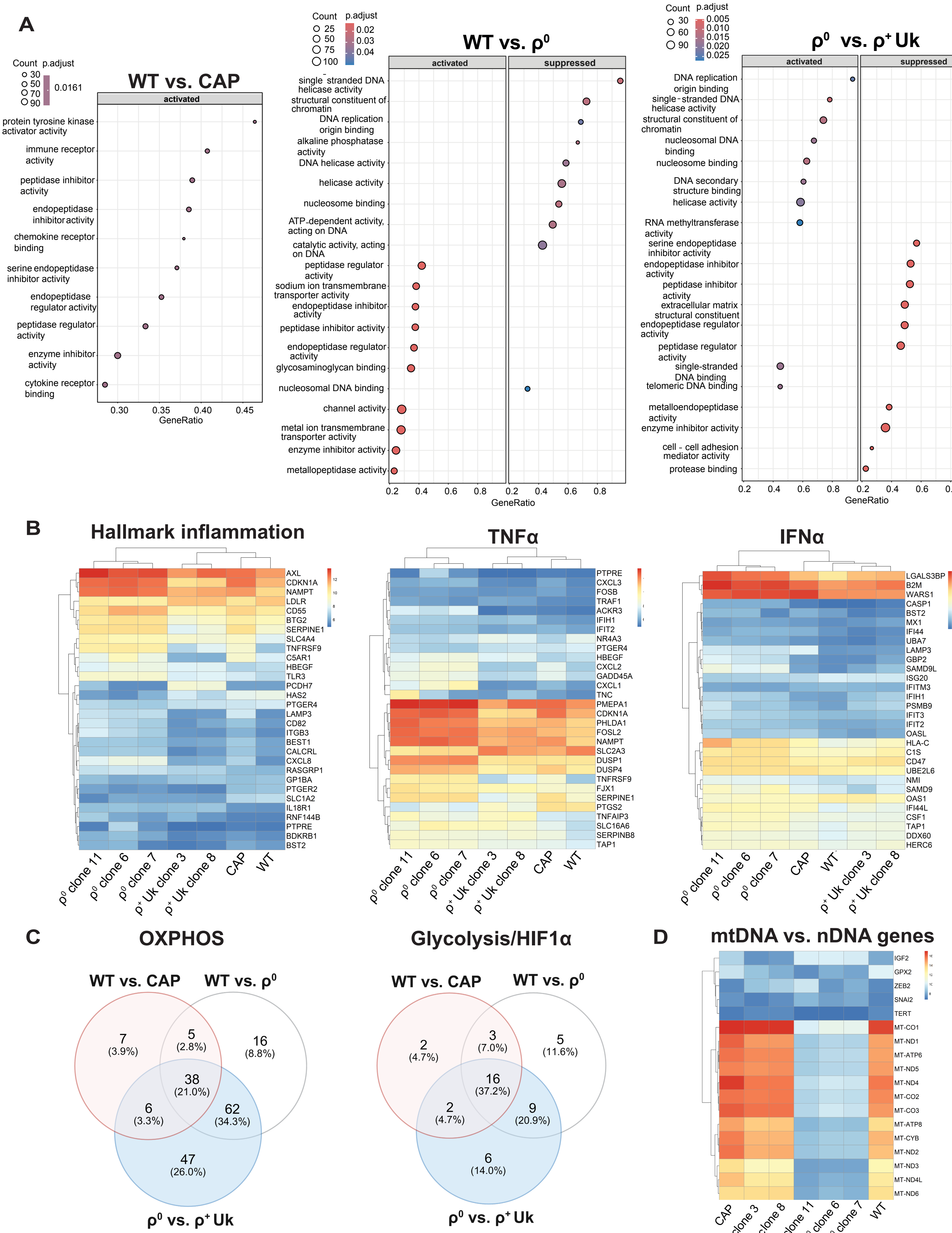

Supplemental Figure 8: OXPHOS inhibition broadly affects the transcriptional landscape, while bioenergetic changes are highlighted.

Supplemental Figure 8: OXPHOS inhibition broadly affects the transcriptional landscape, while bioenergetic changes are highlighted.
(A) – (D) Pertain to the bulk RNA-seq dataset in Figure 5.
(A) Gene Ontology Molecular Function across defined comparisons where dot size depicts gene count, X axis location the gene ratio, and color (where relevant) the p adjusted value.
(B) Pathway heatmaps depict the most differentially expressed genes from specified GSEA hallmark gene lists by log2 fold-change; note: no significance cutoff.
(C) Venn diagrams of DEGs from gene lists in Figure 5C compared to WT. Created in BioRender. Chilkatowsky, A. (2026) https://BioRender.com/cihcnje and https://biorender.com/1kgclat.
(D) A heatmap depicts the relative expression of the 13 mtDNA along with 5 nDNA OXPHOS genes across conditions.

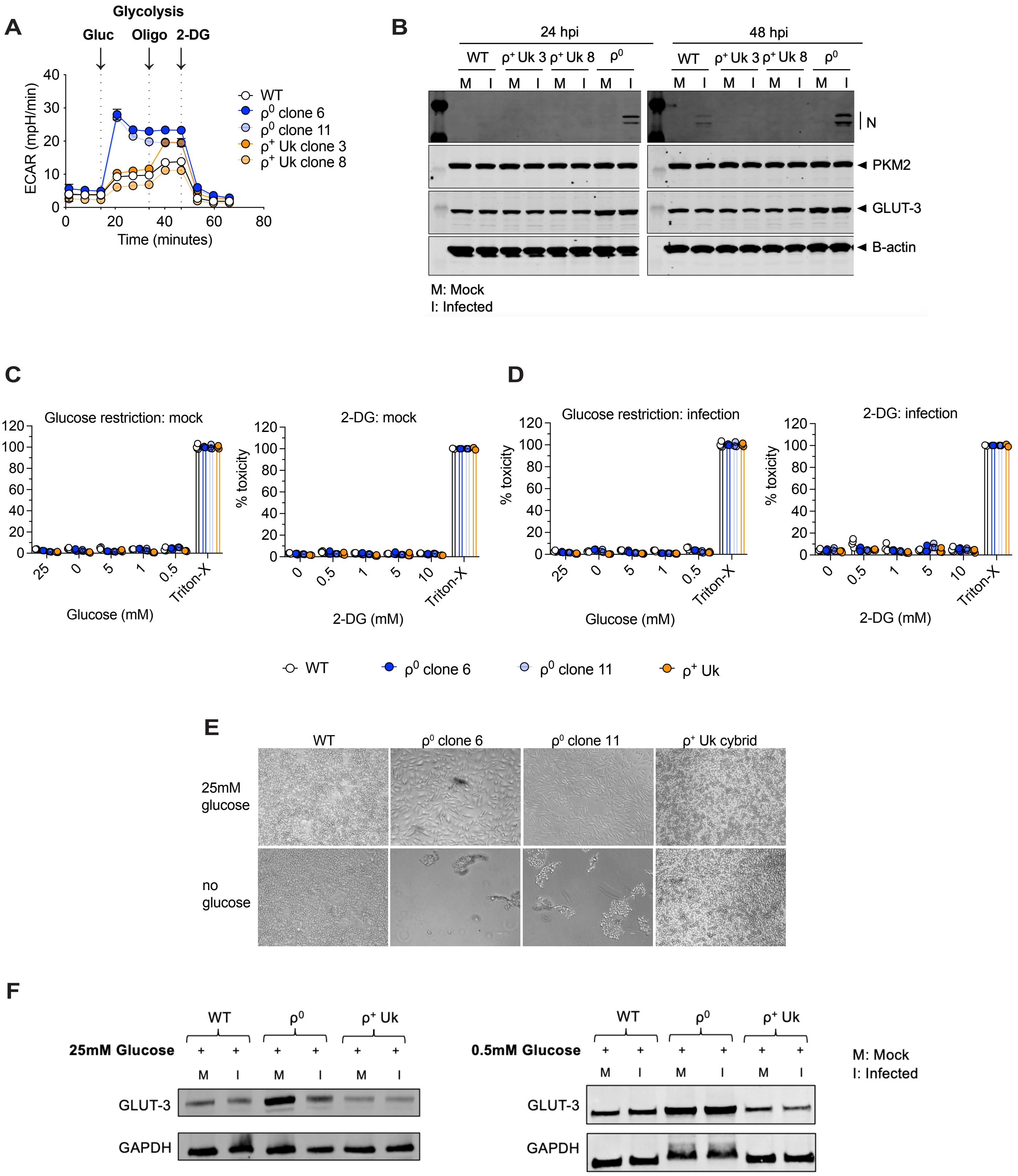
A
Glycolysis
Gluc
Oligo
2-DG
ECAR (mpH/min)
Time (minutes)
WT
ρ0 clone 6
ρ0 clone 11
ρ+ Uk clone 3
ρ+ Uk clone 8
B
24 hpi
48 hpi
WT
ρ+ Uk 3
ρ+ Uk 8
ρ0
M I
N
PKM2
GLUT-3
B-actin
M: Mock
I: Infected
C
Glucose restriction: mock
2-DG: mock
% toxicity
Glucose (mM)
2-DG (mM)
Triton-X
D
Glucose restriction: infection
2-DG: infection
WT
ρ0 clone 6
ρ0 clone 11
ρ+ Uk
E
WT
ρ0 clone 6
ρ0 clone 11
ρ+ Uk cybrid
25mM glucose
no glucose
F
25mM Glucose
0.5mM Glucose
WT
ρ0
ρ+ Uk
GLUT-3
GAPDH
M: Mock
I: Infected

Supplemental Figure 9: OXPHOS inhibition causes upregulation of glycolytic activity and proteins critical for cell survival.
(A) Representative ECAR tracing of Figure 5D. Representative of 10 well replicates, n=3.
(B) PKM2 and GLUT-3 protein expression at 24hpi (left) and 48hpi (right) by western blot, with β-actin as loading control and SARS-CoV-2 N to visualize infection (10 μg loaded). β-actin and N lanes re-used from Figure S6A. Mock (M) and Infected (I) lanes are delineated. n=1.
(C) – (D) LDH cytotoxicity for **(D)** mock-infected and **(E)** infected cells from Figure 6A-B, validated an additional n=2 times (n=5 total).
(E) Brightfield images of cells with or without glucose for 12 hours demonstrate the toxicity of glucose deprivation when OXPHOS is inhibited. Representative of n=2.
(F) GLUT-3 protein expression in 12hpi cell pellets from Figure 6B at 25mM (left) and 0.5mM (right) glucose conditions with GAPDH as loading control (20 μg loaded). Mock (M) and Infected (I) lanes are delineated. n=1.

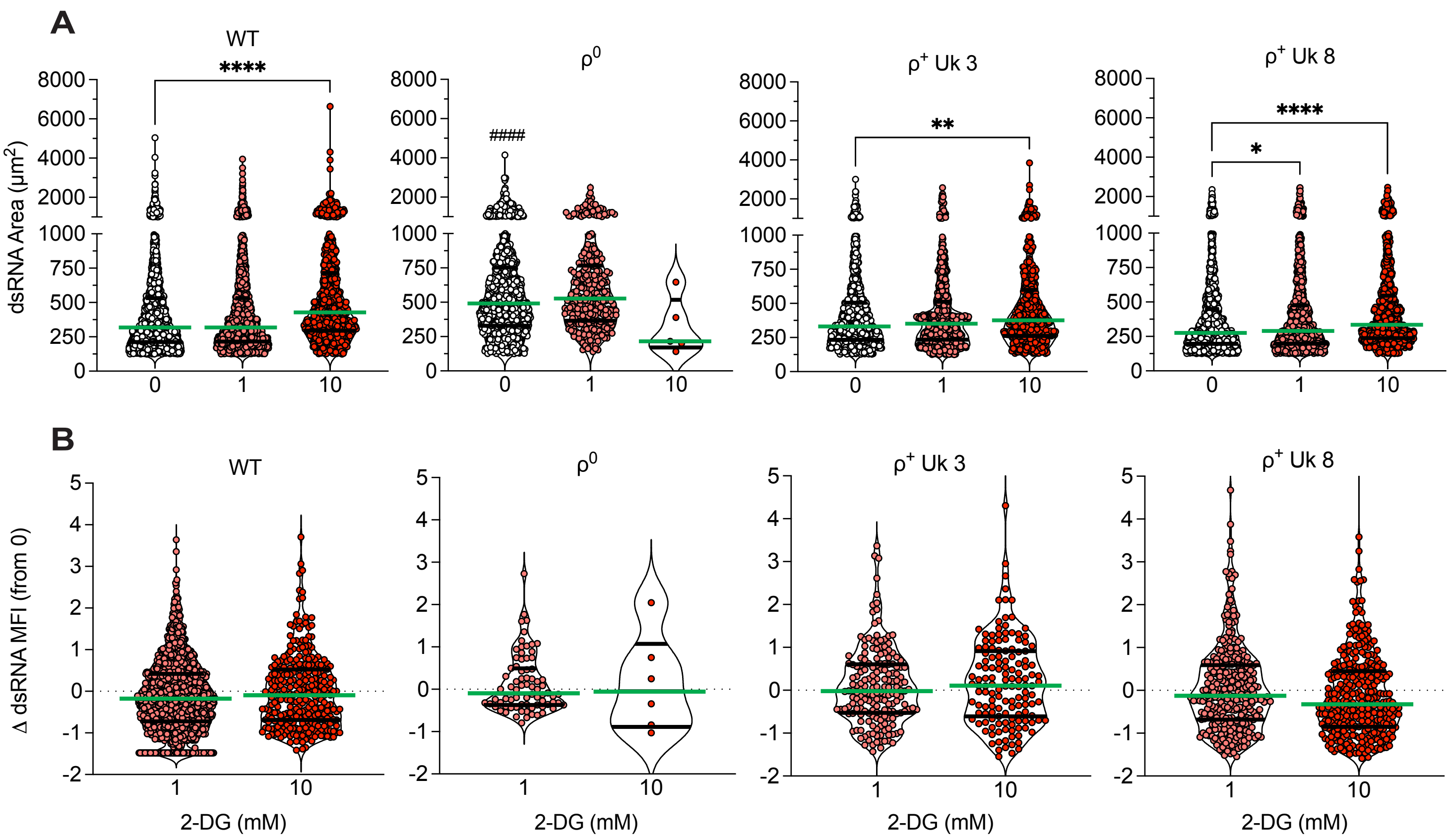

Supplemental Figure 10: Glycolysis activity regulates intracellular viral replication center distribution in infected cells.

(A) – (B) Expanded quantitative analysis of Figure 6 **(C) – (D)**. dsRNA area and MFI from dsRNA-positive cells were calculated and plotted as **(A)** dsRNA area or **(B)** change in MFI from the 0mM 2-DG (control) group. The median and quartile horizontal bars are green and black, respectively. The dotted line in **(B)** represents 0. An ordinary one-way ANOVA was performed compared to the control group (iterations of *) or different cell types treated with 0mM 2-DG compared to ρ^0 0mM 2-DG treated cells (####). Specifically, * = $p < 0.05$; ** = $p < 0.01$; *** = $p < 0.001$; **** or #### = $p < 0.0001$.

TABLE LEGENDS: SUPPLEMENTAL

Table S1: mtDNA sequences of A549-ACE2 endogenous mtDNA and ρ^+ Uk mtDNAs from 143B(TK^-) parental and A459-ACE2 transmitochondrial cybrids.

The entire 16.5 kbp mtDNA was PCR-amplified, assembled, and mtDNA haplogroups defined using MITOMAP.Org (tab 1), and potentially pathologic mtDNA variants in the A549 and Uk mtDNA genomes explored (tab 2 and 3) along with associated heteroplasmy (tab 4).

Table S2: Bulk RNAseq gene ontology plots demonstrate that dozens of pathways are regulated by OXPHOS function in A549-ACE2 cells.

Pertains to the bulk RNA-seq dataset in **Figure 5.** Gene Ontology analysis of Molecular Function, Biological Process, and Cellular Components reveal dramatic differences in cellular membranous structures, mitochondrial metabolism, and inflammation in addition to the emphasized differences in bioenergetics.

Table S3: Bulk RNAseq Venn diagrams and volcano plot gene lists.

Pertains to the bulk RNA-seq dataset in **Figure 5C.**

Table S4: Bulk RNAseq complete DEGs of interest.

Pertains to the bulk RNA-seq dataset in **Figure 5.**

Table S5: Antibodies.

Antibodies and related information.

REFERENCES

1. World Health Organization, COVID-19 Dashboard, https://covid19.who.int/.

2. A. Gupta, M. V. Madhavan, K. Sehgal, N. Nair, S. Mahajan, T. S. Sehrawat, B. Bikdeli, N. Ahluwalia, J. C. Ausiello, E. Y. Wan, D. E. Freedberg, A. J. Kirtane, S. A. Parikh, M. S. Maurer, A. S. Nordvig, D. Accili, J. M. Bathon, S. Mohan, K. A. Bauer, M. B. Leon, H. M. Krumholz, N. Uriel, M. R. Mehra, M. S. V. Elkind, G. W. Stone, A. Schwartz, D. D. Ho, J. P. Bilezikian, D. W. Landry, Extrapulmonary manifestations of COVID-19. *Nat. Med.* **26**, 1017–1032 (2020).

3. A. M. Carabelli, T. P. Peacock, L. G. Thorne, W. T. Harvey, J. Hughes, COVID-19 Genomics UK Consortium, S. J. Peacock, W. S. Barclay, T. I. de Silva, G. J. Towers, D. L. Robertson, SARS-CoV-2 variant biology: Immune escape, transmission and fitness. *Nat. Rev. Microbiol.* **21**, 162–177 (2023).

4. R. F. Service, Bad news for Paxlovid? Coronavirus can find multiple ways to evade COVID-19 drug. *Science* **377**, 138–139 (2022).

5. J. F.-W. Chan, K. H. Kok, Z. Zhu, H. Chu, K. K.-W. To, S. Yuan, K.-Y. Yuen, Genomic characterization of the 2019 novel human-pathogenic coronavirus isolated from a patient with atypical pneumonia after visiting Wuhan. *Emerg. Microbes Infect.* **9**, 221–236 (2020).

6. P. V'kovski, A. Kratzel, S. Steiner, H. Stalder, V. Thiel, Coronavirus biology and replication: Implications for SARS-CoV-2. *Nat. Rev. Microbiol.* **19**, 155–170 (2021).

7. A. R. Fehr, S. Perlman, Coronaviruses: An overview of their replication and pathogenesis. *Methods Mol. Biol.* **1282**, 1–23 (2015).

8. D. Wat, The common cold: A review of the literature. *Eur. J. Intern. Med.* **15**, 79–88 (2004).

9. E. R. Gaunt, A. Hardie, E. C. Claas, P. Simmonds, K. E. Templeton, Epidemiology and clinical presentations of the four human coronaviruses 229E, HKU1, NL63, and OC43 detected over 3 years using a novel multiplex real-time PCR method. *J. Clin. Microbiol.* **48**, 2940–2947 (2010).

10. J. Hurtado-Tamayo, R. Requena-Platek, L. Enjuanes, M. Bello-Perez, I. Sola, Contribution to pathogenesis of accessory proteins of deadly human coronaviruses. *Front. Cell. Infect. Microbiol.* **13**, 1166839 (2023).

11. K. Knoops, M. Kikkert, S. H. E. van den Worm, J. C. Zevenhoven-Dobbe, Y. van der Meer, A. J. Koster, A. M. Mommaas, E. J. Snijder, SARS-coronavirus replication is supported by a reticulovesicular network of modified endoplasmic reticulum. *PLOS Biol.* **6**, e226 (2008).

12. G. Wolff, R. W. A. L. Limpens, J. C. Zevenhoven-Dobbe, U. Laugks, S. Zheng, A. W. M. de Jong, R. I. Koning, D. A. Agard, K. Grünewald, A. J. Koster, E. J. Snijder, M. Bárcena, A molecular pore spans the double membrane of the coronavirus replication organelle. *Science* **369**, 1395–1398 (2020).

13. M. Cortese, J. Y. Lee, B. Cerikan, C. J. Neufeldt, V. M. J. Oorschot, S. Köhrer, J. Hennies, N. L. Schieber, P. Ronchi, G. Mizzon, I. Romero-Brey, R. Santarella-Mellwig, M. Schorb, M. Boermel, K. Mocaer, M. S. Beckwith, R. M. Templin, V. Gross, C. Pape, C. Tischer, J. Frankish, N. K. Horvat, V. Laketa, M. Stanifer, S. Boulant, A. Ruggieri, L. Chatel-Chaix, Y. Schwab, R. Bartenschlager, Integrative imaging reveals SARS-CoV-2-induced reshaping of subcellular morphologies. *Cell Host Microbe* **28**, 853–866.e5 (2020).

14. J. J. A. van Kampen, D. A. M. C. van de Vijver, P. L. A. Fraaij, B. L. Haagmans, M. M. Lamers, N. Okba, J. P. C. van den Akker, H. Endeman, D. A. M. P. J. Gommers, J. J. Cornelissen, R. A. S. Hoek, M. M. van der Eerden, D. A. Hesselink, H. J. Metselaar, A. Verbon, J. E. M. de Steenwinkel, G. I. Aron, E. C. M. van Gorp, S. van Boheemen, J. C. Voermans, C. A. B. Boucher, R. Molenkamp, M. P. G. Koopmans, C. Geurtsvankessel, A. A. van der Eijk, Duration and key determinants of infectious virus shedding in hospitalized patients with coronavirus disease-2019 (COVID-19). *Nat. Commun.* **12**, 267 (2021).

15. J. Fajnzylber, J. Regan, K. Coxen, H. Corry, C. Wong, A. Rosenthal, D. Worrall, F. Giguel, A. Piechocka-Trocha, C. Atyeo, S. Fischinger, A. Chan, K. T. Flaherty, K. Hall, M. Dougan, E. T. Ryan, E. Gillespie, R. Chishti, Y. Li, N. Jilg, D. Hanidziar, R. M. Baron, L. Baden, A. M. Tsibris, K. A. Armstrong, D. R. Kuritzkes, G. Alter, B. D. Walker, X. Yu, J. Z. Li, Massachusetts Consortium for Pathogen Readiness, SARS-CoV-2 viral load is associated with increased disease severity and mortality. *Nat. Commun.* **11**, 5493 (2020).

16. M. E. Soria, M. Cortón, B. Martínez-González, R. Lobo-Vega, L. Vázquez-Sirvent, R. López-Rodríguez, B. Almoguera, I. Mahillo, P. Mínguez, A. Herrero, J. C. Taracido, A. Macías-Valcayo, J. Esteban, R. Fernandez-Roblas, I. Gadea, J. Ruíz-Hornillos, C. Ayuso, C. Perales, High SARS-CoV-2 viral load is associated with a worse clinical outcome of COVID-19 disease. *Access Microbiol.* **3**, 000259 (2021).

17. A. M. Abdel-Haleem, N. E. Lewis, N. Jamshidi, K. Mineta, X. Gao, T. Gojobori, The emerging facets of non-cancerous Warburg effect. *Front. Endocrinol.* **8**, 279 (2017).

18. H. Myllymäki, L. Kelly, A. M. Elliot, R. N. Carter, J. A. Johansson, K. Y. Chang, J. Cholewa-Waclaw, N. M. Morton, Y. Feng, Preneoplastic cells switch to Warburg metabolism from their inception exposing multiple vulnerabilities for targeted elimination. *Oncogenesis* **13**, 2 (2024).

19. A. S. Naidu, C.-K. Wang, P. Rao, F. Mancini, R. A. Clemens, A. Wirakartakusumah, H.-F. Chiu, C.-H. Yen, S. Porretta, I. Mathai, S. A. G. Naidu, Precision nutrition to reset virus-induced human metabolic reprogramming and dysregulation (HMRD) in long-COVID. *NPJ Sci. Food* **8**, 19 (2024).

20. S. K. Thaker, J. Ch'ng, H. R. Christofk, Viral hijacking of cellular metabolism. *BMC Biol.* **17**, 59 (2019).

21. J. W. Guarnieri, J. M. Dybas, H. Fazelinia, M. S. Kim, J. Frere, Y. Zhang, Y. Soto Albrecht, D. G. Murdock, A. Angelin, L. N. Singh, S. L. Weiss, S. M. Best, M. T. Lott, S. Zhang, H. Cope, V. Zaksas, A. Saravia-Butler, C. Meydan, J. Foox, C. Mozsary, Y. Bram, Y. Kidane, W. Priebe, M. R. Emmett, R. Meller, S. Demharter, V. Stentoft-Hansen, M. Salvatore, D. Galeano, F. J. Enguita, P. Grabham, N. S. Trovao, U. Singh, J. Haltom, M. T. Heise, N. J. Moorman, V. K. Baxter, E. A. Madden, S. A. Taft-Benz, E. J. Anderson, W. A. Sanders, R. J. Dickmander, S. B. Baylin, E. S. Wurtele, P. M. Moraes-Vieira, D. Taylor, C. E. Mason, J. C. Schisler, R. E. Schwartz, A. Beheshti, D. C. Wallace, Core mitochondrial genes are down-regulated during SARS-CoV-2 infection of rodent and human hosts. *Sci. Transl. Med.* **15**, eabq1533 (2023).

22. B. Miller, A. Silverstein, M. Flores, K. Cao, H. Kumagai, H. H. Mehta, K. Yen, S.-J. Kim, P. Cohen, Host mitochondrial transcriptome response to SARS-CoV-2 in multiple cell models and clinical samples. *Sci. Rep.* **11**, 3 (2021).

23. D. Bojkova, R. Costa, P. Reus, M. Bechtel, M.-C. Jaboreck, R. Olmer, U. Martin, S. Ciesek, M. Michaelis, J. Cinatl Jr., Targeting the pentose phosphate pathway for SARS-CoV-2 therapy. *Metabolites* **11**, 699 (2021).

24. C. Shang, Z. Liu, Y. Zhu, J. Lu, C. Ge, C. Zhang, N. Li, N. Jin, Y. Li, M. Tian, X. Li, SARS-CoV-2 causes mitochondrial dysfunction and mitophagy impairment. *Front. Microbiol.* **12**, 780768 (2021).

25. P. J. Mullen, G. Garcia, A. Purkayastha, N. Matulionis, E. W. Schmid, M. Momcilovic, C. Sen, J. Langerman, A. Ramaiah, D. B. Shackelford, R. Damoiseaux, S. W. French, K. Plath, B. N. Gomperts, V. Arumugaswami, H. R. Christofk, SARS-CoV-2 infection rewires host cell metabolism and is potentially susceptible to mTORC1 inhibition. *Nat. Commun.* **12**, 1876 (2021).

26. M. W. Gray, Mosaic nature of the mitochondrial proteome: Implications for the origin and evolution of mitochondria. *Proc. Natl. Acad. Sci. U.S.A.* **112**, 10133–10138 (2015).

27. H. J. Balbi, Chloramphenicol: A review. *Pediatr. Rev.* **25**, 284–288 (2004).

28. M. Giacomello, A. Pyakurel, C. Glytsou, L. Scorrano, The cell biology of mitochondrial membrane dynamics. *Nat. Rev. Mol. Cell Biol.* **21**, 204–224 (2020).

29. J. F. Turrens, Mitochondrial formation of reactive oxygen species. *J. Physiol.* **552**, 335–344 (2003).

30. A. J. Majmundar, W. J. Wong, M. C. Simon, Hypoxia-inducible factors and the response to hypoxic stress. *Mol. Cell* **40**, 294–309 (2010).

31. A. M. Nargund, M. W. Pellegrino, C. J. Fiorese, B. M. Baker, C. M. Haynes, Mitochondrial import efficiency of ATFS-1 regulates mitochondrial UPR activation. *Science* **337**, 587–590 (2012).

32. E. Fessler, E. M. Eckl, S. Schmitt, I. A. Mancilla, M. F. Meyer-Bender, M. Hanf, J. Philippou-Massier, S. Krebs, H. Zischka, L. T. Jae, A pathway coordinated by DELE1 relays mitochondrial stress to the cytosol. *Nature* **579**, 433–437 (2020).

33. J. W. Guarnieri, T. Lie, Y. E. S. Albrecht, P. Hewin, K. A. Jurado, G. A. Widjaja, Y. Zhu, M. J. McManus, T. J. Kilbaugh, K. Keith, P. Potluri, D. Taylor, A. Angelin, D. G. Murdock, D. C. Wallace, Mitochondrial antioxidants abate SARS-COV-2 pathology in mice. *Proc. Natl. Acad. Sci. U.S.A.* **121**, e2321972121 (2024).

34. M. Tian, W. Liu, X. Li, P. Zhao, M. A. Shereen, C. Zhu, S. Huang, S. Liu, X. Yu, M. Yue, P. Pan, W. Wang, Y. Li, X. Chen, K. Wu, Z. Luo, Q. Zhang, J. Wu, HIF-1α promotes SARS-CoV-2 infection and aggravates inflammatory responses to COVID-19. *Signal Transduct. Target. Ther.* **6**, 308 (2021).

35. A. C. Codo, G. G. Davanzo, L. de Brito Monteiro, G. F. de Souza, S. P. Muraro, J. V. Virgilio-da-Silva, J. S. Prodonoff, V. C. Carregari, C. A. O. de Biagi Junior, F. Crunfli, J. L. Jimenez Restrepo, P. H. Vendramini, G. Reis-de-Oliveira, K. B. Dos Santos, D. A. Toledo-Teixeira, P. L. Parise, M. C. Martini, R. E. Marques, H. R. Carmo, A. Borin, L. D. Coimbra, V. O. Boldrini, N. S. Brunetti, A. S. Vieira, E. Mansour, R. G. Ulaf, A. F. Bernardes, T. A. Nunes, L. C. Ribeiro, A. C. Palma, M. V. Agrela, M. L. Moretti, A. C. Sposito, F. B. Pereira, L. A. Velloso, M. A. Ramirez Vinolo, A. Damasio, J. L. Proença-Módena, R. F. Carvalho, M. A. Mori, D. Martins-de-Souza, H. I. Nakaya, A. S. Farias, P. M. Moraes-Vieira, Elevated glucose levels favor SARS-CoV-2 infection and monocyte response through a HIF-1α/glycolysis-dependent axis. *Cell Metab.* **32**, 437–446.e5 (2020).

36. D. Bojkova, K. Klann, B. Koch, M. Widera, D. Krause, S. Ciesek, J. Cinatl, C. Münch, Proteomics of SARS-CoV-2-infected host cells reveals therapy targets. *Nature* **583**, 469–472 (2020).

37. A. N. Bhatt, A. Kumar, Y. Rai, N. Kumari, D. Vedagiri, K. H. Harshan, V. Chinnadurai, S. Chandna, Glycolytic inhibitor 2-deoxy-D-glucose attenuates SARS-CoV-2 multiplication in host cells and weakens the infective potential of progeny virions. *Life Sci.* **295**, 120411 (2022).

38. F. Gabanella, C. Barbato, N. Corbi, M. Fiore, C. Petrella, M. de Vincentiis, A. Greco, G. Ferraguti, A. Corsi, M. Ralli, I. Pecorella, C. Di Gioia, F. Pecorini, R. Brunelli, C. Passananti, A. Minni, M. G. Di Certo, Exploring mitochondrial localization of SARS-CoV-2 RNA by padlock assay: A pilot study in human placenta. *Int. J. Mol. Sci.* **23**, 2100 (2022).

39. K. E. Wu, F. M. Fazal, K. R. Parker, J. Zou, H. Y. Chang, RNA-GPS predicts SARS-CoV-2 RNA residency to host mitochondria and nucleolus. *Cell Syst.* **11**, 102–108.e3 (2020).

40. K. K. Singh, G. Chaubey, J. Y. Chen, P. Suravajhala, Decoding SARS-CoV-2 hijacking of host mitochondria in COVID-19 pathogenesis. *Am. J. Physiol. Cell Physiol.* **319**, C258–C267 (2020).

41. Z. Cheng, D. Zhang, J. Chen, Y. Wu, X. Liu, L. Si, Z. Zhang, N. Zhang, Z. Zhang, W. Liu, H. Liu, L. Zhang, L. Song, L. S. C. Dunmall, J. Dong, N. R. Lemoine, Y. Wang, A novel viral protein translation mechanism reveals mitochondria as a target for antiviral drug development. bioRxiv 2020.10.19.344713 [Preprint] (2020). https://doi.org/10.1101/2020.10.19.344713.

42. R. Dwivedi, SARS-CoV-2 hijacks mitochondria, https://news-medical.net/news/20201021/SARS-CoV-2-hijacks-mitochondria.aspx.

43. C. Wu, Y. Liu, Y. Yang, P. Zhang, W. Zhong, Y. Wang, Q. Wang, Y. Xu, M. Li, X. Li, M. Zheng, L. Chen, H. Li, Analysis of therapeutic targets for SARS-CoV-2 and discovery of potential drugs by computational methods. *Acta Pharm. Sin. B* **10**, 766–788 (2020).

44. X. Zheng, L. Li, Potential therapeutic options for COVID-19. *Infect. Microbes Dis.* **2**, 89–95 (2020).

45. G. Tao, W. Liao, J. Hou, X. Jiang, X. Deng, G. Chen, C. Ding, Advances in crosstalk among innate immune pathways activated by mitochondrial DNA. *Heliyon* **10**, e24029 (2024).

46. J. Yoon, S. Kim, M. Lee, Y. Kim, Mitochondrial nucleic acids in innate immunity and beyond. *Exp. Mol. Med.* **55**, 2508–2518 (2023).

47. K. Nakahira, J. A. Haspel, V. A. Rathinam, S. J. Lee, T. Dolinay, H. C. Lam, J. A. Englert, M. Rabinovitch, M. Cernadas, H. P. Kim, K. A. Fitzgerald, S. W. Ryter, A. M. Choi, Autophagy proteins regulate innate immune responses by inhibiting the release of mitochondrial DNA mediated by the NALP3 inflammasome. *Nat. Immunol.* **12**, 222–230 (2011).

48. K. Shimada, T. R. Crother, J. Karlin, J. Dagvadorj, N. Chiba, S. Chen, V. K. Ramanujan, A. J. Wolf, L. Vergnes, D. M. Ojcius, A. Rentsendorj, M. Vargas, C. Guerrero, Y. Wang, K. A. Fitzgerald, D. M. Underhill, T. Town, M. Arditi, Oxidized mitochondrial DNA activates the NLRP3 inflammasome during apoptosis. *Immunity* **36**, 401–414 (2012).

49. Y. Wu, C. Hao, X. Liu, G. Han, J. Yin, Z. Zou, J. Zhou, C. Xu, MitoQ protects against liver injury induced by severe burn plus delayed resuscitation by suppressing the mtDNA-NLRP3 axis. *Int. Immunopharmacol.* **80**, 106189 (2020).

50. A. Vanderheiden, P. Ralfs, T. Chirkova, A. A. Upadhyay, M. G. Zimmerman, S. Bedoya, H. Aoued, G. M. Tharp, K. L. Pellegrini, C. Manfredi, E. Sorscher, B. Mainou, J. L. Lobby, J. E. Kohlmeier, A. C. Lowen, P. Y. Shi, V. D. Menachery, L. J. Anderson, A. Grakoui, S. E. Bosinger, M. S. Suthar, Type I and type III interferons restrict SARS-CoV-2 infection of human airway epithelial cultures. *J. Virol.* **94**, e00985-20 (2020).

51. M. S. Diamond, T.-D. Kanneganti, Innate immunity: The first line of defense against SARS-CoV-2. *Nat. Immunol.* **23**, 165–176 (2022).

52. A. C. Ferreira, V. C. Soares, I. G. de Azevedo-Quintanilha, S. da Silva Gomes Dias, N. Fintelman-Rodrigues, C. Q. Sacramento, M. Mattos, C. S. de Freitas, J. R. Temerozo, L. Teixeira, E. D. Hottz, E. A. Barreto, C. R. R. Pão, L. Palhinha, M. Miranda, D. C. Bou-Habib, F. A. Bozza, P. T. Bozza, T. M. L. Souza, SARS-CoV-2 engages inflammasome and pyroptosis in human primary monocytes. *Cell Death Discov.* **7**, 179 (2021).

53. P. Pan, M. Shen, Z. Yu, W. Ge, K. Chen, M. Tian, F. Xiao, Z. Wang, J. Wang, Y. Jia, W. Wang, P. Wan, J. Zhang, W. Chen, Z. Lei, X. Chen, Z. Luo, Q. Zhang, M. Xu, G. Li, Y. Li, J. Wu, SARS-CoV-2 N protein promotes NLRP3 inflammasome activation to induce hyperinflammation. *Nat. Commun.* **12**, 4664 (2021).

54. T. S. Rodrigues, K. S. G. de Sá, A. Y. Ishimoto, A. Becerra, S. Oliveira, L. Almeida, A. V. Gonçalves, D. B. Perucello, W. A. Andrade, R. Castro, F. P. Veras, J. E. Toller-Kawahisa, D. C. Nascimento, M. H. F. de Lima, C. M. S. Silva, D. B. Caetite, R. B. Martins, I. A. Castro, M. C. Pontelli, F. C. de Barros, N. B. do Amaral, M. C. Giannini, L. P. Bonjorno, M. I. F. Lopes, R. C. Santana, F. C. Vilar, M. Auxiliadora-Martins, R. Luppino-Assad, S. C. L. de Almeida, F. R. de Oliveira, S. S. Batah, L. Siyuan, M. N. Benatti, T. M. Cunha, J. C. Alves-Filho, F. Q. Cunha, L. D. Cunha, F. G. Frantz, T. Kohlsdorf, A. T. Fabro, E. Arruda, R. D. R. de Oliveira, P. Louzada-Junior, D. S. Zamboni, Inflammasomes are activated in response to SARS-CoV-2 infection and are associated with COVID-19 severity in patients. *J. Exp. Med.* **218**, e20201707 (2021).

55. J. W. Guarnieri, A. Angelin, D. G. Murdock, P. Schaefer, P. Portluri, T. Lie, J. Huang, D. C. Wallace, SARS-COV-2 viroporins activate the NLRP3-inflammasome by the mitochondrial permeability transition pore. *Front. Immunol.* **14**, 1064293 (2023).

56. M. Li, M. Ferretti, B. Ying, H. Descamps, E. Lee, M. Dittmar, J. S. Lee, K. Whig, B. Kamalia, L. Dohnalová, G. Uhr, H. Zarkoob, Y.-C. Chen, H. Ramage, M. Ferrer, K. Lynch, D. C. Schultz, C. A. Thaiss, M. S. Diamond, S. Cherry, Pharmacological activation of STING blocks SARS-CoV-2 infection. *Sci. Immunol.* **6**, eabi9007 (2021).

57. F. Humphries, L. Shmuel-Galia, Z. Jiang, R. Wilson, P. Landis, S.-L. Ng, K. M. Parsi, R. Maehr, J. Cruz, A. Morales-Ramos, J. M. Ramanjulu, J. Bertin, G. S. Pesiridis, K. A. Fitzgerald, A diamidobenzimidazole STING agonist protects against SARS-CoV-2 infection. *Sci. Immunol.* **6**, eabi9002 (2021).

58. X. Yin, L. Riva, Y. Pu, L. Martin-Sancho, J. Kanamune, Y. Yamamoto, K. Sakai, S. Gotoh, L. Miorin, P. D. De Jesus, C. C. Yang, K. M. Herbert, S. Yoh, J. F. Hultquist, A. García-Sastre, S. K. Chanda, MDA5 governs the innate immune response to SARS-CoV-2 in lung epithelial cells. *Cell Rep.* **34**, 108628 (2021).

59. A. G. Bodnar, J. M. Cooper, I. J. Holt, J. V. Leonard, A. H. Schapira, Nuclear complementation restores mtDNA levels in cultured cells from a patient with mtDNA depletion. *Am. J. Hum. Genet.* **53**, 663–669 (1993).

60. D. R. Dunbar, P. A. Moonie, H. T. Jacobs, I. J. Holt, Different cellular backgrounds confer a marked advantage to either mutant or wild-type mitochondrial genomes. *Proc. Natl. Acad. Sci. U.S.A.* **92**, 6562–6566 (1995).

61. R. Mineri, N. Pavelka, E. Fernandez-Vizarra, P. Ricciardi-Castagnoli, M. Zeviani, V. Tiranti, How do human cells react to the absence of mitochondrial DNA. *PLOS ONE* **4**, e5713 (2009).

62. R. F. Hoffmann, M. R. Jonker, S. M. Brandenburg, H. G. de Bruin, N. H. T. Ten Hacken, A. J. M. van Oosterhout, I. H. Heijink, Mitochondrial dysfunction increases pro-inflammatory cytokine production and impairs repair and corticosteroid responsiveness in lung epithelium. *Sci. Rep.* **9**, 15047 (2019).

63. Y. Gao, P. Dorn, S. Liu, H. Deng, S. R. R. Hall, R. W. Peng, R. A. Schmid, T. M. Marti, Cisplatin-resistant A549 non-small cell lung cancer cells can be identified by increased mitochondrial mass and are sensitive to pemetrexed treatment. *Cancer Cell Int.* **19**, 317 (2019).

64. J. Yang, L. Liu, Y. Oda, K. Wada, M. Ago, S. Matsuda, M. Hattori, T. Goto, Y. Kawashima, Y. Matsuzaki, T. Taketani, Highly-purified rapidly expanding clones, RECs, are superior for functional-mitochondrial transfer. *Stem Cell Res. Ther.* **14**, 40 (2023).

65. M. Han, E. A. Bushong, M. Segawa, A. Tiard, A. Wong, M. R. Brady, M. Momcilovic, D. M. Wolf, R. Zhang, A. Petcherski, M. Madany, S. Xu, J. T. Lee, M. V. Poyurovsky, K. Olszewski, T. Holloway, A. Gomez, M. S. John, S. M. Dubinett, C. M. Koehler, O. S. Shirihai, L. Stiles, A. Lisberg, S. Soatto, S. Sadeghi, M. H. Ellisman, D. B. Shackelford, Spatial mapping of mitochondrial networks and bioenergetics in lung cancer. *Nature* **615**, 712–719 (2023).

66. C. A. Schmidt, K. H. Fisher-Wellman, P. D. Neufer, From OCR and ECAR to energy: Perspectives on the design and interpretation of bioenergetics studies. *J. Biol. Chem.* **297**, 101140 (2021).

67. M. Strauss, G. Hofhaus, R. R. Schröder, W. Kühlbrandt, Dimer ribbons of ATP synthase shape the inner mitochondrial membrane. *EMBO J.* **27**, 1154–1160 (2008).

68. X. Xie, A. Muruato, K. G. Lokugamage, K. Narayanan, X. Zhang, J. Zou, J. Liu, C. Schindewolf, N. E. Bopp, P. V. Aguilar, K. S. Plante, S. C. Weaver, S. Makino, J. W. LeDuc, V. D. Menachery, P.-Y. Shi, An infectious cDNA clone of SARS-CoV-2. *Cell Host Microbe* **27**, 841–848.e3 (2020).

69. A. Kukat, C. Kukat, J. Brocher, I. Schäfer, G. Krohne, I. A. Trounce, G. Villani, P. Seibel, Generation of ρ^0 cells utilizing a mitochondrially targeted restriction endonuclease and comparative analyses. *Nucleic Acids Res.* **36**, e44 (2008).

70. J. Annunen-Rasila, S. Ohlmeier, H. Tuokko, J. Veijola, K. Majamaa, Proteome and cytoskeleton responses in osteosarcoma cells with reduced OXPHOS activity. *Proteomics* **7**, 2189–2200 (2007).

71. I. Aretz, C. Hardt, I. Wittig, D. Meierhofer, An impaired respiratory electron chain triggers down-regulation of the energy metabolism and de-ubiquitination of solute carrier amino acid transporters. *Mol. Cell. Proteomics* **15**, 1526–1538 (2016).

72. X. Wang, Y. Zhao, F. Yan, T. Wang, W. Sun, N. Feng, W. Wang, H. Wang, H. He, S. Yang, X. Xia, Y. Gao, Viral and host transcriptomes in SARS-CoV-2-infected human lung cells. *J. Virol.* **95**, e0060021 (2021).

73. C. A. Higgins, B. E. Nilsson-Payant, B. Bonaventure, A. P. Kurland, C. Ye, T. M. Yaron, J. L. Johnson, P. Adhikary, I. Golynker, M. Panis, O. Danziger, B. R. Rosenberg, L. C. Cantley, L. Martínez-Sobrido, B. tenOever, J. R. Johnson, SARS-CoV-2 hijacks p38β/MAPK11 to promote virus replication. *mBio* **14**, e0100723 (2023).

74. A. F. Bradburne, An investigation of the replication of coronaviruses in suspension cultures of L132 cells. *Arch. Gesamte Virusforsch.* **37**, 297–307 (1972).

75. M. Schneider, K. Ackermann, M. Stuart, C. Wex, U. Protzer, H. M. Schätzl, S. Gilch, Severe acute respiratory syndrome coronavirus replication is severely impaired by MG132 due to proteasome-independent inhibition of M-calpain. *J. Virol.* **86**, 10112–10122 (2012).

76. Y. Zhang, R. Guo, S. H. Kim, H. Shah, S. Zhang, J. H. Liang, Y. Fang, M. Gentili, C. N. O. Leary, S. J. Elledge, D. T. Hung, V. K. Mootha, B. E. Gewurz, SARS-CoV-2 hijacks folate and one-carbon metabolism for viral replication. *Nat. Commun.* **12**, 1676 (2021).

77. Y. Li, D. M. Renner, C. E. Comar, J. N. Whelan, H. M. Reyes, F. L. Cardenas-Diaz, R. Truitt, L. H. Tan, B. Dong, K. D. Alysandratos, J. Huang, J. N. Palmer, N. D. Adappa, M. A. Kohanski, D. N. Kotton, R. H. Silverman, W. Yang, E. E. Morrisey, N. A. Cohen, S. R. Weiss, SARS-CoV-2 induces double-stranded RNA-mediated innate immune responses in respiratory epithelial-derived cells and cardiomyocytes. *Proc. Natl. Acad. Sci. U.S.A.* **118**, e2022643118 (2021).

78. M. A. Suter, N. Y. Tan, C. H. Thiam, M. Khatoo, P. A. MacAry, V. Angeli, S. Gasser, Y. L. Zhang, cGAS-STING cytosolic DNA sensing pathway is suppressed by JAK2-STAT3 in tumor cells. *Sci. Rep.* **11**, 7243 (2021).

79. A. Stutz, G. L. Horvath, B. G. Monks, E. Latz, ASC speck formation as a readout for inflammasome activation. *Methods Mol. Biol.* **1040**, 91–101 (2013).

80. K. Abe, S. Ikeda, M. Nara, A. Kitadate, H. Tagawa, N. Takahashi, Hypoxia-induced oxidative stress promotes therapy resistance via upregulation of heme oxygenase-1 in multiple myeloma. *Cancer Med.* **12**, 9709–9722 (2023).

81. J. Ji, F. Bi, X. Zhang, Z. Zhang, Y. Xie, Q. Yang, Single-cell transcriptome analysis revealed heterogeneity in glycolysis and identified IGF2 as a therapeutic target for ovarian cancer subtypes. *BMC Cancer* **24**, 926 (2024).

82. S. Qie, N. Sang, Stanniocalcin 2 (STC2): A universal tumour biomarker and a potential therapeutical target. *J. Exp. Clin. Cancer Res.* **41**, 161 (2022).

83. S. Polo-Generelo, C. Rodríguez-Mateo, B. Torres, J. Pintor-Tortolero, J. A. Guerrero-Martínez, J. König, J. Vázquez, E. Bonzón-Kulichenco, J. Padillo-Ruiz, F. de la Portilla, J. C. Reyes, J. A. Pintor-Toro, *Serpine1* mRNA confers mesenchymal characteristics to the cell and promotes CD8+ T cells exclusion from colon adenocarcinomas. *Cell Death Discov.* **10**, 116 (2024).

84. J. Krishnan, M. Suter, R. Windak, T. Krebs, A. Felley, C. Montessuit, M. Tokarska-Schlattner, E. Aasum, A. Bogdanova, E. Perriard, J.-C. Perriard, T. Larsen, T. Pedrazzini, W. Krek, Activation of a HIF1α-PPARγ axis underlies the integration of glycolytic and lipid anabolic pathways in pathologic cardiac hypertrophy. *Cell Metab.* **9**, 512–524 (2009).

85. S. Srivastava, F. Diaz, L. Iommarini, K. Aure, A. Lombes, C. T. Moraes, PGC-1α/β induced expression partially compensates for respiratory chain defects in cells from patients with mitochondrial disorders. *Hum. Mol. Genet.* **18**, 1805–1812 (2009).

86. J. E. Toller-Kawahisa, C. H. Hiroki, C. M. de Souza Silva, D. C. Nascimento, G. A. Públio, T. V. Martins, L. E. A. Damasceno, F. P. Veras, P. R. Viacava, F. Y. Sukesada, E. A. Day, A. Zotta, T. A. J. Ryan, R. M. da Silva, T. M. Cunha, N. P. Lopes, F. de Queiroz Cunha, L. A. J. O'Neill, J. C. Alves-Filho, The metabolic function of pyruvate kinase M2 regulates reactive oxygen species production and microbial killing by neutrophils. *Nat. Commun.* **14**, 4280 (2023).

87. S. M. Hochrein, H. Wu, M. Eckstein, L. Arrigoni, J. S. Herman, F. Schumacher, C. Gerecke, M. Rosenfeldt, D. Grün, B. Kleuser, G. Gasteiger, W. Kastenmüller, B. Ghesquière, J. Van den Bossche, E. D. Abel, M. Vaeth, The glucose transporter GLUT3 controls T helper 17 cell responses through glycolytic-epigenetic reprogramming. *Cell Metab.* **34**, 516–532.e11 (2022).

88. P. K. Kopinski, L. N. Singh, S. Zhang, M. T. Lott, D. C. Wallace, Mitochondrial DNA variation and cancer. *Nat. Rev. Cancer* **21**, 431–445 (2021).

89. D. C. Wallace, Mitochondria and cancer. *Nat. Rev. Cancer* **12**, 685–698 (2012).

90. P. Wang, R. Luo, M. Zhang, Y. Wang, T. Song, T. Tao, Z. Li, L. Jin, H. Zheng, W. Chen, M. Zhao, Y. Zheng, J. Qin, A cross-talk between epithelium and endothelium mediates human alveolar-capillary injury during SARS-CoV-2 infection. *Cell Death Dis.* **11**, 1042 (2020).

91. R. A. Flynn, J. A. Belk, Y. Qi, Y. Yasumoto, J. Wei, M. M. Alfajaro, Q. Shi, M. R. Mumbach, A. Limaye, P. C. DeWeirdt, C. O. Schmitz, K. R. Parker, E. Woo, H. Y. Chang, T. L. Horvath, J. E. Carette, C. R. Bertozzi, C. B. Wilen, A. T. Satpathy, Discovery and functional interrogation of SARS-CoV-2 RNA-host protein interactions. *Cell* **184**, 2394–2411.e16 (2021).

92. Y. K. Yoon, H. P. Kim, S. W. Han, D. Y. Oh, S. A. Im, Y. J. Bang, T. Y. Kim, KRAS mutant lung cancer cells are differentially responsive to MEK inhibitor due to AKT or STAT3 activation: Implication for combinatorial approach. *Mol. Carcinog.* **49**, 353–362 (2010).

93. Y. Hu, W. Lu, G. Chen, P. Wang, Z. Chen, Y. Zhou, M. Ogasawara, D. Trachootham, L. Feng, H. Pelicano, P. J. Chiao, M. J. Keating, G. Garcia-Manero, P. Huang, K-ras(G12V) transformation leads to mitochondrial dysfunction and a metabolic switch from oxidative phosphorylation to glycolysis. *Cell Res.* **22**, 399–412 (2012).

94. A. Nasonovs, M. Garcia-Diaz, D. F. Bogenhagen, A549 cells contain enlarged mitochondria with independently functional clustered mtDNA nucleoids. *PLOS ONE* **16**, e0249047 (2021).

95. G. Cannino, R. El-Khoury, M. Pirinen, B. Hutz, P. Rustin, H. T. Jacobs, E. Dufour, Glucose modulates respiratory complex I activity in response to acute mitochondrial dysfunction. *J. Biol. Chem.* **287**, 38729–38740 (2012).

96. Z. Wen, Y. Zhang, Z. Lin, K. Shi, Y. Jiu, Cytoskeleton—A crucial key in host cell for coronavirus infection. *J. Mol. Cell Biol.* **12**, 968–979 (2020).

97. X. Zhang, H. Shi, J. Chen, D. Shi, H. Dong, L. Feng, Identification of the interaction between vimentin and nucleocapsid protein of transmissible gastroenteritis virus. *Virus Res.* **200**, 56–63 (2015).

98. C. M. Harrison, J. M. Doster, E. H. Landwehr, N. P. Kumar, E. J. White, D. C. Beachboard, C. C. Stobart, Evaluating the virology and evolution of seasonal human coronaviruses associated with the common cold in the COVID-19 era. *Microorganisms* **11**, 445 (2023).

99. D. E. Gordon, G. M. Jang, M. Bouhaddou, J. Xu, K. Obernier, K. M. White, M. J. O'Meara, V. V. Rezelj, J. Z. Guo, D. L. Swaney, T. A. Tummino, R. Hüttenhain, R. M. Kaake, A. L. Richards, B. Tutuncuoglu, H. Foussard, J. Batra, K. Haas, M. Modak, M. Kim, P. Haas, B. J. Polacco, H. Braberg, J. M. Fabius, M. Eckhardt, M. Soucheray, M. J. Bennett, M. Cakir, M. J. McGregor, Q. Li, B. Meyer, F. Roesch, T. Vallet, A. MacKain, L. Miorin, E. Moreno, Z. Z. C. Naing, Y. Zhou, S. Peng, Y. Shi, Z. Zhang, W. Shen, I. T. Kirby, J. E. Melnyk, J. S. Chorba, K. Lou, S. A. Dai, I. Barrio-Hernandez, D. Memon, C. Hernandez-Armenta, J. Lyu, C. J. P. Mathy, T. Perica, K. B. Pilla, S. J. Ganesan, D. J. Saltzberg, R. Rakesh, X. Liu, S. B. Rosenthal, L. Calviello, S. Venkataramanan, J. Liboy-Lugo, Y. Lin, X.-P. Huang, Y. Liu, S. A. Wankowicz, M. Bohn, M. Safari, F. S. Ugur, C. Koh, N. S. Savar, Q. D. Tran, D. Shengjuler, S. J. Fletcher, M. C. O'Neal, Y. Cai, J. C. J. Chang, D. J. Broadhurst, S. Klippsten, P. P. Sharp, N. A. Wenzell, D. Kuzuoglu-Ozturk, H.-Y. Wang, R. Trenker, J. M. Young, D. A. Cavero, J. Hiatt, T. L. Roth, U. Rathore, A. Subramanian, J. Noack, M. Hubert, R. M. Stroud, A. D. Frankel, O. S. Rosenberg, K. A. Verba, D. A. Agard, M. Ott, M. Emerman, N. Jura, M. von Zastrow, E. Verdin, A. Ashworth, O. Schwartz, C. d'Enfert, S. Mukherjee, M. Jacobson, H. S. Malik, D. G. Fujimori, T. Ideker, C. S. Craik, S. N. Floor, J. S. Fraser, J. D. Gross, A. Sali, B. L. Roth, D. Ruggero, J. Taunton, T. Kortemme, P. Beltrao, M. Vignuzzi, A. García-Sastre, K. M. Shokat, B. K. Shoichet, N. J. Krogan, A SARS-CoV-2 protein interaction map reveals targets for drug repurposing. *Nature* **583**, 459–468 (2020).

100. D. E. Gordon, J. Hiatt, M. Bouhaddou, V. V. Rezelj, S. Ulferts, H. Braberg, A. S. Jureka, K. Obernier, J. Z. Guo, J. Batra, R. M. Kaake, A. R. Weckstein, T. W. Owens, M. Gupta, S. Pourmal, E. W. Titus, M. Cakir, M. Soucheray, M. McGregor, Z. Cakir, G. Jang, M. J. O'Meara, T. A. Tummino, Z. Zhang, H. Foussard, A. Rojc, Y. Zhou, D. Kuchenov, R. Hüttenhain, J. Xu, M. Eckhardt, D. L. Swaney, J. M. Fabius, M. Ummadi, B. Tutuncuoglu, U. Rathore, M. Modak, P. Haas, K. M. Haas, Z. Z. C. Naing, E. H. Pulido, Y. Shi, I. Barrio-Hernandez, D. Memon, E. Petsalaki, A. Dunham, M. C. Marrero, D. Burke, C. Koh, T. Vallet, J. A. Silvas, C. M. Azumaya, C. Billesbølle, A. F. Brilot, M. G. Campbell, A. Diallo, M. S. Dickinson, D. Diwanji, N. Herrera, N. Hoppe, H. T. Kratochvil, Y. Liu, G. E. Merz,

M. Moritz, H. C. Nguyen, C. Nowotny, C. Puchades, A. N. Rizo, U. Schulze-Gahmen, A. M. Smith, M. Sun, I. D. Young, J. Zhao, D. Asarnow, J. Biel, A. Bowen, J. R. Braxton, J. Chen, C. M. Chio, U. S. Chio, I. Deshpande, L. Doan, B. Faust, S. Flores, M. Jin, K. Kim, V. L. Lam, F. Li, J. Li, Y. L. Li, Y. Li, X. Liu, M. Lo, K. E. Lopez, A. A. Melo, F. R. Moss, P. Nguyen, J. Paulino, K. I. Pawar, J. K. Peters, T. H. Pospiech, M. Safari, S. Sangwan, K. Schaefer, P. V. Thomas, A. C. Thwin, R. Trenker, E. Tse, T. K. M. Tsui, F. Wang, N. Whitis, Z. Yu, K. Zhang, Y. Zhang, F. Zhou, D. Saltzberg, QCRG Structural Biology Consortium, A. J. Hodder, A. S. Shun-Shion, D. M. Williams, K. M. White, R. Rosales, T. Kehrer, L. Miorin, E. Moreno, A. H. Patel, S. Rihn, M. M. Khalid, A. Vallejo-Gracia, P. Fozouni, C. R. Simoneau, T. L. Roth, D. Wu, M. A. Karim, M. Ghoussaini, I. Dunham, F. Berardi, S. Weigang, M. Chazal, J. Park, J. Logue, M. McGrath, S. Weston, R. Haupt, C. J. Hastie, M. Elliott, F. Brown, K. A. Burness, E. Reid, M. Dorward, C. Johnson, S. G. Wilkinson, A. Geyer, D. M. Giesel, C. Baillie, S. Raggett, H. Leech, R. Toth, N. Goodman, K. C. Keough, A. L. Lind, C. Zoonomia, R. J. Klesh, K. R. Hemphill, J. Carlson-Stevermer, J. Oki, K. Holden, T. Maures, K. S. Pollard, A. Sali, D. A. Agard, Y. Cheng, J. S. Fraser, A. Frost, N. Jura, T. Kortemme, A. Manglik, D. R. Southworth, R. M. Stroud, D. R. Alessi, P. Davies, M. B. Frieman, T. Ideker, C. Abate, N. Jouvenet, G. Kochs, B. Shoichet, M. Ott, M. Palmarini, K. M. Shokat, A. García-Sastre, J. A. Rassen, R. Grosse, O. S. Rosenberg, K. A. Verba, C. F. Basler, M. Vignuzzi, A. A. Peden, P. Beltrao, N. J. Krogan, Comparative host-coronavirus protein interaction networks reveal pan-viral disease mechanisms. *Science* **370**, eabe9403 (2020).

101. M. I. Faizan, R. Chaudhuri, S. Sagar, S. Albogami, N. Chaudhary, I. Azmi, A. Akhtar, S. M. Ali, R. Kumar, J. Iqbal, M. C. Joshi, G. Kharya, P. Seth, S. S. Roy, T. Ahmad, NSP4 and ORF9b of SARS-CoV-2 induce pro-inflammatory mitochondrial dna release in inner membrane-derived vesicles. *Cells* **11**, 2969 (2022).

102. P. Fang, L. Fang, H. Zhang, S. Xia, S. Xiao, Functions of coronavirus accessory proteins: Overview of the state of the art. *Viruses* **13**, 1139 (2021).

103. D. Vázquez-Coto, G. M. Albaiceta, L. Amado-Rodríguez, M. G. Clemente, E. Cuesta-Llavona, J. Gómez, E. Coto, Common mitochondrial haplogroups as modifiers of the onset-age for critical COVID-19. *Mitochondrion* **67**, 1–5 (2022).

104. D. Kumari, Y. Singh, S. Singh, V. Dogra, A. K. Srivastava, S. Srivastava, I. Garg, M. Bargotya, J. Hussain, L. Ganju, R. Varshney, Mitochondrial pathogenic mutations and metabolic alterations associated with COVID-19 disease severity. *J. Med. Virol.* **95**, e28553 (2023).

105. E. Dirican, Ş. T. Savrun, İ. E. Aydın, G. Gülbay, Ü. Karaman, Analysis of mitochondrial DNA cytochrome-b (CYB) and ATPase-6 gene mutations in COVID-19 patients. *J. Med. Virol.* **94**, 3138–3146 (2022).

106. C. Pizzamiglio, P. M. Machado, R. H. Thomas, G. S. Gorman, R. McFarland, M. G. Hanna, R. D. S. Pitceathly, on behalf of the MitoCOVID-19 Study Group, COVID-19-related outcomes in primary mitochondrial diseases: An internationsal study. *Neurology* **98**, 576–582 (2022).

107. M. Foretz, B. Guigas, B. Viollet, Metformin: Update on mechanisms of action and repurposing potential. *Nat. Rev. Endocrinol.* **19**, 460–476 (2023).

108. K. Khunti, P. Knighton, F. Zaccardi, C. Bakhai, E. Barron, N. Holman, P. Kar, C. Meace, N. Sattar, S. Sharp, N. J. Wareham, A. Weaver, E. Woch, B. Young, J. Valabhji, Prescription of glucose-lowering therapies and risk of COVID-19 mortality in people with type 2 diabetes: A nationwide observational study in England. *Lancet Diabetes Endocrinol.* **9**, 293–303 (2021).

109. C. T. Bramante, N. E. Ingraham, T. A. Murray, S. Marmor, S. Hovertsen, J. Gronski, C. McNeil, R. Feng, G. Guzman, N. Abdelwahab, S. King, L. Tamariz, T. Meehan, K. M. Pendleton, B. Benson, D. Vojta, C. J. Tignanelli, Metformin and risk of mortality in patients hospitalised with COVID-19: A retrospective cohort analysis. *Lancet Healthy Longev.* **2**, e34–e41 (2021).

110. J. D. Lalau, A. Al-Salameh, S. Hadjadj, T. Goronflot, N. Wiernsperger, M. Pichelin, I. Allix, C. Amadou, O. Bourron, T. Duriez, J.-F. Gautier, A. Dutour, C. Gonfroy, D. Gouet, M. Joubert, I. Julier, E. Larger, L. Marchand, M. Marre, L. Meyer, F. Olivier, G. Prevost, P. Quiniou, C. Raffaitin-Cardin, R. Roussel, P. J. Saulnier, D. Seret-Begue, C. Thivolet, C. Vatier, R. Desailloud, M. Wargny, P. Gourdy, B. Cariou, CORONADO investigators,

Metformin use is associated with a reduced risk of mortality in patients with diabetes hospitalised for COVID-19. *Diabetes Metab.* **47**, 101216 (2021).

111. H. E. Davis, L. McCorkell, J. M. Vogel, E. J. Topol, Long COVID: Major findings, mechanisms and recommendations. *Nat. Rev. Microbiol.* **21**, 133–146 (2023).

112. B. Appelman, B. T. Charlton, R. P. Goulding, T. J. Kerkhoff, E. A. Breedveld, W. Noort, C. Offringa, F. W. Bloemers, M. van Weeghel, B. V. Schomakers, P. Coelho, J. J. Posthuma, E. Aronica, W. Joost Wiersinga, M. van Vugt, R. C. I. Wüst, Muscle abnormalities worsen after post-exertional malaise in long COVID. *Nat. Commun.* **15**, 17 (2024).

113. C. T. Bramante, J. D. Huling, C. J. Tignanelli, J. B. Buse, D. M. Liebovitz, J. M. Nicklas, K. Cohen, M. A. Puskarich, H. K. Belani, J. L. Proper, L. K. Siegel, N. R. Klatt, D. J. Odde, D. G. Luke, B. Anderson, A. B. Karger, N. E. Ingraham, K. M. Hartman, V. Rao, A. A. Hagen, B. Patel, S. L. Fenno, N. Avula, N. V. Reddy, S. M. Erickson, S. Lindberg, R. Fricton, S. Lee, A. Zaman, H. G. Saveraid, W. J. Tordsen, M. F. Pullen, M. Biros, N. E. Sherwood, J. L. Thompson, D. R. Boulware, T. A. Murray, COVID-OUT Trial Team, Randomized trial of metformin, ivermectin, and fluvoxamine for Covid-19. *N. Engl. J. Med.* **387**, 599–610 (2022).

114. H. Parthasarathy, D. Tandel, A. H. Siddiqui, K. H. Harshan, Metformin suppresses SARS-CoV-2 in cell culture. *Virus Res.* **323**, 199010 (2023).

115. C. T. Bramante, K. B. Beckman, T. Mehta, A. B. Karger, D. J. Odde, C. J. Tignanelli, J. B. Buse, D. M. Johnson, R. H. B. Watson, J. J. Daniel, D. M. Liebovitz, J. M. Nicklas, K. Cohen, M. A. Puskarich, H. K. Belani, L. K. Siegel, N. R. Klatt, B. Anderson, K. M. Hartman, V. Rao, A. A. Hagen, B. Patel, S. L. Fenno, N. Avula, N. V. Reddy, S. M. Erickson, R. D. Fricton, S. Lee, G. Griffiths, M. F. Pullen, J. L. Thompson, N. E. Sherwood, T. A. Murray, M. R. Rose, D. R. Boulware, J. D. Huling, COVID-OUT Study Team, Favorable antiviral effect of metformin on SARS-CoV-2 viral load in a randomized, placebo-controlled clinical trial of COVID-19. *Clin. Infect. Dis.* **79**, 354–363 (2024).

116. M. P. King, G. Attardi, Isolation of human cell lines lacking mitochondrial DNA. *Methods Enzymol.* **264**, 304–313 (1996).

117. I. A. Trounce, Y. L. Kim, A. S. Jun, D. C. Wallace, Assessment of mitochondrial oxidative phosphorylation in patient muscle biopsies, lymphoblasts, and transmitochondrial cell lines. *Methods Enzymol.* **264**, 484–509 (1996).

118. M. Picard, J. Zhang, S. Hancock, O. Derbeneva, R. Golhar, P. Golik, S. O'Hearn, S. Levy, P. Potluri, M. Lvova, A. Davila, C. S. Lin, J. C. Perin, E. F. Rappaport, H. Hakonarson, I. A. Trounce, V. Procaccio, D. C. Wallace, Progressive increase in mtDNA 3243A>G heteroplasmy causes abrupt transcriptional reprogramming. *Proc. Natl. Acad. Sci. U.S.A.* **111**, E4033–E4042 (2014).

119. S. Horibe, K. Ishikawa, K. Nakada, M. Wake, N. Takeda, T. Tanaka, S. Kawauchi, N. Sasaki, Y. Rikitake, Mitochondrial DNA mutations are involved in the acquisition of cisplatin resistance in human lung cancer A549 cells. *Oncol. Rep.* **47**, 32 (2022).

120. C. A. Schneider, W. S. Rasband, K. W. Eliceiri, NIH Image to ImageJ: 25 years of image analysis. *Nat. Methods* **9**, 671–675 (2012).

121. J. Schindelin, I. Arganda-Carreras, E. Frise, V. Kaynig, M. Longair, T. Pietzsch, S. Preibisch, C. Rueden, S. Saalfeld, B. Schmid, J. Y. Tinevez, D. J. White, V. Hartenstein, K. Eliceiri, P. Tomancak, A. Cardona, Fiji: An open-source platform for biological-image analysis. *Nat. Methods* **9**, 676–682 (2012).

122. P. M. Schaefer, L. Scherer Alves, M. Lvova, J. Huang, K. Rathi, K. Janssen, A. Butic, T. Yardeni, R. Morrow, M. Lott, D. Murdock, A. Song, K. Keller, B. A. Garcia, C. A. Francomano, D. C. Wallace, Combination of common mtDNA variants results in mitochondrial dysfunction and a connective tissue dysregulation. *Proc. Natl. Acad. Sci. U.S.A.* **119**, e2212417119 (2022).

123. M. T. Lott, J. N. Leipzig, O. Derbeneva, H. M. Xie, D. Chalkia, M. Sarmady, V. Procaccio, D. C. Wallace, mtDNA variation and analysis using mitomap and mitomaster. *Curr. Protoc. Bioinformatics* **44**, 1.23.1–1.23.26 (2013).

124. B. Howe, A. Umrigar, F. Tsien, Chromosome preparation from cultured cells. *J. Vis. Exp.* **83**, e50203 (2014).

125. U. Singh, J. Li, A. Seetharam, E. S. Wurtele, pyrpipe: A Python package for RNA-Seq workflows. *NAR Genom. Bioinform.* **3**, lqab049 (2021).

126. R. Patro, G. Duggal, M. I. Love, R. A. Irizarry, C. Kingsford, Salmon provides fast and bias-aware quantification of transcript expression. *Nat. Methods* **14**, 417–419 (2017).

127. M. I. Love, W. Huber, S. Anders, Moderated estimation of fold change and dispersion for RNA-seq data with DESeq2. *Genome Biol.* **15**, 550 (2014).

128. G. Yu, L. G. Wang, Y. Han, Q. Y. He, clusterProfiler: An R package for comparing biological themes among gene clusters. *OMICS* **16**, 284–287 (2012).

129. A. Liberzon, C. Birger, H. Thorvaldsdóttir, M. Ghandi, J. P. Mesirov, P. Tamayo, The Molecular Signatures Database (MSigDB) hallmark gene set collection. *Cell Syst.* **1**, 417–425 (2015).

130. V. Sharma, labelStackFromATextFile.ijm, https://gist.github.com/ved-sharma/8b6ff203af59827086f1cc1e0775f6d7.

131. D. R. Stirling, M. J. Swain-Bowden, A. M. Lucas, A. E. Carpenter, B. A. Cimini, A. Goodman, CellProfiler 4: Improvements in speed, utility and usability. *BMC Bioinformatics* **22**, 433 (2021).
